# Theoretical Models for Surface Forces and Adhesion and Their Measurement Using Atomic Force Microscopy

**DOI:** 10.3390/ijms131012773

**Published:** 2012-10-08

**Authors:** Fabio L. Leite, Carolina C. Bueno, Alessandra L. Da Róz, Ervino C. Ziemath, Osvaldo N. Oliveira

**Affiliations:** 1 Nanoneurobiophysics Research Group, Department of Physics, Chemistry and Mathematics, Federal University of São Carlos (UFSCar), P.O. Box 3031, CEP 18052-780, Sorocaba, São Paulo, Brazil; E-Mails:carolcastrob@gmail.com (C.C.B.); alessandra.roz@gmail.com (A.L.D.R.); 2 Institute of Geosciences and Exact Sciences, São Paulo State University (UNESP), P.O. Box 178, CEP 13550-970, Rio Claro, São Paulo, Brazil; E-Mail: ziemath@rc.unesp.br; 3 Institute of Physics of São Carlos, University of São Paulo (USP), P.O. Box 369, CEP 13560-970, São Carlos, São Paulo, Brazil; E-Mail: chu@ifsc.usp.br

**Keywords:** van der Waals, adhesion, surface forces, atomic force microscopy, atomic force spectroscopy, structural forces, double-layer, wettability, AFM

## Abstract

The increasing importance of studies on soft matter and their impact on new technologies, including those associated with nanotechnology, has brought intermolecular and surface forces to the forefront of physics and materials science, for these are the prevailing forces in micro and nanosystems. With experimental methods such as the atomic force spectroscopy (AFS), it is now possible to measure these forces accurately, in addition to providing information on local material properties such as elasticity, hardness and adhesion. This review provides the theoretical and experimental background of AFS, adhesion forces, intermolecular interactions and surface forces in air, vacuum and in solution.

## 1. Introduction

The integral form of interaction forces between surfaces of macroscopic bodies through a third medium (e.g., vacuum and vapor) are named *surfaces forces*, while those that work to hold two bodies in contact are named *adhesive forces*. If a process between two bodies is perfectly elastic, that is, no energy dissipates during their interaction, the adhesive and surface forces are equal in magnitude [1]. Understanding intermolecular interactions is key to achieving control of materials at the molecular level, which is essential for various areas of physics and for nanotechnology. While the properties of widely used materials such as metals and inorganic semiconductors are governed by covalent or metallic bonding, in soft matter van der Waals (vdW) interactions play a prominent role, though their associated energies are much smaller than covalent or even H-bonds. In most systems involving surfaces and colloidal phenomena, intermolecular (or interatomic) forces are crucial, which act between discrete, nonbonded atoms or molecules over distances significantly greater than molecular bond dimensions [2]. Intermolecular forces are, therefore, nondirectional, nonstoichiometric, and long-range forces [3]. The vdW forces arise from interaction between uncharged atoms or molecules, leading not only to such phenomena as the cohesion of condensed phases and physical adsorption of gases, but also to a universal force of attraction between macroscopic bodies [3,4]. The existence of this force is demonstrated by the adherence of any particles of microscopic size to one another in the absence of special forces of repulsion, as in aggregation of fine powders and coagulation in colloidal dispersions [5].

vdW forces are crucial in a host of phenomena such as adhesion [6], surface tension [7], nanostructured films [8], nanostructures [9,10], nanosystems [11], physical adsorption [12] and wettability [13], and affect the properties of gases, liquids, thin films and the structure of condensed macromolecules [14–16]. They are also relevant in determining film thickness in wetting [17] and surface melting problems [18], interactions involving polymer surfaces [14,19], in free standing films in soap bubbles [20], and in the flocculation and deflocculation of colloidal systems [21]. Direct information of vdW forces became possible in the 1970’s with the surface force apparatus (SFA) [22,23] to explore their magnitude and distance dependence. However, this technique is restricted because it requires smooth, semitransparent, macroscopic surfaces as part of the experimental set-up, which limits the materials that can be examined.

A significant advance was achieved with the atomic force microscopy (AFM) [24–26] which made it possible to observe and manipulate molecular and atomic level features (*i.e.*, measurement and manipulation of vdW forces), in addition to having a well-defined tip-sample geometry with a higher force resolution [27]. Another major application of AFM is force spectroscopy [28,29] where the AFM tip is extended towards and retracted from the surface, and the deflection of the cantilever is monitored as a function of the piezoelectric displacement. AFS has been used to measure nanoscale contacts, atomic bonding, vdW and Casimir forces, dissolution forces in liquids, single molecule stretching and rupture forces [8,28,30,31]. It is ideally suited for short and long-range interactions as well as to study adhesion between solid surfaces, including those bearing deposited polyelectrolytes [32,33]. The forces relevant to AFM are ultimately of electromagnetic origin, but distinct intermolecular, surface and macroscopic effects give rise to interactions with different dependencies. In the absence of external fields, the dominant forces are vdW interactions, short-range repulsive interactions and adhesion forces. Following Butt [28] and Ducker *et al.* [30], vdW forces between AFM tips and surfaces have been calculated and measured by many researchers [34–39] based on the interaction between a spherical or conical tip and various types of samples.

The aim of this paper is to provide a review of vdW interactions and adhesion forces, including fundamental models and possible applications with AFM. The paper is organized as follows. The basic concepts involved in vdW interactions and use of the Hamaker constant are introduced in Sections 2 and 3, respectively. The main uses of AFS are discussed in Section 4. Section 5 is dedicated to theoretical models and experimental results for vdW and adhesion forces in vacuum, in air and in solution, where the measurements using pull-on and pull-off forces are treated separately. Section 6 closes the paper with final remarks.

## 2. Van der Waals Interactions

### 2.1. vdW Interactions between Molecules in Vacuum

In chemistry and physics, the name vdW force is sometimes used as a synonym for the totality of non-covalent forces (also known as intermolecular forces). These forces, which act between stable molecules, are weak compared to those appearing in chemical bonding [40]. All atoms and molecules, even in an inert gas such as helium and argon, exhibit weak, short-range attractions due to vdW forces. Friction, surface tension, viscosity, adhesion and cohesion, are also related to vdW forces [41,42]. These phenomena arise from the fluctuations in the electric dipole moments of molecules which become correlated as the molecules come closer together, giving rise to an attractive force [43]. In 1893, Johannes D. van der Waals (1837–1923) [44] developed a thermodynamic theory of capillarity to explain the behavior of liquids, after having introduced unspecific forces for gas molecules. He established the minimization of free energy as the criterion for equilibrium in a liquid-gas system and applied this to surface tensions, introducing the long-range vdW forces as resulting from dipole and quadrupolar interactions between molecules that make up gases, liquids or solids [45]. vdW forces are the general name given to a set of forces characterized by the same power dependence on distance, having the dipole moment and the atomic polarizability as the important parameters [46]. They include three forces of different origins [47], all proportional to 1/*r*^6^, where *r* is the distance between the atoms or molecules.

**Figure 1:**
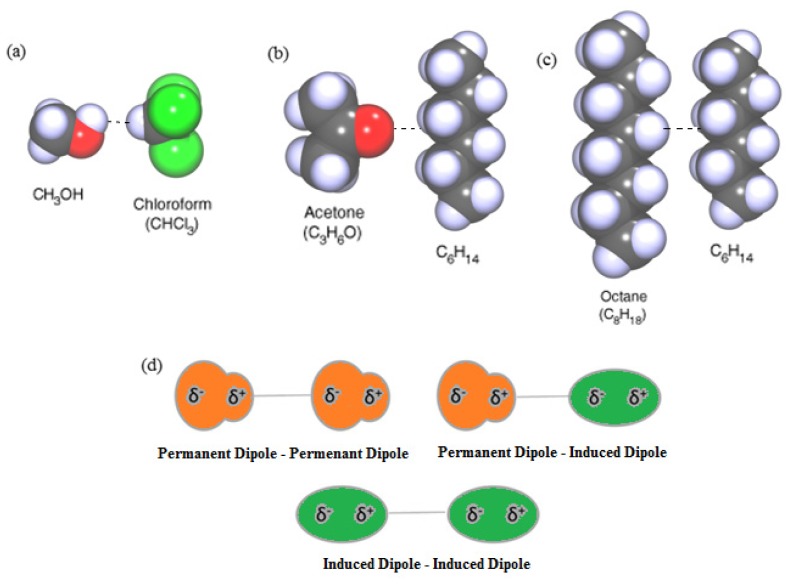
(**a**) Permanent Dipole-Permanent Dipole or Keesom forces. They exist only between polar molecules, being stronger than London forces for molecules of equivalent size; (**b**) Permanent Dipole-Induced Dipole or Debye force. It arises from the distortion of the charge cloud induced by a polar molecule nearby, *i.e.*, a non-polar molecule will be temporarily polarized in the vicinity of a polar molecule, and the induced and permanent dipoles will be mutually attracted; (**c**) Instantaneous Dipole-Induced Dipole or London forces. They result from electrostatic attraction between temporary dipoles and induced dipoles caused by movement of electrons; these are attraction forces that operate between all molecules and among isolated atoms in noble gases. The strength of the forces is related to the number of electrons present and hence to the size of the molecule (or isolated atom); **(d)** Interactions between molecules–temporary and permanent dipoles.

The first contribution is due to electrostatic interactions between charges (in molecular ions), dipoles (for polar molecules), quadrupoles (all molecules with symmetry lower than cubic), and permanent multipoles. It is also referred to as *Keesom force*, named after Willem Hendrik Keesom [48]. Often, these forces occur between two molecules with permanent dipoles, *i.e.*, they originate from the attraction between permanent dipoles as in the molecules of Figure 1a, and are temperature dependent. Such molecules are called *polar*, e.g., water, which has a dipole moment of 1.85 Debye (1D = 3.336 × 10^−30^ C/m). These permanent dipoles occur when 2 atoms in a molecule have substantially different electronegativity (Figure 1d). Thus, Keesom forces depend on the electronegativity of an atom [49], and more electronegative atoms have a *δ*^−^ charge. Note that usually the dipole-dipole interaction between two atoms is zero, because atoms rarely carry a permanent dipole. Atoms in an *S*-state (a spherically symmetric state), such as the H-atom and noble gas atoms, do not carry any multipole and for such systems this force is absent.

The second contribution is the *induction* (also known as polarization) or *Debye force* [50], arising from interactions between rotating permanent dipoles and from the polarizability of atoms and molecules (induced dipoles). These induced dipoles occur when one molecule with a permanent dipole repels another molecule’s electrons. A molecule with permanent dipole can induce a dipole in a similar neighboring molecule and cause mutual attraction, as depicted in Figure 1b. Debye forces cannot occur between atoms. The forces between induced and permanent dipoles are not as temperature dependent as Keesom interactions because the induced dipole is free to shift and rotate around the non-polar molecule. The Debye induction effects and Keesom orientation effects are referred to as polar interactions.

The third and dominant contribution is the dispersion or *London force* (fluctuating dipole-induced dipole) [51], due to the non-zero instantaneous dipole moments of all atoms and molecules. Such polarization can be induced either by a polar molecule or by the repulsion of negatively charged electron clouds in non-polar molecules (Figure 1c). Thus, London interactions are caused by random fluctuations in electron density in an electron cloud. Figure 1d shows that the electron rich side, possessing a δ^−^ charge, and the electron deficient side (with a δ^+^ charge) attract and repel neighboring dipoles. An atom with a large number of electrons will have a greater associated London force than a smaller atom. The dispersion (London) force is the most important component because all materials are polarizable, whereas Keesom and Debye forces require permanent dipoles. The London interaction is universal and is present in atom-atom interactions as well. For various reasons, London interactions (dispersion) have been considered relevant for interactions between macroscopic bodies in condensed systems. Hamaker developed the theory of vdW between macroscopic bodies in 1937 and showed that the additivity of these interactions renders them considerably more long-range, as will be seen in Section 3.

The interaction free energies of these 3 types of forces can be written in a single equation, which describes the total vdW contribution to the free energy of interaction between two small particles 1 and 2:

(2.1) $$w(r)=-\frac{C_{12}}{r^{6}}=\frac{C_{ind}+C_{orient}+C_{disp}}{r^{6}}=-\frac{C_{vdW}}{r^{6}}$$

where *C_ind_*, *C_orient_*, and *C_disp_* are the coefficients due to induction, orientation and dispersion, respectively. This equation together with a repulsive [52], very short-ranged potential due to the overlap between the electron clouds of atoms describes the interaction between isolated molecules in vacuum.

The constant *C_vdW_* (C^(6)^) in Equation 2.1 is given by [53]:

(2.2) $$C_{vdW}=C_{\nu =0}+C_{\nu >0}={\left[-\frac{3k_{B}T}{{\left(4\pi {ɛ}_{\circ }ɛ\right)}^{2}}\left(\frac{\mu_{1}^{2}}{3k_{B}T}+\alpha_{1}\right)\left(\frac{\mu_{2}^{2}}{3k_{B}T}+\alpha_{2}\right)\right]}_{\nu =0}-{\left[\frac{3\alpha_{2}\alpha_{1}}{2{\left(4\pi {ɛ}_{\circ }ɛ\right)}^{2}}\frac{\text{h}\nu_{1}\nu_{2}}{\nu_{1}+\nu_{2}}\right]}_{\nu >0}$$

and thus:

(2.3) $${\text{C}}_{\text{vdW}}=-{\left[\frac{\mu_{1}^{2}\mu_{2}^{2}}{3{\left(4\pi {ɛ}_{0}ɛ\right)}^{2}{\text{k}}_{\text{B}}\text{T}}+\frac{\mu_{1}^{2}\alpha_{2}+\mu_{2}^{2}\alpha_{1}}{{\left(4\pi {ɛ}_{0}ɛ\right)}^{2}}\right]}_{\nu =0}+{\left[\frac{3\alpha_{2}\alpha_{1}}{2{\left(4\pi {ɛ}_{0}ɛ\right)}^{2}}\frac{\text{h}\nu_{1}\nu_{2}}{\nu_{1}+\nu_{2}}\right]}_{\nu >0}$$

in which α_1_ and α_2_ are the electronic polarizabilities of the molecules, μ_1_ and μ_2_ are the dipole moments, ɛ_0_ is the vacuum permittivity (8.854 × 10^−12^ C^2^J^−1^m^−1^), *k_B_* is the Boltzmann constant (1.381 × 10^−23^ JK^−1^), *T* is the temperature, *h*ν_1_ and *h*ν_2_ are the first ionization potentials of the molecules (*ν*_1_, *ν*_2_ are ionization frequencies (Hz)) and *h* is the Planck constant (6.626 × 10^−34^ Js). The first term (ν = 0) contains the Keesom and Debye energies, valid for interactions between polar molecules and named polar or entropic contribution [54–56]. The second term (ν > 0) is referred to as dispersion (London) contribution and acts between all molecules [57]. This expression yields good agreement with experimental data, with *C_12_* being close to the average of *C_11_* and *C_22_* for dissimilar molecules 1 and 2. However, this empirical law does not apply to water, which is highly polarized [53].

The total vdW interaction free energy of two molecules 1 and 2 in free space or air, where ɛ = 1, is therefore:

(2.4) $$w(r)=-\frac{1}{{\text{r}}^{6}}\left\{{\left[\frac{\mu_{1}^{2}\mu_{2}^{2}}{3{\left(4\pi {ɛ}_{0}ɛ\right)}^{2}{\text{k}}_{\text{B}}\text{T}}+\frac{\mu_{1}^{2}\alpha_{2}+\mu_{2}^{2}\alpha_{1}}{{\left(4\pi {ɛ}_{0}ɛ\right)}^{2}}\right]}_{\nu =0}+{\left[\frac{3\alpha_{2}\alpha_{1}}{2{\left(4\pi {ɛ}_{0}ɛ\right)}^{2}}\frac{h\nu_{1}\nu_{2}}{\nu_{1}+\nu_{2}}\right]}_{\nu >0}\right\}$$

The interaction (Keesom forces) between molecules with permanent dipole moments (polar molecules) (first term) also contain dipole-quadrupole interactions, but these contributions are usually much weaker. The quadrupolar contribution to the atom-surface vdW interaction was discussed by Jiang *et al.* [58,59] and by Hutson *et al*. [60]. When molecular constituents of a gas (N_2_, O_2_ or H_2_) have no dipole-allowed vibration-rotation transitions, no absorption is seen using conventional spectroscopic techniques [61,62]. However, long-path-length infrared spectroscopy can reveal weak features in the neighborhood of the fundamental vibration of the uncomplexed molecule [63]. The spectra of N_2_-Ar molecule near fundamental vibration of N_2_ can also present absorption bands [64]. This phenomenon occurs because the complex contains a small dipole moment induced by the electron cloud distortion and molecular quadrupole induction effects [62]. Power and Thirunamachandran [65] concluded that the internal structures of the interacting systems need to be considered to obtain the complete form of vdW forces in terms of the multipole polarizabilities. The retarded vdW forces involving electric quadrupole polarizabilities have also been investigated in detail [66,67].

The vdW dipole-dipole interaction potential can be calculated using the London’s formula (Equation 2.1). The dipole-quadrupole vdW potential and dipole-quadrupole coefficients have been estimated by Mayer [68] using a revised formula by Margenau [69]. The intervening matrix elements appearing in the Margenau formula were expressed by Mayer in terms of polarizabilities, thus obtaining [69–71]:

(2.5) $$w(r)=-\frac{{\text{C}}^{\left(8\right)}}{r^{8}}$$

where C^(8)^ denotes the dipole-quadrupole constant and is described in terms of dispersion coefficients (C^(6)^). For more details see Khandelwal *et al.* [71]. Using Equation 2.5, Porsev and Derevianko [72] calculated vdW coefficients C^(6)^ and C^(8)^ of alkaline-earth metal dimers for the interaction of two like atoms in the ground state with high accuracy. This expression is easily extended to include quadrupole-quadrupole dispersion energies as [65]:

(2.6) $$w(r)=-\frac{{\text{C}}^{\left(10\right)}}{r^{10}}$$

The main problem of Equation 2.1 is that it is incomplete, since it neglects quadrupolar polarizations and such effects make an important contribution to C^(6)^. This may be seen most easily by considering the case of a solid A composed of distinct polarizable atoms B. If dielectric screening (local field) effects are neglected, the atom-surface potential may be written as a sum over long-range atom-atom potential [60]:

(2.7) $$w(r)=-\frac{{\text{C}}^{\left(6\right)}}{r^{6}}-\frac{{\text{C}}^{\left(8\right)}}{r^{8}}-\frac{{\text{C}}^{\left(10\right)}}{r^{10}}$$

Thus, vdW forces between atoms without permanent dipoles may be described as resulting from the interactions of multipoles associated with quantum transitions of the atoms. When the atoms are far apart, the dipole interaction is the only appreciable one. But at short distances, higher dipoles, usually neglected, must be considered [69]. For more details on the role of quadrupole forces in vdW attractions, see relevant papers [58–60,73]. Pauling and Beach [74] calculated the vdW interaction energy of two hydrogen atoms at large internuclear distances using a linear variation function. The authors use 26 terms for the dipole-dipole interaction, 17 for the dipole-quadrupole interaction and 26 for the quadrupole-quadrupole interaction. The interaction energy was given as:

(2.8) $$w=-\frac{6.49903{\text{e}}^{2}}{a_{o}\rho^{6}}-\frac{124.399{\text{e}}^{2}}{a_{o}\rho^{8}}-\frac{1135.21{\text{e}}^{2}}{a_{o}\rho^{10}}$$

where $\rho =\frac{r}{a_{o}}$.

Summarizing, almost all intermolecular forces have four major contributions. There is always a repulsive part, prohibiting the collapse of molecular complexes, and an attractive part. The repulsive part is mainly due to the typical quantum mechanical effect of intermolecular electron exchange. The attractive part consists of three distinct contributions, namely Keesom, Debye and London. It is noteworthy that in some texts the vdW force means the totality of forces (including repulsion), in others it means all the attractive forces, while still in some other cases the term vdW is used solely as a synonym for the London force.

### 2.2. vdW between Molecules in a Medium

The vdW force can be calculated also for atoms or molecules of dielectric constant ɛ_1_ and ɛ_2_ in a medium of dielectric constant ɛ_3_. McLachlan [75] presented a generalized theory for vdW forces between infinite media 1 and 2 separated by a medium 3, in which α was the excess polarizability of the molecules. For a small spherical molecule 1 of radius α in a medium 3, the excess polarizability is given approximately by [23,76]:

(2.9) $$\alpha_{exc}(\nu )=4\pi {ɛ}_{\circ }{ɛ}_{3}(\nu )\left(\frac{{ɛ}_{1}(\nu )-{ɛ}_{3}(\nu )}{{ɛ}_{1}(\nu )+2{ɛ}_{3}(\nu )}\right)$$

where ɛ_1_ and ɛ_2_ are the dielectric constants of media 1 and 2, respectively, and ρ is the number of atoms per unit volume.

Inserting Equation 2.3 into Equation 2.2, the entropic (*ν* = 0) and the dispersion (*ν* > 0) terms become:

(2.10) $$C_{\nu =0}=-3k_{B}T\rho_{1}\rho_{2}\left(\frac{{ɛ}_{1}(0)-{ɛ}_{3}(0)}{{ɛ}_{1}(0)+2{ɛ}_{3}(0)}\right)\left(\frac{{ɛ}_{2}(0)-{ɛ}_{3}(0)}{{ɛ}_{2}(0)+2{ɛ}_{3}(0)}\right)$$

and

(2.11) $$C_{\nu >0}=-\frac{3h\rho_{1}\rho_{2}}{\pi }\int_{0}^{\infty }\left(\frac{{ɛ}_{1}(i\nu )-{ɛ}_{3}(i\nu )}{{ɛ}_{1}(i\nu )+2{ɛ}_{3}(i\nu )}\right)\left(\frac{{ɛ}_{2}(i\nu )-{ɛ}_{3}(i\nu )}{{ɛ}_{2}(i\nu )+2{ɛ}_{3}(i\nu )}\right)\text{d}\nu$$

in which ɛ_1_(0), ɛ_2_(0), and ɛ_3_(0) are the static dielectric constants of the three media, and ɛ_1_(*iv*), ɛ_2_(*iv*), and ɛ_3_(*iv*) are the dielectric constants of the three media at the imaginary frequencies *iv* = 2iπ*k_B_T*/*h*. If the dielectric medium has one strong absorption peak at the frequency ν*_e_* (mean absorption frequency in the ultraviolet region), which is usually different from the frequency ν of the isolated molecule, ɛ_1_(*v*) can be written as ɛ (*v*) = 1 + (*n*^2^ − 1)/[1 − (*v*/*v_e_*)^2^], so that:

(2.12) $$ɛ(i\nu )=1+\frac{n^{2}-1}{1+{\left(\nu /\nu_{e}\right)}^{2}}$$

where *n* is the refractive index, approximately equal to $\sqrt{ɛ(\nu_{\nu is})}$, where *v_vis_* = 5 × 10^14^ s^−1^. Substituting into Equation 2.7, we obtain:

(2.13) $$C_{\nu >o}=-\frac{\sqrt{3}}{2}h\nu_{e}\rho_{1}\rho_{2}\left[\frac{\left(n_{1}^{2}-n_{3}^{2}\right)\left(n_{2}^{2}-n_{3}^{2}\right)}{\sqrt{\left(n_{1}^{2}+2n_{3}^{2}\right)}\sqrt{\left(n_{2}^{2}-2n_{3}^{2}\right)}\left[\sqrt{\left(n_{1}^{2}+2n_{3}^{2}\right)}+\sqrt{\left(n_{2}^{2}+2n_{3}^{2}\right)}\right]}\right]$$

where *n*_1_, *n*_2_ and *n*_3_ are the refractive indices for media with molecules 1 and 2 and for the medium 3, respectively. For simplicity, it is assumed that all three media have the same absorption frequency *v_e_*.

The total vdW interaction free energy of two identical molecules 1 in medium 3 is therefore:

(2.14) $$w(r)=w{\left(r\right)}_{\nu =0}+w{\left(r\right)}_{\nu >0}\approx -\left[3k_{B}T{\left(\frac{{ɛ}_{1}(0)-{ɛ}_{3}(0)}{{ɛ}_{1}(0)+2{ɛ}_{3}(0)}\right)}^{2}+\frac{\sqrt{3}}{4}h\nu_{e}\frac{{\left(n_{1}^{2}-n_{3}^{2}\right)}^{2}}{{\left(n_{1}^{2}+2n_{3}^{2}\right)}^{3/2}}\right]\frac{a^{6}}{r^{6}},$$

which is strictly valid only for *r* >> *a*, where *a* is the radius of the molecule.

Some features of the vdW forces are noteworthy:

1. The vdW force is anisotropic, similarly to the polarizabilities of the majority of molecules, *i.e.*, they have different values for different molecular directions (except for ideal spherical particles);
2. The orienting effects of the anisotropic dispersion forces are usually less important than other forces such as dipole-dipole interactions;
3. The vdW force is non-additive. The force between two molecules is affected by molecules nearby, which behave like a medium, and is important for large particles interacting with a surface;
4. The vdW force is much reduced in a solvent medium.

Retardation effects should be considered to account for the speed with which particles interact (limited to the speed light), especially in media where the speed of light is much smaller than in vacuum [53].

## 3. Interactions between Surfaces and the Hamaker Constant

**Figure 2:**
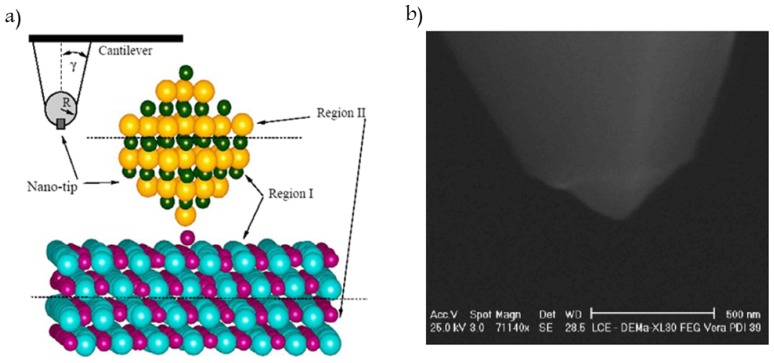
(**a**) Scheme for the integration of macroscopic and AFM tip (Reproduced by permission of IOP Publishing Ltd. [80]); (**b**) SEM image of a sharpened pyramidal tip (Reproduced by permission of Taylor and Francis Ltd. [81].

The expressions 2.1 through 2.11 are used for interactions between molecules and atoms isolated. For estimating vdW interactions between surfaces, measurements can be performed using an AFM (Figure 2) or theoretical models may be used. In general, there are two approaches to calculating the vdW interaction between two bodies as a function of their separation distance [77]. The first one, referred to as the Hamaker approach [78], determines the vdW interaction of two macroscopic bodies by carrying out the so-called Hamaker-type integration of all the intermolecular interactions. The second approach, based on the Lifshitz theory [79], is more rigorous and gives the vdW interaction energy as a function of macroscopic electrodynamic properties of the interacting media, such as their dielectric permittivities and refractive indices. It should be noted that, regardless of the approach employed, the only difference is in the way the Hamaker constant is determined.

The most direct way to investigate vdW interactions is simply to position two bodies together and measure the force of attraction as a function of the distance between them, as we shall discuss in Section 4. Interactions between surfaces in AFM or SFA may be modeled by summing the attractive and repulsive potential pairs over all interacting atoms. The potential energy is then:

(3.1) $$w(r)=\xi \left({\left(\frac{r_{o}}{r}\right)}^{12}-2{\left(\frac{r_{o}}{r}\right)}^{6}\right)$$

where ξ is the binding energy (well depth), *r_0_* is approximately the equilibrium distance between bound atoms (*r_o_* = 2^⅙^σ, with σ being the diameter of one of the atoms) and *r* is the interatomic distance. For clarity, the potential can also be expressed as:

(3.2) $$w(r)=4\xi \left({\left(\frac{\sigma }{r}\right)}^{12}-{\left(\frac{\sigma }{r}\right)}^{6}\right)$$

The tip-surface interactions are normally modeled as schematically shown in Figure 2a [80]. The AFM tip is represented by a conical macroscopic tip of angle ϕ with a sphere of radius *R* at the end, according to the pyramidal shape determined with scanning electron microscopy (SEM) (Figure 2b) [81]. The atomistic nano-tip is embedded at the base of the sphere.

A simple sum for all the atoms of the tip and sample is a good approximation for repulsive forces. However, the vdW interaction is non-additive; *i.e.*, the interaction of two bodies is affected by the presence of other bodies, and a simple sum of the pair-wise interactions is usually greater than the actual force between the macro bodies [39]. The degree of non-additivity may depend on the density of the medium, *i.e*., for rarefied media it is possible to assume additive forces [82]. An additive approximation based on the local geometry, material properties and structure of the tip [83] is used in many practical applications, including atomistic simulations for AFM [84], because the full tip contains billions of atoms.

The original work by London [85] focused on the attractions of induced electrical dipoles in individual atoms and molecules. Sokolov [84] generalized this for the forces between spherical particles as a function of particle size and separation, by summing all dipolar interactions of the atoms and molecules of a solid or liquid. He was able to apply this analysis for particles in a fluid, thus introducing constants (now known as Hamaker constants), which provide scales for the vdW forces between particles of various shapes with intervening media. Hamaker used the following approximations: (1) the total interaction is obtained by the pair-wise summation of the individual contributions (additivity); (2) the summation can be replaced by an integration over the volumes of the interacting bodies assuming that each atom occupied a specific volume, with a density ρ (continuous medium); (3) ρ and *C* (interaction constant defined by London and specific to the identity of the interacting atoms) should be uniform over the volume of the bodies (homogeneous material properties).

The non-retarded energy of interaction between two particles 1 and 2, of volumes *V*_1_ and *V*_2_ containing ρ_1_ and ρ_2_ atoms per milliliter is:

(3.3) $$w=-\int_{V_{1}}\text{d}\nu_{1}\int_{V_{2}}\text{d}\nu_{2}\frac{\rho_{1}\rho_{2}C_{L}}{D^{6}}$$

where *C_L_* is the non-retarded microscopic constant (London constant), *i.e.*, the second term in Equation 2.2, *C_ν_*_>0_. The vdW force is

(3.4) $$F_{vdW}=\frac{\partial w}{\partial D}$$

vdW forces have been obtained by combining Equation 3.3 with Equation 3.4 for bodies of regular geometric form [78]. For example, for two spheres of radii *R*_1_ and *R*_2_, $F_{vdW}=\frac{AR}{12D^{2}}$ (see Section 5), where *A* is the Hamaker’s coefficient (or constant), being equal to [78]:

(3.5) $$A=\pi^{2}C_{L}\rho_{1}\rho_{2}$$

The Hamaker constant depends on *C_L_*, a microscopic property of two interacting atoms, then ultimately depending on the strength of the interaction between bodies and the medium surrounding them. It has dimension of energy.

In Equation 3.5, many-body effects of an intervening liquid medium and retardation effects for large distances were ignored. To overcome this problem, Lifshitz [79] presented an approach with multi-body interaction where the polarizability, *α*, and the first ionization potential in the Hamaker equations were replaced by the static and frequency-dependent dielectric constant, ɛ*_r_*, and refractive index, *n* [86]. Within Lifshitz’ derivation, the Hamaker constant for interaction of medium 1 and 2 across medium 3 (immersion medium) is:

(3.6) $$A≅\frac{3}{4}k_{B}T\frac{{ɛ}_{1}-{ɛ}_{3}}{{ɛ}_{1}+{ɛ}_{3}}\frac{{ɛ}_{2}-{ɛ}_{3}}{{ɛ}_{2}+{ɛ}_{3}}+\frac{3h}{4\pi }\int_{\nu 1}^{\infty }\left(\frac{{ɛ}_{1}(i\nu )-{ɛ}_{3}(i\nu )}{{ɛ}_{1}(i\nu )+{ɛ}_{3}(i\nu )}\right)\left(\frac{{ɛ}_{21}(i\nu )-{ɛ}_{3}(i\nu )}{{ɛ}_{2}(i\nu )+{ɛ}_{3}(i\nu )}\right)\text{d}\nu$$

Inserting Equation 2.9 into Equation 3.6, the Hamaker constant may be expressed in terms of dielectric constants, *ɛ_r_*, and refractive index, *n*_r_, taking into account the approximation of Ninham and Parsegian [20] and Hough and White [87]:

(3.7) $$A≅\frac{3}{4}k_{B}T\frac{{ɛ}_{1}-{ɛ}_{3}}{{ɛ}_{1}+{ɛ}_{3}}\frac{{ɛ}_{2}-{ɛ}_{3}}{{ɛ}_{2}+{ɛ}_{3}}+\frac{3h\nu_{e}}{8\sqrt{2}}\frac{\left(n_{1}^{2}-n_{3}^{2})(n_{2}^{2}-n_{3}^{2}\right)}{\sqrt{n_{1}^{2}+n_{3}^{2}}\sqrt{n_{2}^{2}+n_{3}^{2}}\left[\sqrt{n_{1}^{2}+n_{3}^{2}}+\sqrt{n_{2}^{2}+n_{3}^{2}}\right]}$$

The first term of Equation 3.7 includes Debye and Keesom forces and the second term is the dispersion contribution. In polar condensed media, especially in the presence of electrolytes, the dispersion contribution is normally the only significant term.

For the “symmetric case” of two identical phases (ɛ_1_ = ɛ_2_ and *n*_1_ = *n*_2_) interacting across medium 3, Equation 3.7 reduces to the simple expression:

(3.8) $$A≅\frac{3}{4}k_{B}T{\left(\frac{{ɛ}_{1}-{ɛ}_{3}}{{ɛ}_{1}+{ɛ}_{3}}\right)}^{2}+\frac{3h\nu_{e}}{16\sqrt{2}}\frac{{\left(n_{1}^{2}-n_{3}^{2}\right)}^{2}}{{\left(n_{1}^{2}+n_{3}^{2}\right)}^{3/2}}$$

The non-retarded Hamaker constant comprises non-dispersion or entropic contribution (*A*_ν=0_) and dispersion (A_ν>0_) components, such that *A* = *A*_ν=0_ + *A*_ν>0_. For identical particles, the Lifshitz continuum theory can be used to estimate the integral parts:

(3.9) $$A_{\nu =0}=\frac{3}{4}k_{B}T{\left(\frac{{ɛ}_{1}-{ɛ}_{3}}{{ɛ}_{1}+{ɛ}_{3}}\right)}^{2}$$

(3.10) $$A_{\nu >0}=\frac{3h\nu_{e}}{16\sqrt{2}}\frac{{\left(n_{1}^{2}-n_{3}^{2}\right)}^{2}}{{\left(n_{1}^{2}+n_{3}^{2}\right)}^{3/2}}$$

The term *k_B_T* defines the interaction as being primarily entropic in nature with a maximum value of $\frac{3}{4}(k_{B}T)$, since $\left\{\frac{\left({ɛ}_{1}-{ɛ}_{3}\right)}{\left({ɛ}_{1}+{ɛ}_{3}\right)}\right\}\leq 1$. At *T* = 300 K, *k_B_T* ≈ 3 × 10^−23^ J, which is an order of magnitude less than the dispersion contribution. The actual difference between the two terms (dispersion and electrostatic) will be reduced by mathematical cancellations in the second (dispersion) term in Equation 3.7, but only rarely will the electrostatic contribution constitute the dominant factor in the total interaction.

Thus, for interactions between two hydrocarbon phases across a water film, the Hamaker constant is $A_{\nu >0}=\frac{3\left(6.63\times 10^{-34}\right)\left(3\times 10^{15}\right)}{16\sqrt{2}}\frac{{\left(1.41^{2}-1.33^{2}\right)}^{2}}{{\left(1.41^{2}+1.33^{2}\right)}^{3/2}}\approx 0.17\times 10^{-20}\text{J}$, where both of these liquids have roughly the same absorption frequency ν*_e_* ≈ 3.0 × 10^15^ s^−1^ [23], *h* = 6.63× 10^15^ Js and *n*_1_, *n*_2_, are the refractive indices of water and hydrocarbon, respectively. Concerning the zero-frequency contribution, water has a high static dielectric constant ɛ_1_ = 80, while hydrocarbons have a dielectric constant ɛ_1_ ≈ 2 [23]. The large difference between ɛ_1_ and ɛ_2_ leads to a large zero-frequency contribution to the Hamaker constant of $A_{\nu =0}=\frac{3}{4}\left(1.381\times 10^{23}\right)\left(300\right){\left(\frac{80-2}{80+2}\right)}^{2}\approx 0.28\times 10^{-20}\text{J}$, giving *A_total_* ≈ 0.45 × 10^−20^ J.

For interactions between conducting bodies such as metals, Equation 3.7 cannot be applied, since their static dielectric constant ɛ is infinite (the dielectric permittivity of a metal is given approximately by ɛ(ν) = 1 − ν*_e_*^2^/ν^2^). For two metals in vacuum, the Hamaker constant is [23]: $A≅\frac{3}{8\sqrt{2}}h\frac{\nu_{e1}\nu_{e2}}{\nu_{e1}+\nu_{e2}}≅4.10^{-19}\text{J}$, in which ν_e1_ and ν_e2_ are the plasma frequencies of the free electron gas, typically in the range (3–5) × 10^15^ s^−1^.

The equations above exhibit some important features:

- The vdW force between two identical bodies in a medium is always attractive (*A* is positive), whereas the force between two different bodies may be attractive or repulsive. If *ɛ*_3_ and *n*_3_ are intermediate between *ɛ*_1_ and *ɛ*_2_ and *n*_1_ and *n*_2_, respectively, *A* is negative (repulsive). Hamaker noted this [78], which was supported by Derjaguin [88], while Visser [89] established the precise conditions necessary for repulsive vdW-London forces. Fowkes [90] was the first to indicate a few possible examples of such repulsions, and van Oss *et al*. [91] demonstrated the existence of many such systems;
- The vdW force between any two condensed bodies in vacuum or in air (*ɛ*_3_ = 1 and *n*_3_ = 1) is always attractive (*A* is always positive);
- If *ɛ*_3_ and *n*_3_ equal the dielectric constant and index of refraction of either of the two bodies, *A* vanishes;
- The polar term cannot be larger than (3/4) *k_B_T*;
- Since *hν* >> *k_B_T*, as for interactions in free space, the dispersion force contribution (*ν* > 0) is usually greater than the dipolar contribution (*ν* = 0);
- The vdW force is much reduced in a solvent medium.

In other words, vdW forces can be attractive, repulsive or zero. The judicious choice of the medium in which an atomic force spectroscopy (AFS) experiment is carried out helps control the vdW forces between tip and sample. For non-conducting (non-metallic) solids and liquids interacting in vacuum or air (ɛ_3_ = *n*_3_ = 1) the Hamaker constant is typically in the range *A* = (5–10) × 10^−20^ J, while for interactions in a liquid medium such as water, the Hamaker constant is typically one order of magnitude smaller, in the range *A* = (0.5–1.5) × 10^−20^ J. For example, *A*~0.8 × 10^−20^ J is used for lipid bilayers [23], and is estimated as *A*~(1.0–1.5) × 10^−20^ J for proteins interacting in water or salt solutions, being slightly lower in high salt concentrations [92]. Hamaker constants for materials commonly used in AFS are listed in ref. [93]. Usual AFM tips are made of silicon nitride or silicon, one common substrate is mica and the colloidal probes are usually silica spheres.

**Figure 3:**
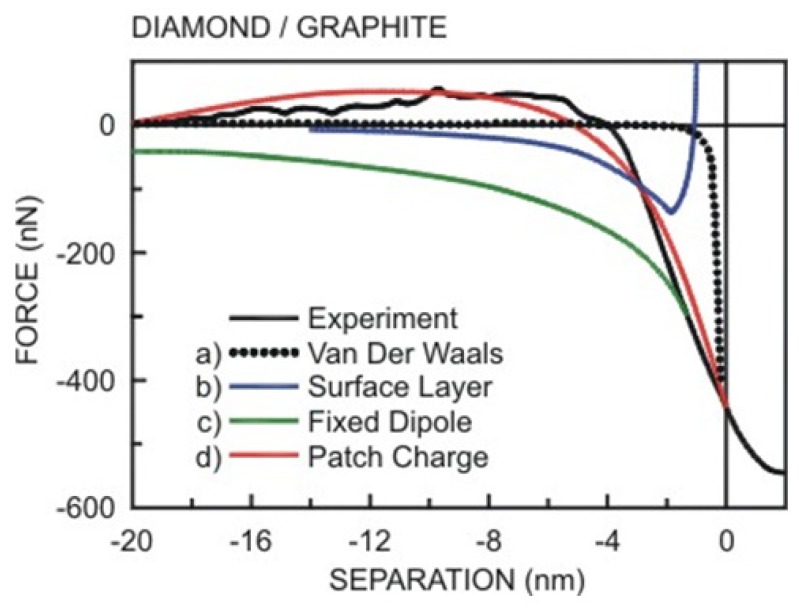
Attractive force curve for diamond-graphite (*k_c_* = 260 Nm^−1^, *R_t_* = 300 nm) with various theoretical fittings: (**a**) vdW; (**b**) surface layer of dielectric material; (**c**) fixed dipole; and (**d**) patch charge. The values used for the vdW interactions were *R_t_* = 300 nm and *A* = 2.5 × 10^−19^ J. The thickness of the surface layer was 1nm, with a tip radius of 30 μm, *A* = 2.5 × 10^−19^ J and Δ*A* = 0.2. For the fixed dipole curve, the thickness of the dipole layers was 1nm, the dipole moments = 1.4 Debye, the volume density of the dipoles was 3.0 × 10^28^ m^−3^ and the tip radius was 300 nm (Reproduced by permission of IOP Publishing Ltd. [94]).

In Figure 3, one example of an experimental attractive force curve for a diamond tip against a graphite surface is shown, together with an attempt of fitting the curve with a vdW force (curve A) using a reasonable value of *A* [94]. To obtain the best fit for a sphere on flat geometry, it is necessary to postulate an unreasonably large value of *AR* [95]. Even then, the fit is very poor, the prediction being too short-range. One suggestion is to consider more complex types of long-range interactions, which depend on a change in material properties of the near-surface region, giving increased attraction at small separations, as we will see later.

There are two possibilities to obtain the Hamaker constant: measuring the dielectric function for all frequencies or measuring the attraction force for a known geometry directly. To measure the dielectric function, use may be made of spectroscopic methods such as electron energy loss spectroscopy, absorption in the UV-vis. range, infrared and microwave spectroscopy [96]. A method to quantitatively evaluate the Hamaker constant using the jump-into-contact effect in AFM was developed by Das *et al*. [97]. They found that the jump-to-contact (see more details in Section 4) of the cantilever in the atomic force microscope is caused by an inherent instability in the motion of the AFM cantilever. The Hamaker constant was determined from the cantilever deflection at the jump-to-contact using the force constant of the cantilever and the tip radius of curvature, all of which can be obtained with AFM measurements. This method is applicable only to surfaces that have vdW interaction as the tip-sample interaction. Another interesting method consists in calculating the work of adhesion and then relating it to the Hamaker constant through [98]:

(3.11) $$\varpi =\frac{A}{\beta a_{o}^{n}}$$

Where β and *n* depend on the geometry of the system and can be calculated from the force laws listed in Table 1.

**Figure 4:**
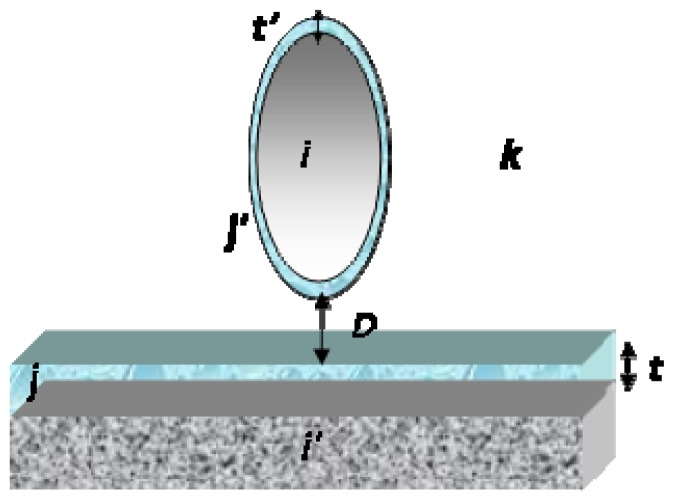
Scheme of two surfaces (*i* (tip) and *i*′ (sample)) interacting across of a medium, *k*, with adsorbed layers *j* and *j*′ of thickness *t* and *t*′.

The equations for surface interactions previously mentioned were derived for the situation in which the interacting units were separated by a vacuum. Special features appear when the measurements are performed under ambient conditions because both the tip and the sample surface may be coated with a thin water film. vdW interactions are affected by adsorbed layers of a dielectric material, with the Hamaker constant depending on the permittivity of the adsorbed layers. The effective Hamaker constant *A_iki_*_′_ for two surfaces *i* and *i*′ with adsorbed layers *j* and *j*′ of thickness *t* and *t*′, across medium *k* can be evaluated using [103] (see Figure 4):

(3.12) $$A_{{iki}^{\prime }}=A_{{jkj}^{\prime }}-A_{ikj}-A_{i^{\prime }kj}+A_{i^{\prime }ji}$$

where, for this case, *j* = *j*′ (water).

Burnham *et al*. [94] suggested an expression for an effective Hamaker constant, based on a simple situation where a dry gas *k* separates two identical solids *i* that are both coated with layer *j* with thickness *t*:

(3.13) $$A=A_{jkj}-\frac{2A_{ijk}}{1+{\left(t/D\right)}^{3}}+\frac{A_{iji}}{1+{\left(2t/D\right)}^{3}}$$

Thus, for reasonably small values of *t*/*D*, *A* = (*A_jkj_* − 2*A_ijk_* + *A_iji_*) + (*A_ijk_* − *A_iji_*)(6*t*/*D*).

On the basis of reasonable values of *t* and of the constant term in Equation 3.13, Burnham *et al*. [94] obtained the fitting shown in Figure 3 (curve (b)). Considering the surface layer (Equation 3.13) the best fitting was obtained.

The terms in Equation 3.12 can be found by combining relations, which is frequently done for obtaining approximate values for unknown Hamaker constants in terms of known ones. Considering *A_ikj_* from Equation 3.12 as the non-retarded Hamaker constant for media *i* and *j* interacting across medium *k*[23]:

(3.14) $$A_{ikj}\approx \left(\sqrt{A_{ii}}-\sqrt{A_{kk}}\right)\left(\sqrt{A_{jj}}-\sqrt{A_{kk}}\right)$$

where *A_ii_*, *A_kk_*, and *A_jj_* are the Hamaker constant of the AFM tip, the medium and water, respectively. As an illustration of the above relations let us consider a system comprising silicon (*i*), air (or vacuum) (*k*), and water (*j*), for which Equation 3.14 would predict: $A_{ikj}\approx \left(\sqrt{18.65}-\sqrt{0}\right)\left(\sqrt{3.7}-\sqrt{0}\right)\times 10^{-20}=8.3\times 10^{-20}\text{J}$. When two surfaces of component *i* are separated by a medium of component *k*, *i.e.*, *i = j*, the effective Hamaker constant is approximated by:

(3.15) $$A_{iki}\approx {\left(\sqrt{A_{ii}}-\sqrt{A_{kk}}\right)}^{2}$$

where *A_ii_* is the Hamaker constant for component *i* in a vacuum, and *A_kk_* is the corresponding constant for component *k*. As the Hamaker constants for *i* and *k* become similar, the effective Hamaker constant tends toward zero, and the free energy of attraction between the two surfaces is also reduced to zero. As discussed in Section 5, such a reduction in attractive forces due to an intervening medium provides one way to successfully prevent spontaneous joining of surfaces [2].

The Supplementary Material of this Review lists Hamaker constants for AFM studies, including values of a variety of conditions under which the force curves were measured, in some cases attaching colloidal spheres of different materials to AFM cantilevers. It should be stressed that Hamaker constants for many practical systems are still unknown, for they may be difficult to determine [104].

## 4. Introduction to Atomic Force Spectroscopy (AFS)

**Figure 5:**
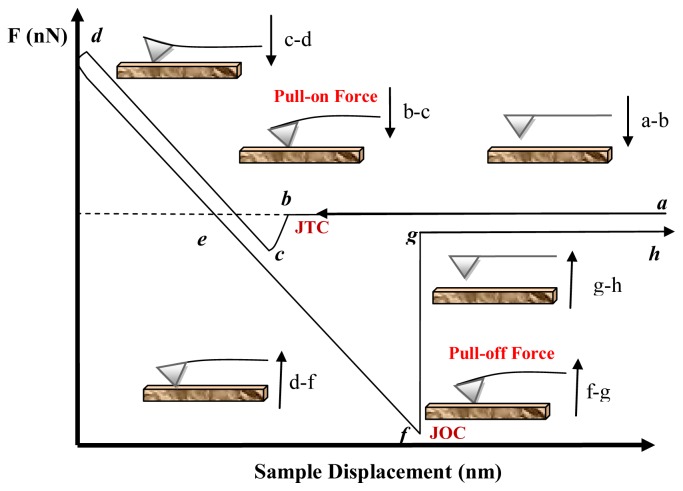
Force curve on a wood surface illustrating the points where jump-to-contact (JTC) (approach) and jump-off-contact (JOC) (withdrawal) occur and the maximum values of the attractive force (pull-on force and pull-off force).

AFM can be used to determine the dependence of the interaction on the probe-sample distance at a given location [105], in the so-called atomic force spectroscopy (AFS). AFS may be performed in two ways: local force spectroscopy (LFS) and force imaging spectroscopy (FIS). In LFS, the force curve is determined at a particular location on the sample surface, as shown schematically in Figure 5. Force curves are plots of the deflection of the cantilever (force) *versus* the extension of the piezoelectric scanner (sample displacement); if the cantilever spring constant is known, then the force can be calculated or measured. These curves can be used to measure the vertical force that the tip applies to the sample surface and to study the surface properties of the sample, including the elastic deformation of soft samples. They can also be used to monitor the unfolding of protein molecules as the latter are pulled from the sample surface by the AFM tip.

In the diagram of Figure 5 is shown a typical F *vs.* D curve obtained with a soft cantilever on a hard sample. Segment *a*–*d* represents the first half cycle (approach curve) while segment *d*–*h* is the second half cycle (withdrawal curve) of the curve. These cycles can be divided roughly into three regions: the contact line, the non-contact region and the zero line. The zero line is obtained when the tip is far from the sample and the cantilever deflection is close to zero. For measurements in a liquid, this line gives information on the viscosity of the liquid [106]. When the sample is pressed against the tip, the corresponding cantilever deflection plot is referred to as the contact line, which can provide information on sample stiffness. The most interesting regions of the force curve are two non-contact regions, containing the jump-to-contact (JTC) and the jump-off-contact (JOC). The non-contact region in the approach curve provides information about attractive (vdW or Coulomb force) or repulsive forces (vdW in some liquids, double-layer, hydration and steric force) before contact; this discontinuity occurs when the gradient of the tip-sample force exceeds the spring constant of the cantilever. The maximum forward deflection of the cantilever multiplied by the effective spring constant of the cantilever is the *pull-on force* [94]. The non-contact region in the withdrawal curve contains the jump-off-contact, a discontinuity that occurs when the cantilever’s spring constant is greater than the gradient of the tip-sample adhesion forces. The maximum backward deflection of the cantilever multiplied by the effective spring constant of the cantilever is the pull-off force [94].

At the start of the cycle (point *a*) a large distance separates the tip and the sample, there is no interaction and the cantilever remains in a non-interacting equilibrium state. As separation decreases, in *a–b* the tip is brought into contact with the sample at a constant speed until it reaches a point close to the sample surface (point *b*). Once the total force gradient acting on the tip exceeds the stiffness of the cantilever, the tip jumps to contact (JTC) with the sample surface (*b–c*). JTC is often due to capillary forces from the moisture layer that covers the tip and the sample surface. In (*c–d*), the tip and sample are in contact and deflections are dominated by mutual electronic repulsions between overlapping molecular orbitals of the tip and sample atoms. The shape of segment (*c–d*) indicates whether the sample is deforming in response to the force from the cantilever. The slope of the curve in the contact region is a function of the elastic modulus and geometries of the tip and sample [29].

Segment (*d–e*) represents the opposite movement to segment (*c–d*), with the tip being withdrawn. If both segments are straight and parallel to each other, there is no additional information content. If they are not parallel, the hysteresis gives information on plastic deformation of the sample [26,29]. In segment (*d–f*) the sample is being retracted and adhesion or bonds formed during contact with the surface cause the tip to adhere to the sample. As the sample continues retracting, the spring force of the cantilever overcomes the adhesion forces and the cantilever pulls off sharply (*f–g*). In this segment, several long and short-ranged forces become effective (see Table 2) [43,107,108]. The force at point *f* is the total adhesive force between the tip and the sample. In segment (*g–h*) the cantilever is moved upwards to its undeflected or noncontact position. The adhesive force can be measured through deflections of a spring, according to Hooke’s law:

(4.1) $$F=-k_{c}\delta_{c}$$

where the cantilever deflection δ*_c_* is determined by the acting force *F* and the spring constant of the cantilever, *k_c_*. Although cantilevers have a spring constant defined by the manufacturer, it is important to calibrate the system, as there have been cases where the actual spring constant was one order of magnitude off the nominal value. A number of methods for determining the spring constant have been proposed [109–112]. In the calibration, one has to measure the resonant frequency of the cantilever before and after addition of a small mass onto the tip [109]. Moreover, one has to determine the unloaded resonant frequency using the cantilever’s density and dimensions [110,113], or through thermal fluctuations of the cantilever [114,115].

In summary, using AFS makes it possible to obtain the following information: (*i*) the magnitude of the long-range attractive and adhesive forces [29,81]; (*ii*) estimation of the point of tip-sample contact; (*iii*) the tip-sample contact area; and (*iv*) the elastic modulus and plasticity of thin and thick films [116,117].

**Figure 6:**
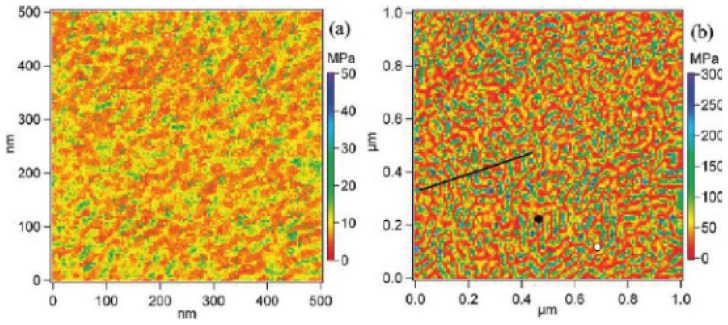
Force imaging spectroscopy—Young’s modulus maps of (a) SEBS (10/80/10) and (b) SEBS (21/58/21) (Reprinted with permission from [119] (© 2010, American Chemical Society)).

In *force imaging spectroscopy* (FIS), force curves are recorded at a large number of sample surface locations [118]. Figure 6 shows Young’s modulus maps obtained from nanomechanical mapping measurements for poly(styrene-b-ethylene-co-butylene-b-styrene) (SEBS) samples having different compositions [119]. The characteristic phase-separated morphologies consisting of high and low Young’s modulus regions are clearly exhibited. The contrasts in the maps reflect the variations in Z-travel needed to damp the amplitude of the interacting probe to a trigger level chosen by an operator. In the Young’s modulus maps, the light green areas with higher Young’s moduli are considered to be hard PS blocks, while the red areas with lower Young’s moduli are considered to be soft PEB blocks. Such experiments allow precise identification of surface locations occupied by different blocks and offer experimental data for nanomechanical models for extracting quantitative data.

**Figure 7:**
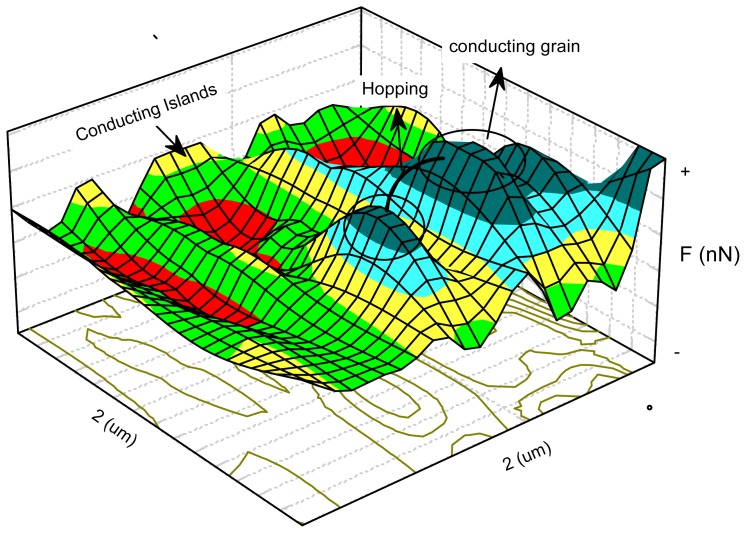
Map of forces obtained with AFS showing regions of repulsive (conducting islands) and attractive interactions on POEA films in solution (pH = 3).

The FIS mode can be used to measure adhesion [120], hardness, or deformability of samples and vdW interactions. Maps of interaction can be produced also by measuring the vertical displacement of the sample–driven by the piezoscanner–and the deflection of the cantilever with respect to its position at rest on several points of the surface. Force curves are digitally acquired at 100 or more points equally spaced from each other over the sample surface scanned area. By way of illustration, Figure 7 shows an adhesion map with islands of repulsive forces with diameter varying from 100 to 470 Å (average size = 306 ± 109 Å) in a matrix with attractive forces. These islands are made of protonated, semi-crystalline PANI. The result presented by AFS is in surprisingly good agreement with the value (200 to 300 Å) estimated by Zuo *et al.* [121] based on *ac* conductivity measurements. These charged domains provided new evidence for the formation of conducting islands [121–123].

Leite *et al.* [81] measured the variability in adhesion due to contamination and surface roughness using adhesion maps from the distribution of the measured forces. In addition to identifying regions contaminated by either organic compounds or adsorbed water, it was possible to estimate the adhesion force in air and water. The experimental results were in good agreement with theoretical calculations, where the adhesion forces in air and water were mostly associated with capillary and vdW forces, respectively. A small long-range repulsive force was observed in water due to the overlapping electrical double-layers formed on both the tip and sample surfaces.

Tapping mode AFM (Intermittent Contact-AFM) has also been used to map tip-surface interactions [124,125]. In this mode, the cantilever is oscillated at its resonant frequency at a position just above the surface, so that the tip is only in contact with the surface for a very short time. A feedback loop ensures that the amplitude of the cantilever oscillation remains almost constant. It is possible to measure the phase difference between the driving oscillation and the detected cantilever oscillation, generating a phase difference map. An increase in the phase difference arises from a stronger tip-sample interaction, creating contrast in the phase map [126]. There are still, however, problems associated with the methods for determining tip-sample interactions, and the nature of interactions leading to image contrast is under debate [127]. Kitamura and Iwatsuki [128] analyzed the use of noncontact atomic force microscopy to detect variations in surface composition, *i.e.*, to detect a ‘spectroscopic image’ of the sample. The authors showed that long-range forces depend on the composition of the AFM tip and of the sample. They demonstrated how vdW forces may be utilized for AFM spectroscopy. vdW interactions have been detected for samples under high vacuum conditions [129–131].

## 5. Measuring and Calculating van der Waals and Adhesion Forces

The interactions between two surfaces depend on whether the system is in vacuum, in air or ambient conditions or in a liquid. In vacuum, there are long-range vdW and electrostatic (Coulombic) forces, while at smaller surface separations–corresponding to molecular contact (D ~ 0.1–0.2 nm)—there are additional forces such as covalent, hydrogen and metallic bonding forces. All of these forces determine the adhesion between bodies of different geometries, the surface and interfacial energies of planar surfaces, and the strengths of materials, grain boundaries, cracks, and other adhesive junctions [21]. When exposed to a vapor, e.g., atmospheric air, two solid surfaces in or close to contact may have a surface layer of chemisorbed or physisorbed molecules, or a capillary condensed liquid bridge between them [132,133]. Each of these effects can drastically modify adhesion. The adhesion force usually decreases, but in the case of capillary condensation, the additional Laplace pressure between the surfaces may cause the adhesion to be stronger than in an inert gas or vacuum. The force between two surfaces totally immersed in a liquid is again different from that in vacuum or air. The vdW attraction is generally reduced, but other forces come into play, which can qualitatively change both the range and even the sign of the interaction, as discussed in Section 3. The attractive forces depend on the surfaces characteristics, being stronger for two hydrophobic surfaces interacting in water and weaker for two hydrophilic surfaces. In addition, the force may no longer be purely attractive; it can be repulsive, or the force can change sign at some finite surface separation.

Because the factors mentioned above are important for determining the strength of vdW interactions and adhesion in different systems, we shall consider them in separate subsections, also distinguishing between measurements of pull-on (approach curve) or pull-off forces (withdrawal curve).

### 5.1. Interactions in Vacuum

#### 5.1.1. Attractive Forces (pull-on forces)

The attractive forces or pull-on forces, *F_pull-on_*, in vacuum, can comprise two components: the non-electrostatic, *F_ne_*, and the electrostatic forces, *F_e_*:

(5.1) $$F_{pull-on}=F_{ne}+F_{e}$$

where *F_ne_* comprise the vdW forces (*F_vdW_*). Thus, the pull-on force in vacuum is given by:

(5.2) $$F_{pull-on}^{vac}=F_{vdW}+F_{e}$$

##### 5.1.1.1. Electrostatic Forces (*F_e_*)

The electrostatic forces come basically from the effect of electric fields on electrical charges [134]. The measurement of these Coulomb forces can be useful to investigate the tip shape and its influence on surface roughness and mechanical deformation at contact [29,134,135]. To describe these concepts, several analytical models exist in the literature, which are based on three assumptions [134]:

1. Surfaces are smooth, and the surface topography is not taken into account;
2. The materials are conductive with charge uniformly distributed, only on the surface, and the electric field is normal to the surface;
3. There is no charge between contacting objects.

###### 5.1.1.1.1. Plane–Plane Model

This model gives the electrostatic pressure and requires the electrostatic force given by [134,136]:

(5.3) $${\text{F}}_{\text{plane}}=\frac{{ɛ}_{0}{\text{V}}^{2}\text{A}}{2{\text{z}}^{2}}$$

where ε_0_ is the free space permittivity, V is the potential difference, A is the area of contact and *z* is the separation distance (gap). Additionally, very small distances between objects are used when the contact is estimated for flat surfaces.

###### 5.1.1.1.2. Sphere–Plane Model

The sphere models have been developed for more complex shapes and larger separation distances. These models give an approximation of the electrostatic forces for the contact between a conductive sphere and a conductive plane, thus simulating the case of an AFM tip and the sample. For all separation distances a general expression has been developed [98,134,137]:

(5.4) $$F_{sphere}=\pi {ɛ}_{0}\frac{R^{2}V^{2}}{z(z+R)},\text{for }R\ll z\ll L$$

where ε_0_ is the free space permittivity, *R* is sphere radius (m), *V* is the potential difference, *L* is the length of the tip (m) and *z* is the separation distance (gap).

###### 5.1.1.1.3. Uniformly Charged Line Models (Conical Tip Models)

The principle consists in replacing the equipotential conducting surfaces by the equivalent image charges. The main hypothesis is that the cone may be approximated by a charged line of constant charge density λ_0_ given by Hao *et al.* [138] for a small aperture angle (θ≤π/9) as:

(5.5) $$\lambda_{0}=4\pi {ɛ}_{0}V{\left[\text{ln}\left(\frac{1+\text{cos }\theta }{1-\text{cos }\theta }\right)\right]}^{-1}$$

The resulting force is [93]:

(5.6) $$F_{C}≅\frac{{\lambda_{0}}^{2}}{4\pi {ɛ}_{0}}\text{ln}\left(\frac{L}{4z}\right)$$

in which *L* ≤ *z* ≤ *R*. This model fits well the experimentally measured forces at large tip-sample separations.

###### 5.1.1.1.4. The Asymptotic Model

The principle is to decompose a conical tip into infinitesimal surfaces [137]. The contribution of the apex and the spherical tip are evaluated separately and then added to get the total force, which is given by:

(5.7) $$F_{asymp}=\pi {ɛ}_{0}V^{2}\;\left[\frac{R^{2}(1-\text{sin}\theta )}{z[z+R(1-\text{sin}\theta )]}+k^{2}\;\left(\text{ln}\frac{L}{z+R(1-\text{sin}\theta )}-1+\frac{R\;{\text{cos}}^{2}\theta /\text{sin}\theta }{z+R(1-\text{sin}\theta )}\right)\right]$$

where $k^{2}=\frac{1}{{\left\{\text{ln}\left[\text{tan}\left(\frac{\theta }{2}\right)\right]\right\}}^{2}}$.

###### 5.1.1.1.5. The Hyperboloid Model (Hyperboloid Tip Model)

In this model the tip is represented by hyperboles bounded by a maximum distance *r*_max_ from the axis. The expression is derived by solving the Laplace equation in a prolate spheroidal coordinate system and by treating the tip-sample geometry as two confocal hyperboloids [139,140].

(5.8) $$F_{hyp}=4\pi {ɛ}_{0}V^{2}\frac{\text{ln}\;\left[1+{\left(\frac{r_{max}}{R}\right)}^{2}\;\left(1+\frac{R}{z}\right)\right]}{ln^{2}\;\left(\frac{1+\eta_{tip}}{1-\eta_{tip}}\right)}$$

in which $\eta_{tip}=\sqrt{z/z+R}$ and *r*_max_ is the cut-off radius introduced to avoid divergence.

###### 5.1.1.1.6. The Cylindrical Model

This model differs from the previous one for being two-dimensional and not axisymmetrical. Using the analytical model for the cylinder–plane contact described by Smythe [141], the electrostatic force is given by [134]:

(5.9) $$F_{electrostatic}^{nondeformed}(N/m)=\frac{\pi {ɛ}_{0}{ɛ}_{R}\sqrt{RV^{2}}}{2\sqrt{2z\;3/2}}=\frac{\pi {ɛ}_{0}{ɛ}_{R}\lambda V^{2}}{4\sqrt{2\pi }\sqrt{Az\;3/2}}$$

where ε_0_ and ε*_R_* are the permittivity of free space and the relative permittivity of the environment, respectively and λ is the wavelength.

##### 5.1.1.2. van der Waals Forces (*F_vdw_*)

The first attempts to measure vdW interactions began in the 1950’s with [88] and [142] for optically flat, polished glass plates in a vacuum. Experiments were performed with separations, *D*, between 500–2000 nm [142] and 500–950 nm [143]. Measurements between a sphere and a plate were made for separations of 100–700 nm [99] and 94–500 nm [143]. The separations between surfaces used in these experiments are beyond the range over which non-retarded vdW interactions dominate, usually of the order of 10 nm [78]. Thus, the values obtained are for retarded interactions, or in the transition regime between the two. The measurement of non-retarded vdW interactions required substantial improvements in sample smoothness and control of separations, obtained only with sophisticated equipment.

The first inter-surface force measurement at separations of the order of 1–10 nm was conducted by [47], using crossed cylindrical surfaces of cleaved mica, separated by air. A piezoelectric crystal was used to control the separation precisely. This mechanism was adapted to measure the surface forces between crossed mica cylinders in aqueous solutions [22], now named the surface force apparatus (SFA). The forces between mica surfaces were measured from 1–100 nn in aqueous KNO_3_ solutions at pH 6. With AFM force curves have been obtained for various materials separated by vacuum or liquids [98,144–146].

An analytical expression for the force curve (pull-on force) may employ the derivation of Hao *et al*. [138]. The cantilever-sample system can be described by means of a potential *w_tot_*, which is the sum of three potentials: *w*_tot_ = *w_cs_*(*D*) + *w_c_*(δ*_c_*) + *w_s_*(δ*_s_*). Here *w_cs_*(*D*) is the interaction potential between the tip and sample (*D* is the sample-tip distance), e.g., the Lennard-Jones potential, *w_c_*(δ*_c_*) is Hooke’s elastic potential of the cantilever (*w_c_*(δ*_c_*) = ½ *k_c_*δ*_c_*^2^) and *w_s_*(δ*_s_*) is the potential that describes the sample deformation according to Hooke’s law (*w_s_*(δ*_s_*) = ½ *k_s_*δ*_s_* ^2^), where *k_c_*, *k_s_*, δ*_c_*, and δ*_s_* are the cantilever and sample spring constants, the deflection of the cantilever and deformation of the sample, respectively.

**Figure 8:**
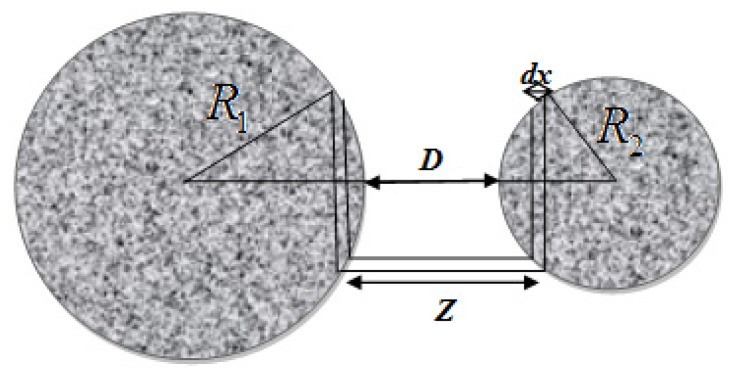
Geometry employed in the Derjaguin approximation (sphere-sphere), *z* is the distance between the circular sections, *D* is the distance between the two bodies (spheres), *R* is the radial coordinate.

The Lennard-Jones (L-J) potential is composed of two interactions: the vdW attraction and the Pauli repulsion [147,148], which is the repulsion caused by the overlap of the electron clouds of two atoms. The quantum mechanical calculations for the resulting potential of the overlap of the wave functions yield an exponential dependence [149], which is normally approximated by a power law with *n* > 9. For the Lennard-Jones potential *n* = 12 is chosen. At relatively large separations, typically of the order of 1 nm or more, vdW interactions lead to a negative interaction potential and thus to attractive forces, which are present in any environment. For a pair potential in the form *w*(*D*) = −*G*/*D^n^*, where *G* is the constant in the atom-atom pair potential, and assuming that the potential is additive, the interaction energies between macroscopic bodies may be obtained *via* integration. In the case of two interacting spheres at distance *D*, the force *F*(*D*) can be obtained by integrating over small circular sections of surface 2π*x*d*x* on both spheres [150], as depicted in Figure 8:

(5.10) $$F(D)=\int_{D}^{\infty }2\pi x\text{d}xf(z)$$

in which *f*(z) is the normal force per unit area. Thus, the total force between the sphere and the plate is:

(5.11) $$F(D)=2\pi \frac{R_{1}R_{2}}{R_{1}+R_{2}}w{\left(D\right)}_{planes}$$

where *w*(*D*)_planes_ is the energy per unit area between two identical flat plates (surfaces), and *R_1_* and *R_2_* are the radii of the two spheres interacting. This relationship shows that the force between a sphere and a plate is directly proportional to the energy per area between identical flat plates. Equation 5.11 is known as the Derjaguin approximation [150,151], which applies when *R* >> *D* [152], *i.e*., whenever the interaction range and the separation *D* are much smaller than *R*_1_ and *R*_2_. From the Derjaguin approximation, with *R_2_* >> *R_1_*, one obtains the force between a sphere and a flat surface:

(5.12) $$F(D)=2\pi R_{t}w{\left(D\right)}_{planes}$$

where *R_t_* is the radius of the AFM tip.

vdW energies between macroscopic bodies in vacuum may be computed *via* integration only in the approximation that the forces are assumed additive and non-retarded. The interaction laws obtained *via* integration are listed in Table 3 for the most common geometries. For extended electrically neutral bodies, e.g., a sphere above a flat plane, a proper accounting of geometry must be included, resulting in an interacting force that varies with the sphere-substrate separation as *D*^−2^.

**Figure 9:**
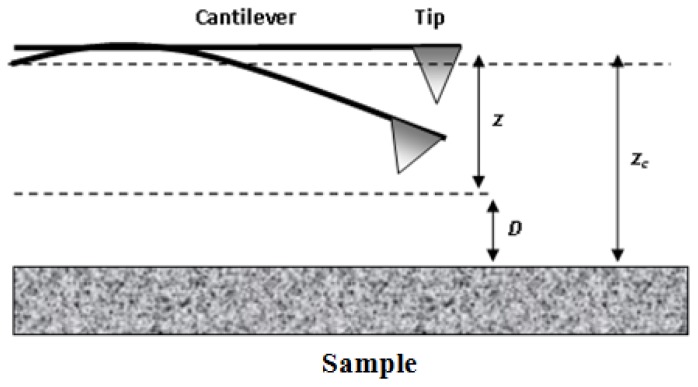
Scheme of the relevant spatial distances in AFM. *D* is the tip-sample distance, whereas *z_c_* is the distance between the sample and the cantilever rest position, and *z* is the cantilever deflection.

Using the form of the vdW potential for two flat surfaces (Derjaguin’s equation), the vdW force between a sphere and a flat surface in vacuum is [153]:

(5.13) $$F{\left(z_{C}-z\right)}_{sphere-plane}=-\frac{AR_{t}}{6D^{2}}$$

where *z_c_* is the height from the bottom to the tip above the surface in the absence of external forces, *z* or δ*_c_* is the deflection of the cantilever due to short- and long-ranged forces acting on the tip and *D* is the tip-surface separation, *D* = *z_c_* − *z* (see Figure 9).

The experimental determination of the tip-surface separation (*D*) (see Table 1), often-named jump-to-contact distance (*D_jtc_*), is not straightforward, requiring a more accurate quantitative analysis. When AFM is operated in vacuum and the tip and sample are electrically neutral, the tip will be subjected to three forces: a spring force due to the cantilever, a short-ranged repulsive contact force, and a long-ranged attractive vdW force. In the absence of short-ranged repulsion, the tip position at equilibrium will be such as to balance the vdW and spring forces. This point may be determined by finding the energy minimum for the system. Additionally, to convert the diode-voltage versus sample-displacement data to a force *vs*. tip-sample separation curve, it is necessary to define zeros of both force and separation and to convert the diode signal to cantilever deflection and force. The zero of force can be chosen when the deflection is constant (where the tip and sample surface are far apart), and the zero of distance can be chosen when the cantilever deflection was linear with respect to the sample at large forces [144]. In the experiments, the relationship between sample displacement and diode response in the region of constant compliance is independent of the surface force and is used to convert the diode response into the deflection of the cantilever. This conversion is then used to determine changes in tip-surface separation, with the relative surface separation being calculated by adding the displacement of the sample to the deflection of the cantilever.

The potential energy may be written as [154]:

(5.14) $$w(z,z_{c})=-\frac{AR_{t}}{6D}+\frac{1}{2}k_{c}z^{2}$$

Local minima of the energy function are found by setting its derivative to zero:

(5.15) $$\frac{dw(z,z_{c})}{dz}=\frac{AR_{t}}{6D^{2}}+k_{c}z=0$$

Thus, the length scale at which the vdW forces are able to deflect the spring significantly is given by $\beta \equiv \sqrt[{3}]{\frac{AR_{t}}{k_{c}}}$. The jump-to-contact distance, *D_jtc_*, is specified as $D_{jtc}\equiv \frac{\beta }{\sqrt[{3}]{3}}$ [154], and:

(5.16) $$D_{jtc}\equiv \frac{\beta }{\sqrt[{3}]{3}}=\sqrt[{3}]{\frac{AR_{t}}{3k_{c}}}$$

For typical values of the Hamaker constant, radius tip and spring constant (10^−19^ J, 50 nm, and 0.1 N/m, respectively), the tip will jump into contact with the surface when it is 26 Å apart [154].

Butt *et al.* [98] considered the sample stiffness, *k*_s_, in which case the potential energy described in Equation 5.15 can be written as:

(5.17) $$w(z,z_{c})=-\frac{AR_{t}}{6D}+\frac{1}{2}k_{c}z^{2}+\frac{1}{2}k_{s}{\delta_{s}}^{2}$$

where δ_s_ is the deformation of the sample. For a sphere-plane system, it can be shown that the distance at *D*_jtc_ is given by:

(5.18) $$D_{jtc}=\sqrt[{3}]{\frac{AR}{3k_{eff}}}$$

where $k_{eff}=\frac{k_{c}k_{s}}{k_{c}+k_{s}}$.

Equation 5.18 allows one to calculate *A*, *R* (*k*_eff_) and *D*_jtc_ once the elastic constant of the cantilever *k*_c_ and the effective elastic constant of the system *k*_eff_ are known. Equation 5.18 differs from Equation 5.16 due to the inclusion of the sample elastic constant, *k_s_*. For separations *D_jtc_* < *r*_0_, where *r*_0_ is an intermolecular distance introduced to avoid the divergence of Equation 5.9, often given by *r*_0_ = 0.165 nm [23], the resulting vdW force is identified with the adhesion force derived from one of the models presented in the previous section.

Several experiments have demonstrated the capability of AFM in probing vdW forces with high resolution in distance and force [29,93,98] with measurements in vacuum or in dry N_2_ for systems such as silicon tungsten/graphite or tungsten/gold [116], tungsten/mica or tungsten/alumina [155] and Ni/Au [156]. Burnham *et al.* [155] measured forces between a tungsten tip and several samples under dry nitrogen, as shown in Table 4. They concluded that the attraction force with AFM depends on the sample surface energy and contact area, which was normalized for the tip radius by dividing by 4π*R*_t_. Goodman and Garcia [157] estimated vdW forces in vacuum as being of the order of 10–20 nN, whereas these forces between metallic tips and samples were as high as 100 nN when the AFM was operated in the purely repulsive mode.

The vdW interactions (energy and force) between a spherical particle and an infinite cylinder were derived by [158] using the method of additive summation of the pair interactions described by the potential of the general form $w(r)=-\frac{C_{vdW}}{r^{m}}$, where *C_vdW_* is the interaction constant. The authors also derived a compact formula for the energy of vdW interactions of a point-like particle (atom, molecule) with a sphere and a cylinder for the case of arbitrary *m*. This study is important since many problems in physics, chemistry, and biology deal with the vdW interaction of fine spherical particles with bodies of cylindrical shape, like nanowires, nanotubes, and fibers. The non-retarded vdW force between a sphere (*s*) and a cylinder (*c*) is [158]:

(5.19) $$F^{sc}=-\frac{A_{6}R_{s}^{3}}{24c^{\frac{2}{5}}{R_{c}}^{\frac{3}{2}}s^{2}p^{\frac{3}{2}}}\left\{\left(4+2s+s^{2}\right)w\left(\frac{1}{p}\right)-s(1+s)K\left(\frac{1}{p}\right)\right\},$$

where *R* *_s_* and *R* *_c_* are the radii of the sphere and cylinder, respectively, *D* is the distance between both, *c = D + R_s_* *+ R_c_, p =* 1+*s*/2, *s* = ((*D+R_s_*)^2^−*R_s_*^2^)/2*cR_c_*. *K* and *w* are the complete elliptic integrals defined as [159]:

(5.20) $$w(z)=\int_{0}^{\pi /2}\sqrt{1-z\;{\text{sin}}^{2}\;\theta \text{d}\theta }$$

(5.21) $$K(z)=\int_{0}^{\pi /2}1/\sqrt{1-z\;{\text{sin}}^{2}\;\theta \text{d}\theta }$$

For distances larger than 20 nm, retardation effects (Casimir Effects) become dominant. Casimir and Polder [160] showed that the interaction energy between two atoms is approximately described by −*C*_3_/*r*^7^ rather than −*C*_1_/*r*^6^, *i.e.*, if the time required for light to travel from atom 1 to interacting atom 2 is comparable to the inverse of the frequency of fluctuations dipoles, attraction is reduced. Therefore, at distances *r* ≥ λ_0_, where λ_0_ is the characteristic wavelength of radiation in the spectra of interacting atoms given by λ_0_ = 2π*c*ϖ_0_^−1^ [158] (ϖ_0_ is the atomic frequency, *c* is the light velocity in vacuum), the dispersion interaction is no longer instantaneous. It is determined by the finite time of the signal propagation from one dipole to another, 2*rc*^−1^, which results in the retardation of the vdW interaction. In the limit r >> λ_0_ the London forces do not exist. In this case, the vdW interaction is fully retarded and reduced. The full expression for the Casimir and Polder potential, valid for all separations *r* >> *a*, is given by a cumbersome integral for which it is convenient to use a simple analytical approximation.

In the case of AFM systems, the retarded vdW force between a sphere and cylinder is:

(5.22) $$F(D)=2\pi R_{s}\frac{\sqrt{R_{c}}}{\sqrt{R_{s}+R_{c}}}\left(-\frac{1}{10\pi }\frac{A^{7}}{D^{3}}\right)$$

Theoretical issues of vdW forces in connection with force microscopy are discussed in detail in Hartmann [161]. Wennerstrom *et al.* [162] have also discussed the origins and effects of retardation on interactions between bodies.

#### 5.1.2. Adhesion Forces (Pull-Off Forces)

The concept of adhesion force or pull-off forces, a material property reflecting the influence from elastic deformation, surface roughness [163–165], and interfacial surface energy, is an efficient, quantitative measure of the adhering tendency of a powder or surface. The adhesion forces, *F_adh_* or *F_pull–off_*, in vacuum, can comprise two components: the non-electrostatic, *F_ne_* and the electrostatic forces, *F_e_* [166]:

(5.23) $$F_{pull-off}\;=F_{ne}+F_{e}$$

where *F_ne_* contains the vdW forces (*F_vdW_*) or vdW adhesiveness and the chemical bonding force (*F_chem_*). Thus, the total adhesion force or pull-off force in vacuum is given by:

(5.24) $$F_{pull-off}^{vac}\;=F_{vdW}+F_{chem}+F_{e}$$

##### 5.1.2.1. Electrostatic Forces (*F_e_*)

Owing to local inhomogeneities in the work function of a material, a microscopic charge transfer takes place, resulting in a dipole. If the work function of two patches varied by a total Δφ, a particle would have a dipole moment *p* = 4πɛ*R*^2^Δφ. If the cores of two particles are separated by *r*, the force on each particle will have a maximum value of [167]:

(5.25) $$F_{max}=\frac{3p^{2}}{\pi {ɛ}_{0}r^{4}}=48\pi {ɛ}_{0}{\left(\frac{R}{r}\right)}^{4}\;{\left(\Delta \varphi \right)}^{2}$$

and will vary with the orientation of the dipoles. This model is referred to as the patch charge model.

The interactions amid particles and surfaces become more intricate than simply distinguishing between patch charges and vdW interactions, since real particles are not perfect spheres and usually have an electric charge. For illustration, a 10 μm xerographic toner particle may have a typical charge of the order of 8fC [168]. If these toner particles were spherical and the charge uniformly distributed over their surface, the Coulombic forces would be small compared to the vdW interactions for the particles in contact with the surface. Therefore, the Coulombic attraction between these particles in contact with a grounded metal plate would be given simply by [167]:

(5.26) $$F_{I}=\alpha \frac{q^{2}}{16\pi {ɛ}_{0}R^{2}}$$

where α is a factor to correct for the polarization of the particle, *q* and *R* are the charge and radius of the particle, respectively and ɛ_0_ is the permittivity of free space. For a material with a dielectric constant (*k*) of 4, α = 1.9. For the ideal particle under consideration, *F_I_*= 10 nN [167].

Particles can acquire charge in several ways, with the most common source being charge buildup in powder handling due to triboelectric charge transfer. Tribocharging is expected to result in non-uniform charge distributions over particle surfaces, and this nonuniformity can play a significant role in adhesive effects [169]. Hays [168] proposed that the total charged area *A_t_* on a tribo-electrically charged toner particle represents a small part of the total toner particle’s surface area. Based on Hays’ model, the total charge would be *Q =* σ*A_t_*, where σ is the surface charge density. A small fraction, like 20%, of the charged surface area, *A_c_*, might be in close proximity to the conducting surface. If *A_c_* is much larger than the average distance between the charged surface and the conducting substrate, the magnitude of the electrostatic forces of adhesion can be expressed as $F_{E}=-\frac{a^{2}A_{c}}{2{ɛ}_{0}}$, and the total adhesion can be written as,

(5.27) $$F_{A}=-\frac{a^{2}A_{c}}{2{ɛ}_{0}}-W_{A_{c}}$$

where *W_A_c__* represents a non-electrostatic (*i.e*., surface-energy based) adhesion contribution. Estimates in the literature indicate that contact charging can produce surface charge densities ranging from 0.5 to 5 mC/m^2^ depending on the materials involved [170].

##### 5.1.2.2. Van der Waals Adhesiveness (*F_vdW_* )

The non-electrostatic component (*F_ne_*) can be understood as the sum of vdW and capillary forces. In vacuum, capillary forces are neglected. Thus, if the measurement is made in a ‘dry’ atmosphere, such as nitrogen or vacuum, the adhesion force, *F_adh_*, is due mainly to dispersion forces and may be explained with adhesion mechanics. For deformable bodies, the vdW adhesion force between two fine smooth spheres displaying ideal Hertzian elastic behavior was solved in the 1970s. Much of the present understanding of the elastic adhesion mechanics (adhesion and deformation) of spheres on planar substrates is based on the theoretical work of Johnson, Kendall and Roberts (JKR) (1971) [171] and of Derjaguin, Muller and Toporov (DMT) (1975) [172]. For a heterogeneous sphere/flat system, within the Derjaguin approximation one can write:

(5.28) $$F_{adh}^{DMT}=2\pi R\varpi_{ikj}$$

where ϖ_ij_ is the work of adhesion between two surfaces *i* and *j* in a medium *k*. The concept of measuring the strength of adhesion in terms of the work of adhesion, ϖ*_ikj_*, was first introduced by Harkins in 1928 [173]. From the JKR theory, separation will occur when the contact area between the surfaces is *a_adh_* *=* 63*a*_0_, where *a*_0_ is the contact area at zero applied load. This separation occurs for a pull-off force of:

(5.29) $$F_{adh}^{JKR}=-\frac{3}{2}\pi R\varpi_{ikj}$$

The JKR model should describe appropriately adhesion for larger spheres with high surface energies and low Young’s moduli, while the DMT model should be appropriate for describing adhesion of smaller spheres of low surface energies and higher Young’s moduli.

If there is plastic or elasto-plastic deformation neither the DMT nor JKR models hold. Instead, the Maugis and Pollock (M–P) analysis [174] can be used, at least for full plasticity. The MP analysis gives the pull-off force as [146]:

(5.30) $$F_{adh}^{MP}=\frac{3\pi \varpi_{ikj}K}{2{\left(\pi H\right)}^{3/2}}p^{1/2}$$

where *H* is the hardness of the yielding material and *K* is the effective elastic constant of the system given by: $K=\frac{4}{3}{\left(\frac{1-\nu_{s}^{2}}{E_{s}}+\frac{1-\nu_{t}^{2}}{E_{t}}\right)}^{-1}$ (*ν_s_, E_s_, ν_t_,* and *Et,* are the Poisson’s ratio and the Young’s moduli of surface and tip, respectively). The reduced Young’s modulus is $E_{o}=\frac{1}{K}$.

For ideally smooth surfaces the theoretically predicted *F^DMT^_adh_* and *F* *^JKR^_adh_* represent the lower and the upper limits of the experimentally measured *F_adh_*, respectively. Hence, one can write [175]:

(5.31) $$F_{adh}=-\alpha_{adh}R\varpi_{ikj}$$

where *α_adh_* (adhesiveness parameter) lies between $\left(\frac{3}{2}\right)\pi$ (for soft surfaces) and 2π (for hard surfaces). The two models differ substantially in predicted contact area, adhesion force and surface profile. JKR theory predicts a finite radius of contact under zero load and when surfaces separate: $a_{o(JKR)}={\left(\frac{6\pi \varpi_{ikj}R^{2}}{K}\right)}^{1/3}$ and $a_{s(JKR)}=\frac{a_{o}}{4^{1/3}}\approx 0.63a_{o}$, respectively. One can estimate the number of molecular contacts in adhesive interactions by dividing the contact area at pull-off, *a_s_*_(_*_JKR_*_)_, by the area occupied by a single functional group [176]. Corresponding quantities for DMT theory are $a_{o(DMT)}={\left(\frac{2\pi \varpi_{ikj}R^{2}}{K}\right)}^{1/3}$ and *a_s(DMT)_* = 0. The estimate of the number of molecular contacts in the DMT model must consider the range of intermolecular of forces *z_o_* (equilibrium size of the atoms at contact).

A self-consistent approach to the contact problem typically requires numerical solutions. Such calculations based on the Lennard-Jones potential showed that the DMT and JKR results correspond to the opposite ends of a spectrum of a non-dimensional parameter (so-called Tabor elasticity parameter) [177]: $\mu_{T}=2.92{\left(\frac{\varpi^{2}R}{K^{2}z_{0}^{3}}\right)}^{1/3}$.

Tabor suggested that when μ exceeds unity, the JKR theory is applicable (μ*_T_* > 1); otherwise the DMT model should be used (μ*_T_* < 1). A description of the transition between these limits (μ*_T_* ≈ 1) was provided by Müller *et al.* [178], Maugis [179] and Johnson and Greenwood [180]. Xu *et al*. [181] suggested a modified Tabor parameter for the JKR-DMT transition in the presence of a liquid meniscus, as did Fogden and White [182], who introduced a parameter to include the Kelvin radius for the JKR–DMT transition. This topic was also addressed by Maugis and Gauthiermanuel [183] who included capillary effects within the framework that Maugis had previously established. The Maugis-Dugdale (M-D) theory can be expressed mainly in terms of dimensionless parameters, such as an elasticity parameter, λ, related to μ*_T_* by:

(5.32) $$\lambda =\frac{2.06}{z_{0}}{\left(\frac{\varpi^{2}R}{\pi K^{2}}\right)}^{1/3}=1.16\mu_{T}$$

Using this theory, Johnson [184] constructed an adhesion map with co-ordinates μ and *F̄*, where *F̄* is the reduced load given by:

(5.33) $$\bar{F}=\frac{F_{adh}}{\pi \varpi R}$$

Significant adhesion has been encountered in nanotribology where the contact size may be measured in nanometers. Most practical applications fall in the JKR zone of the map, but a small radius of an AFM tip, for example, leads to operating values of λ which are in the Maugis-Dugdale transition zone. For AFM systems Carpick *et al.* reported λ ≅ 0.8 [185] while Lantz *et al.* [186] found λ ≅ 0.2→0.3. Upon inserting appropriate estimates for *ϖ*, *K*, and *R* in Equation 5.33, an approximate choice between Equations 5.28 and 5.30 can be made. Carpick *et al.* [187] presented a conversion table between λ and associated values of *F̄*, which can be estimated using an empirical equation [187]:

(5.34) $$\bar{F}=-\frac{7}{4}+\frac{1}{4}\left(\frac{4.04\lambda^{1.4}-1}{4.04\lambda^{1.4}+1}\right)$$

Using Equations 5.33 and 5.34, one obtains empirical values for the adhesion force. For λ values in the literature the expression of the adhesion force is approximately:

(5.35) $$F_{adh}^{M-D}\approx \left(1.9\overset{\alpha_{adh}}{↔}1.6\right)\pi R\varpi_{ikj}$$

Shi *et al.* [188] showed a comparison of the three models, viz. JKR, DMT and M–D and the influence of the dimensionless load parameter. Both the dimensionless load parameter, *F̄*, and the transition parameter affect the contact area at micro/nano-scale and should not be ignored in nano-indentation tests. Patrick *et al.* [189] also demonstrated the accuracy of the three models using molecular dynamics simulations. These simulations, experimentally verified by Lantz *et al.* [186], indicate that an exact determination of the work of adhesion, *ϖ_ijk_*, only from force–distance curves, is impossible (for determination of work of adhesion see section V-3). For the slope of the contact line and the jump-off-contact depend on each other in a way described by the parameter λ, but in order to calculate λ both *ϖ_ijk_* and *K* must be known. When the surfaces are rough, the M-D model (Equation 5.35) is no longer valid, the vdW adhesiveness between a spherical particle and a rough substrate can be calculated with the Rumpf formula [190]:

(5.36) $$F_{adh}=\frac{A\;R}{6{D_{o}}^{2}}\left[\frac{1}{1+\frac{R}{r_{a}}}+\frac{1}{{\left(1+\frac{r_{a}}{D_{o}}\right)}^{2}}\right]$$

where *A* is the Hamaker constant and *D_o_* is the “cut off” distance, which represents the effective separation between two surfaces or particles in contact (interatomic spacing).The first term in the brackets stands for the interaction between the particle with radius *R* and the semispherical asperity with radius *r_a_*. The second term represents the “noncontact” force between the particle and the flat surface with a separation *r* + *D_o_* ≈ *r*.

The asperity on surfaces is not hemispherical. Rabinovich *et al*. [190] suggested an approximation for this case, using root-mean-square (rms) roughness and the peak-to-peak distance, which can be measured with an AFM. Within such approximation, the adhesion force is:

(5.37) $$F_{adh}=\frac{A_{1,2}\;R}{6{D_{o}}^{2}}\left[\frac{1}{1+\frac{R}{r_{a}}}+\frac{1}{{\left(1+\frac{z_{\text{max}}}{D_{o}}\right)}^{2}}\right]$$

where the separation distance between the particle and the flat substrate is approximated by *z*_max_ = 1.817 rms. The other terms are defined as in Equation 5.36. Unfortunately, the rms values depend on the scanned areas, and bring additional difficulties for estimating the force. The model described the decrease in adhesion for increasing roughness on titanium deposited on silicon wafers [191] and served to analyze adhesion force of glass and lactose particles from rough surfaces, e.g., polycarbonate and acrylonitrile-butadiene-styrene [192]. Zhou *et al.* [193] studied the influence from electrostatic interactions and roughness of particles and substrates on adhesion. They concluded that the vdW adhesion forces can be drastically reduced if two conditions are fulfilled: (*i*) the surface must be appropriately rough and (*ii*) the peak-to-peak distance between rough spots must be in the right proportion to minimize the density between the two adhering partners. This topic was also investigated by other research groups [81,194–196].

The measured adhesion force for two rough surfaces in contact may also be expressed as [196]:

(5.38) $$F_{adh}=\alpha_{\text{int}}n\pi a_{o}^{2}$$

where *n* denotes the number of asperity contacts and *α_int_* is the intrinsic adhesiveness, defined as the adhesion force for a unit effective surface area [196]. This equation provides the explanation for the high variability of AFM adhesion results. Schaefer *et al.* [197,198] showed that surface-particle adhesion with the colloid probe technique was much lower than expected for a simple sphere-plate geometry, which was attributed to the roughness of the contacting surfaces. Other roughness models for particle adhesion have been studied, where rough surfaces and interactions were modeled with fractals, boundary element technique, semi-empirical models, and using fast Fourier transform algorithms [199–210].

#### 5.1.3. Work of Adhesion and Surface Energy

##### 5.1.3.1. Wettability

**Figure 10:**
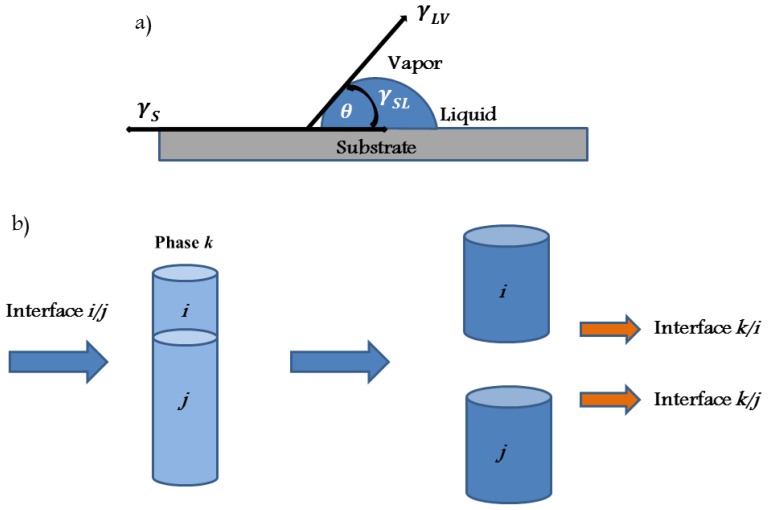
(**a**) Schematic representation of a liquid drop on a solid surface. Equilibrium is characterized by the three surface tensions acting at the liquid (*i*): solid, (*j*): vapor, (*k*): contact line [211]; (**b**) The separation of two phases.

Wetting is the ability of a liquid to maintain contact with a solid surface resulting from intermolecular interactions when the two are brought together. The degree of wetting (wettability) is determined by a force balance between adhesive and cohesive forces [23]. Adhesive forces between a liquid and solid cause a liquid drop to spread across the surface. Cohesive forces within the liquid cause the drop to ball up and avoid contact with the surface. If a liquid is forced to cover a substrate, which it does not wet, under equilibrium conditions it will break up and form drops. This phenomenon is called dewetting [211]. Dewetting is driven by the balance of capillary forces acting at the three phase contact line (substrate, liquid and air), as shown in Figure 10. The balance between the liquid–air, liquid–substrate, and substrate–air interfacial tensions determines this capillary force, being related to the contact angle. If the interfacial tension between the solid and the liquid is higher than the surface energy of the solid, dewetting will happen because it is energetically more favorable to remove the liquid from the substrate than to keep it spread on the substrate [211]. The position of the triple interface will change in response to the horizontal components of the interfacial tensions acting on it. At equilibrium these tensions will be in balance and one obtains the so-called Young Equation [212]:

(5.39) $$\gamma_{LV}=\gamma_{SL}+\gamma_{LV}\text{cos}\theta$$

Although Equation 5.39 describes the equilibrium contact angle in terms of the interfacial tensions involved, it gives no real insight into the reason why a certain value of contact angle is reached. Some surfaces have very high contact angles for water, while for others it is negligible. As already mentioned, an understanding of the origin of contact angle requires knowledge of the balance of forces between molecules in the liquid drop (cohesive forces), and those between the liquid molecules and the surface (adhesive forces). A surface with primarily polar groups, such as hydroxyl groups, will have a good affinity for water and, therefore, strong adhesive forces and a low contact angle (hydrophilic surfaces). If the surface is made up of non-polar groups, which is common for polymer surfaces or surfaces covered by an organic layer, the surface is hydrophobic, and the contact angle will be large. Wetting is determined by the equilibrium contact angle, *θ*. If *θ* < 90°, the liquid is said to wet the surface; if *θ* = 0° there is complete or perfect wetting and if *θ* > 90°, the liquid does not wet the surface. Contact angles of 180° are not found, as there are always interactions between the liquid and the solid.

##### 5.1.3.2. Work of Adhesion

If two phases (*i* and *j*) in contact are pulled apart inside a third phase *k*, the original interface is destroyed and two new interfaces are formed (see Figure 10b).The work energy per unit area in performing this operation is called the *work of adhesion*, *ϖ*. There is a contribution from each interface removed from or added to the system:

(5.40) $$\varpi_{ikj}=\gamma_{ik}+\gamma_{jk}-\gamma_{ij}$$

If, instead of two distinct phases, a column of single liquid is pulled apart, the work of cohesion is:

(5.41) $$\varpi_{iki}=2\gamma_{ik}\;(\text{two equal surfaces},i,\text{in a medium},k)$$

where γ*_ik_* is the surface tension and *ϖ_iki_* is the free energy of cohesion in vacuum. Thus, the work of adhesion is the energy to create two new surfaces from one interface (Equation 5.40), while the work of cohesion is the energy to create two new surfaces (Equation 5.41). Surface tension (or surface free energy per unit area) of a liquid or solid is defined as half of the free energy change due to cohesion of the material in vacuum [213,214]. When one of the phases is a solid, the expression for the work of adhesion (Equation 5.40) can be combined with the equation for the contact angle (Equation 5.39):

(5.42) $$\varpi_{ij}=\gamma_{ik}+\gamma_{jk}-\gamma_{ij}=\gamma_{ik}\text{cos}(1+\text{cos}\theta )$$

This is commonly known as the equation of Young-Dupré [212,215]. Its significance is that it relates the work of adhesion to the really measured quantities γ*_ik_* and θ, rather than to the inaccessible interfacial tensions involving the solid surface. A common approach to treating solid surface energies is that of expressing any surface tension (usually against air) as the sum of components due to dispersion forces (γ*^d^*) and polar (e.g., hydrogen bonding) forces (γ*^p^*) [216]. Thus, the interfacial tension between two phases *i* and *j* is expressed in terms of the two components for each phase:

(5.43) $$\gamma_{j}^{i}=\gamma_{i}+\gamma_{j}-2\sqrt{\gamma_{i}^{d}\gamma_{j}^{d}}-2\sqrt{\gamma_{i}^{p}\gamma_{j}^{p}}$$

Four cases arise in describing the work of adhesion:

- unequal surfaces *i* and *j* in contact in vapor (*V*)
  (5.44) $$\varpi_{iVj}=2\left(\sqrt{\gamma_{i}^{d}\gamma_{j}^{d}}+\sqrt{\gamma_{i}^{p}\gamma_{j}^{p}}\right)$$
- equal surfaces *i* and *j* in contact in vapor
  (5.45) $$\varpi_{iVi}=2\left(\gamma_{iV}^{d}+\gamma_{iV}^{p}\right)$$
- unequal surfaces *i* and *j* in contact with a liquid (L) or immersed solids [217]
  (5.46) $$\varpi_{iLj}=2\left\{\left(\gamma_{L}^{d}+\gamma_{L}^{p}\right)-\left[\sqrt{\gamma_{i}^{d}\gamma_{L}^{d}}+\sqrt{\gamma_{i}^{p}\gamma_{L}^{p}}\right]-\left[\sqrt{\gamma_{j}^{d}\gamma_{L}^{d}}+\sqrt{\gamma_{j}^{p}\gamma_{L}^{p}}\right]+\left[\sqrt{\gamma_{i}^{d}\gamma_{j}^{d}}+\sqrt{\gamma_{i}^{p}\gamma_{j}^{p}}\right]\right\}$$
- equal surfaces *i* and *i* in contact under a liquid
  (5.47) $$\varpi_{iLi}=2\left\{\left(\gamma_{L}^{d}+\gamma_{L}^{p}\right)-2\left[\sqrt{\gamma_{i}^{d}\gamma_{L}^{d}}+\sqrt{\gamma_{i}^{p}\gamma_{l}^{p}}\right]+\left[\sqrt{\gamma_{i}^{d}\gamma_{i}^{d}}+\sqrt{\gamma_{i}^{p}\gamma_{i}^{p}}\right]\right\}$$

The cross-dispersion as well as the cross-polar interactions are more appropriately expressed using harmonic rather than geometric means [218], so that:

(5.48) $$\varpi_{ij}=\frac{4\gamma_{i}^{d}\gamma_{j}^{d}}{\gamma_{i}^{d}+\gamma_{j}^{d}}+\frac{4\gamma_{i}^{p}\gamma_{j}^{p}}{\gamma_{i}^{p}+\gamma_{j}^{p}}$$

The so called polarity matching $\left(\frac{\gamma_{S}^{p}}{\gamma_{S}^{d}}=\frac{\gamma_{L}^{p}}{\gamma_{L}^{d}}\right)$ states that optimum adhesives for non-polar materials are non-polar while those for polar materials are polar (Bruyne’s rule).The adherent surface may be characterized, *i.e.*, given values of γ*_S_^d^* and γ*_S_^p^*, by using a pair of probe liquids, each with known γ*_L_^d^* and γ*_L_^p^* values, which are tabulated or may be determined from interfacial tension measurements against polar liquids. The work of adhesion of these probe liquids against the solid is measured from their contact angles against the solid and their surface tensions [218]:

(5.49) $$\varpi_{i}=\gamma_{i}(1+\text{cos}\theta_{i})=2\sqrt{\gamma_{i}^{d}\gamma_{s}^{d}}+2\sqrt{\gamma_{i}^{p}\gamma_{s}^{p}}\text{and}$$

(5.50) $$\varpi_{j}=\gamma_{j}(1+\text{cos}\theta_{j})=2\sqrt{\gamma_{j}^{d}\gamma_{s}^{d}}+2\sqrt{\gamma_{j}^{p}\gamma_{s}^{p}}$$

providing two equations for the variables γ*_S_^d^* and γ*_S_^p^*. The polarity matching principle has achieved some measure of qualitative success in estimating contact adhesion.

##### 5.1.3.3. Surface Tension

The total surface tensions of liquids are easily determined by a variety of methods [219–222] or can in many cases be found in published tables [23,223]. In an attempt to relate components to the chemical nature of the phase, van Oss *et al.* [224] suggested that the polar component could be better described in terms of acid-base interactions. Thus, an interfacial tension can be expressed as γ*=*γ*^LW^* γ*^AB^*, where LW and AB are Lifshitz-vdW and acid-base interactions. Unlike γ^LW^, the polar London-vdW component, the acid-base component γ^AB^ comprises two non-additive parameters. These acid-base interactions are complementary in nature and are the electron-acceptor surface tension parameter (γ+) and the electron-donor surface tension parameter (γ^−^). The total interfacial between two phases is [225]:

(5.51) $$\gamma_{SL}={\left(\sqrt{\gamma_{S}^{LW}}-\sqrt{\gamma_{L}^{LW}}\right)}^{2}+2\left(\sqrt{\gamma_{L}^{+}\gamma_{L}^{-}}+\sqrt{\gamma_{S}^{+}\gamma_{S}^{-}}-\sqrt{\gamma_{S}^{+}\gamma_{L}^{-}}-\sqrt{\gamma_{S}^{-}\gamma_{L}^{+}}\right)$$

For LW interactions, the free energies of interaction between two identical or different materials, in vacuum or immersed in a liquid, can be directly obtained from surface tensions of the materials. If one wishes to characterize the wetting behavior of a particular liquid-solid pair, one needs only to obtain the contact angle θ for three liquids (two of which must be polar) with known γ*_L_^LW^*, γ*_L_*^+^, and values γ*_L_*^−^, and then use Equation 5.52 [226].

(5.52) $$(1+\text{cos}\theta )\gamma_{L}=2\gamma_{SL}$$

Another approach includes the Owens-Wendt theory [227], in which it is assumed that the polar and non-polar components of surface energy can be combined as a geometric average: $\gamma_{L}(1+\text{cos}\theta )=2\left(\sqrt{\gamma_{s}^{d}\gamma_{L}^{d}}+\sqrt{\gamma_{s}^{p}\gamma_{L}^{p}}\right)$. Dividing this relationship by $2\sqrt{\gamma_{L}^{d}}$:

(5.53) $$\frac{\gamma_{L}(1+\text{cos}\theta )}{2\sqrt{\gamma_{L}^{d}}}=\sqrt{\gamma_{s}^{d}}+\sqrt{\frac{\gamma_{L}^{p}}{\gamma_{L}^{d}}}\sqrt{\gamma_{s}^{p}}$$

Hence, a graph of $\frac{\gamma_{L}(1+\text{cos}\;\theta )}{2\sqrt{\gamma_{L}^{d}}}$ versus $\sqrt{\gamma_{L}^{p}/\gamma_{L}^{d}}$ leads to a straight line with intercept $\sqrt{\gamma_{s}^{d}}$ and angular coefficient equal to $\sqrt{\gamma_{s}^{p}}$, which is the surface energy of the material.

The surface energy between two surfaces can also be determined from the intermolecular forces. The pairwise summation of energies between all the atoms of one medium with all the atoms of the other medium, for vdW forces, leads to the interaction energy between two identical media as $\varpi =-\frac{A}{12\pi D^{2}}$ (see Table 3). Had the summation been carried out between all atoms, including atoms in the same medium, one should obtain two additional energy terms: $\varpi =-C+\frac{A}{12\pi D^{2}}$, where the constant is simply the bulk cohesive energy [21]. The positive term arises from the unsaturated bonds at the two surfaces. This term is always positive, indicating that a free liquid tends to minimize surface energy by minimizing its surface area [21]. Thus, besides the bulk energy, the total energy of two planar surfaces at a distance *D* is given by $\varpi =\frac{A}{12\pi D_{o}^{2}}\left(1-\frac{D_{o}^{2}}{D^{2}}\right)$ per unit area. At *D = D_o_* (two surfaces in contact), *ϖ =* 0, while for *D =* ∞ (two isolated surfaces), $\varpi =\frac{A}{12\pi D_{o}^{2}}=2\gamma$ or:

(5.54) $$\gamma^{LW}=\frac{A}{24\pi D_{o}^{2}}$$

In other words, the surface energy γ equals half the energy needed to separate two flat surfaces from contact to infinity, viz. it is half the adhesion energy. To use Equation 5.52 for calculating surface energies a “cut off” distance *D_0_* should be found, which represents the effective separation between two surfaces or particles in contact. *D_0_* is substantially less than the interatomic or intermolecular center-to-center distance *r* (see Equation 5.10) [228]. In order to estimate the strength of interaction forces and energies, as in Table 1, a cut-off distance of *D_0_* ≈ 0.165 nm is sometimes used for macroscopic surfaces, and *D_0_* ≈ 0.3–0.4 nm for individual atoms or small molecules [43]. However, the use of molecular diameters, *i.e.*, *D_0_* = σ, does not give reasonable results in Equation 5.54. Instead, by considering that there should be nine neighbor molecules in a planar, closely-packed structure, a semi-empirical equation was developed for the cut-distance for *D_0_* which is significantly lower than σ [229]:

(5.55) $$\gamma^{LW}=\frac{A}{24\pi {\left(\sigma /2.5\right)}^{2}}$$

Thus, one may now test Equations 5.52 and 5.53 as to how well they predict surface energies determined with AFM or contact angle measurements. Equations 5.52 and 5.53 are not applicable only for highly polar H-bonding liquids such as formamide, glycerol, and glycol, for which the surface energies are underestimated. These equations are reliable for liquids interacting only with dispersion forces to within ±20% [229]. The equations cited previously do not describe the effect of surface topography and treatment on the surface energy or contact angle, although these parameters are greatly affected by the roughness of the solid surfaces [163,230,231].

Another method for measuring surface energy is to AFM and the mechanics of the contacts (JKR and DMT). The difference between JKR and DMT models lies in assuming the nature of forces acting between particle and substrate. Both models describe the correlation between pull-off force (*F_pull–off_*) and work of adhesion (ϖ) through a simple analytical equation (Equation 5.31). Thus, equaling Equation 5.31 with Equation 5.39 [232,233]one obtain:

(5.56) $$\gamma_{ik}=\frac{F_{pull-off}}{-\alpha_{adh}2\pi R_{t}}$$

where α*_adh_* = 2 in the DMT model and α*_adh_* = 1.5 in the JKR model. Thus, knowing which mechanical model applies to a particular system under study, and setting operation conditions during AFM measurements that satisfy the particular model, γ*_ik_* can be determined.

### 5.2. Interactions in Ambient Conditions

#### 5.2.1. The Thin Water Film

If a liquid vapor is introduced in the system, the surface energy of the solids may be modified by adsorption. At a certain relative vapor pressure capillary condensation occurs at the point of contact between the tip and sample. An annulus of capillary condensate forms around the tip, yielding a capillary force that could be the main contribution in the measured pull-on and pull-off force. For measurements under ambient conditions in which a layer of adsorbed water is formed on the sample surface, two main nanoscale effects have to be considered: the disjoining pressure, Π, experienced by thin films, and in the case of non-flat interfaces the Laplace pressure (*L*), which determines the curvature of the adsorbed layer. The disjoining pressure is the interaction force per unit area between flat liquid/gas interfaces, and is induced by long-range interactions. For films of micrometer thickness, the disjoining pressure is negligible, but it must be considered for films in the range from 2 nm to 100 nm thick. Several forces contribute to the disjoining pressure, as follows [86]: Π(*t*) = Π*_disp._* + Π*_EDL_* + Π*_ads_* + Π*_HB_* + Π*_st_*, where Π*_disp_* arises from the vdW or London dispersion forces, Π*_EDL_* are the electrical double layer interaction forces, Π*_ads._* arises from the solute adsorption, Π*_HB_* is due to hydrogen bonding and Π*_st_* arises from steric forces.

For some systems, the vdW interaction dominates and the disjoining pressure for a film of thickness, *t*, can be written as:

(5.57) $$\Pi (t)=\frac{A_{SLV}}{6\pi t^{3}}$$

**Figure 11:**
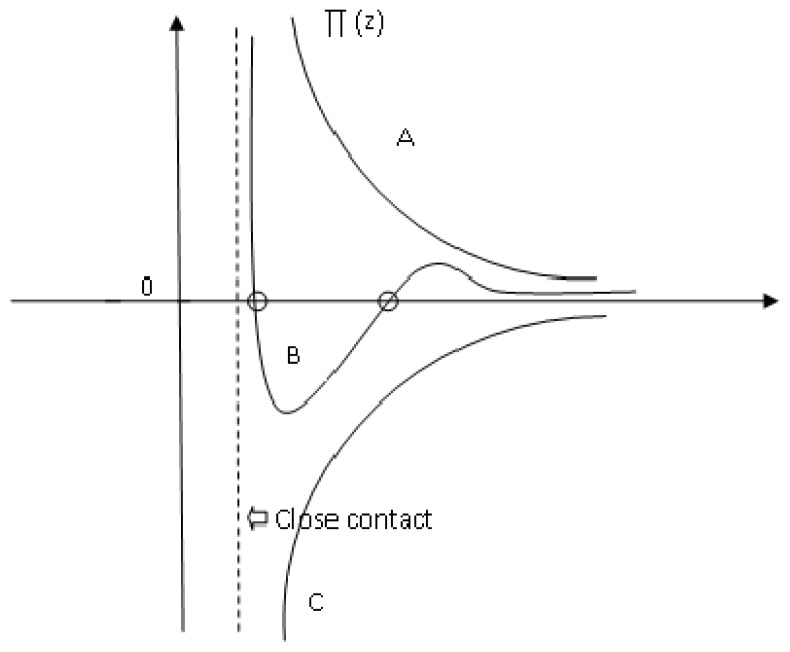
Dependence of disjoining pressure on film thickness and type of force involved. Curve (**A**) corresponds to a repulsive force and is a wetting case. Curve (**C**) is an attractive force and a non-wetting situation, and curve (**B**) corresponds to a metastable film (Reproduced by permission of Elsevier [234]).

Depending on the sign of the Hamaker constant, *A*_SLV_, *i.e*., on the dielectric properties of the three media, the force responsible for the disjoining pressure can be attractive, repulsive or a mixture of both, as shown in Figure 11. Curve A is typical of a stable film (wetting), curve C corresponds to an unstable film (non-wetting), and curve B corresponds to a metastable film.

Another possible origin for the disjoining pressure is the so-called repulsive double layer force, which is relevant for charged surfaces or ionic solutions [23]. For an electrolyte solution, the disjoining pressure can be described by:

(5.58) $$\Pi (t)=K_{s}\;\text{exp}(-2\chi t)$$

Where χ is the Debye screening length of ions in the solution and *K_s_* is a constant factor related to the surface charge. In the case of pure water, the ions come mainly from the solid surface, their concentration being very low. DLVO theory includes the effects of both long-range forces, namely, the vdW and the double layer, for the disjoining pressure. Hence, the Π ( *z*) plot can take complicated shapes owing to the superposition of the two contributions, as illustrated in Figure 11. In effect, the disjoining pressure displaces the gas/liquid interface away from or towards the solid/liquid interface. This implies a change in the internal energy of the system and, as a consequence, a change in chemical potential of the liquid, which will change from zero to μ*_liq_* = Π(*t*)/*n_1_*. In order to keep the equilibrium between vapor and liquid phases, both chemical potentials must be equal: μ*_vapor_* = *kT* ln (*n_ν_/n_sat_*) = −Π(*t*)/*n_l_*. From these expressions, it is possible to obtain the film thickness for a given temperature and gas density.

Considering only the vdW contribution to the disjoining pressure and a hydrophilic substrate, the thickness of the water film can be approximated by:

(5.59) $$t={\left(\frac{kT}{\eta \;\text{ln}(p_{s}/p)}\right)}^{1/3}$$

where η is related to the Hamaker constant between water and the substrate and the density of water ρ by η *= A_sw_* (6πρ), and *p* and *p_s_* are the partial vapor pressure and saturated vapor pressure of water, *i.e.*, relative vapor pressure [235].

As an AFM tip approaches the substrate, the capillary force on the tip is initially near zero until the tip contacts the surface of the water film. When contact is made, water is adsorbed around the tip to form a meniscus bridge between the tip and the substrate. Therefore, the force curve (pull-on force) depends directly on the height of the water film adsorbed on the substrate. The minimum thickness required for the water to spread [236] is:

(5.60) $$s=a_{m}{\left(\frac{\gamma_{SV}}{\varsigma }\right)}^{1/2}$$

where *a_m_* is a molecular length given by *a_m_* = *A/*6πγ*_SV_* [237], ζ is the spreading coefficient given by ς = γ*_SV_* − γ*_SL_* − γ*_LV_*, and γ_sv_ is the solid-vacuum interfacial energy. The formation of the capillary neck requires a minimum height of the water film. No capillary neck forms between two surfaces until the water film thickness reaches the minimum thickness, *s*. Clearly, if ς is negative then γ*_SV_* < γ*_SL_* < γ*_LV_*, and the liquid will not spread on the substrate but will form a finite contact angle θ*_c_* given by Young’s equation [212]: γ*_SV_* = γ*_SL_* + γ*_LV_*, cosθ*_c_*. To calculate θ*_c_*, using the Hamaker constant, one can use the Hough–White equation (valid only for alkanes with carbon number above 10) [87]:

(5.61) $$\text{cos}\;\theta_{c}\approx \frac{2A_{SVL}}{A_{LVL}}-1$$

Techniques used to analyze water films on surfaces include ellipsometry [238], the surface force apparatus [239] and atomic force microscopy [240–243]. Forcada *et al.* [244] measured the thicknesses of solid-supported thin lubricant films using AFM, where the differences between the thicknesses measured with the force microscope and ellipsometric thicknesses were explained by the appearance of an instability in the liquid film. The theoretical description also predicts the dependence of these differences on the film thickness.

**Figure 12:**
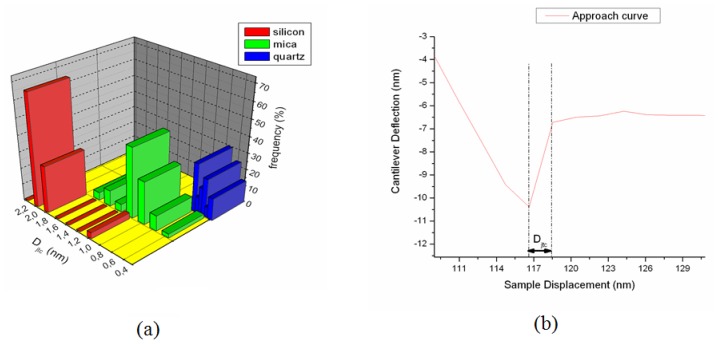
(**a**) Histogram illustrating the values of jump-to-contact distance in air (RH ≈ 70%) for sample surfaces of mica, quartz and silicon; (**b**) Typical force curve enlarged in the attractive region, illustrating the thickness of the liquid film determined by AFS (*k*_c_ ≈ 0.13 N/m) (Reproduced by permission of Taylor and Francis Ltd. [29]).

In our group, measurements of water layer thickness were carried out on mica, silica and silicon substrates. Figure 12a shows the thickness of the liquid film determined by AFM and the influence of the type of substrate. Figure 12b shows a force curve enlarged in the attractive region (approach curve) identifying the jump-to-contact distance (Djct). The thickness of the liquid film is determined by Djtc values in the force curve (relative humidity, RH, ≈ 70%), since in “dryer” conditions (RH ≈ 36%) this distance decreases to *D_itc_^vdw^,* which is directly related to vdW forces (D_jtc_ = 2.1 nm). The theoretical value using Equation 5.16 is 1.4 nm for dry conditions.

#### 5.2.2. Attractive Interactions (vdW) (Pull-On Forces)

In 1969, Tabor and Winterton [47] measured the attractive vdW force-law (with SFA) between two glass or mica surfaces down to surface separations of *D* = 1.5 nm, and confirmed the Lifshitz theory of vdW forces. In 2003, Götzinger and Peukert [96] measured dispersive forces of particle-surface interactions by AFM and observed this force in the jump-in plot about 12 nm above the surface. In silicon surfaces, the vdW forces were approximately 4.5 nN in air and 0.7 nN in water. The vdW force between the surfaces (sphere-flat surface) covered by an adsorbed liquid film is given by (see Figure 4) [103]:

(5.62) $$F{\left(D\right)}_{sphere-plane}=-\frac{A_{eff}R}{6D^{2}}=\frac{R}{6}\left[\frac{A_{{jkj}^{\prime }}}{D^{2}}-\frac{A_{ikj}}{{\left(D+t\right)}^{2}}-\frac{A_{i^{\prime }kj}}{{\left(D+t^{\prime }\right)}^{2}}+\frac{A_{i^{\prime }ji}}{{\left(D+t+t^{\prime }\right)}^{2}}\right]$$

where *t_i_* and *t_j_* are the thickness of the water film adsorbed on surfaces *i* and *j*, whose values may be obtained with Equation 5.59.

At small separations, when $D\langle \langle (t+t^{\prime }),\;F{\left(D\right)}_{sphere-plane}=-\frac{A_{{jkj}^{\prime }}R}{6D^{2}}$, while at large separations, when $D\rangle \rangle (t+t^{\prime }),\;F{\left(D\right)}_{sphere-plane}=-\frac{A_{ikj}R}{6D^{2}}$. Thus, the vdW interactions are dominated by the properties of the bulk or substrate materials at large separations and by the properties of the adsorbed layers at separations less than the thickness of the layers. This means that the adhesion energies are largely determined by the properties of any adsorbed films even when these are only one monolayer thick [23].

#### 5.2.3. Adhesion Forces (Pull-Off Forces)

##### 5.2.3.1. Capillary Forces

**Figure 13:**
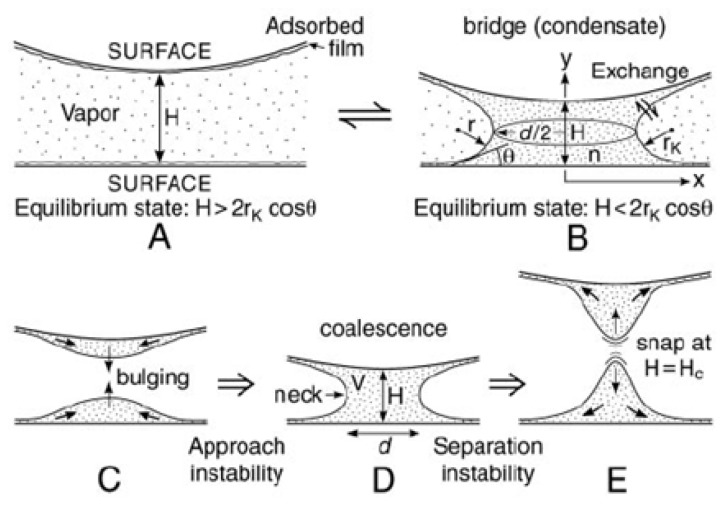
Liquid bridges between two surfaces (**A** and **B**). The equilibrium state of a liquid bridge is determined by thermodynamics. At equilibrium, the mean curvature of the liquid vapor interface of a bridge must equal the Kelvin radius *r*_k_. For wide necks and small θ, *d* >> *r* so that *r* ≈ *r*_k_, as drawn; (**C**–**E**) Transitions between the equilibrium states **A** and **B** usually occur via nonequilibrium processes. For example, because of the vdW force on approach or a Rayleigh instability on separation, fast mechanical instabilities may trigger bridge coalescence (**C**→**D**) or snapping (**D**→**E**). In such processes, the meniscus curvature is not determined by the Kelvin equation [245].

The formation and disappearance of liquid bridges between two surfaces can occur either through equilibrium or nonequilibrium processes. In the first case, the bridge molecules are in thermodynamic equilibrium with the surrounding vapor medium. In the second, chemical potential gradients result in material transfer [245]. Figure 13 illustrates some of the important concepts associated with the formation and disappearance of liquid bridges. The equilibrium states depicted in Figure 13A,B are uniquely determined by the Kelvin length (2*r_k_* cosθ). However, thermodynamics alone does not tell us how long it will take for transitions to occur between two equilibrium states. Such transitions typically occur by means of nonequilibrium processes involving material transfer. In the limit of very rapid processes, mechanical instabilities may occur at constant liquid volume, as depicted in Figure 13C–E.

**Figure 14:**
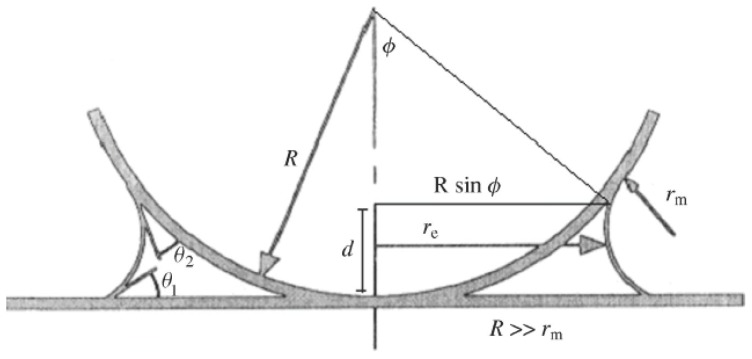
Schematic view of a water meniscus between a sphere with radius *R* and a plate (Reproduced by permission of Taylor and Francis Ltd. [29]).

At equilibrium, the meniscus curvature is related to the relative vapor pressure (relative humidity for water), *p*/*p_s_*, by the well-known Kelvin equation [246]:

(5.63) $${\left(\frac{1}{r_{1}}+\frac{1}{r_{2}}\right)}^{-1}=r_{k}=\frac{\gamma \nu_{m}}{RT\;\text{log}(p/p_{s})}$$

where *r*_k_ is the Kelvin radius, *R* is the gas constant, *T* is the temperature, *r*_1_ and *r*_2_ are the radius of the droplets, *p* is the actual vapor pressure, *p_s_* is the saturated vapor pressure, and *ν_m_* is the molar volume for water at 20 °C. Thus, for the spherical concave water meniscus in Figure 14, putting *r*_1_ = *r*_2_ = *r_m_*, *r_m_* = ∞ at *p*/*p_s_* = 1 and rm ≈ −0.5 nm *r_m_* ≈ −0.5*nm* at *p/p_s_* = 0.1 (10% relative humidity).

A consequence of the dependence of vapor pressure on curvature is the phenomenon of capillary condensation [247]. To investigate the effect of a liquid condensate on the adhesion force between a surface and a macroscopic sphere (SFA) or AFM tip (nanoscale), we consider here that the surfaces are smooth and non-porous (sphere and flat plate). The surfaces are surrounded by vapor, which is in chemical and thermal equilibrium with the liquid bridge. The volume of the condensation is expected to depend strongly on the relative vapor pressure, the distance between the surfaces as well as on the three-phase contact angle [248]. The contribution of capillary forces to the total interaction between an AFM tip and sample increases above a certain critical humidity [249]. Moreover, the adhesion force depends strongly on whether the substrate is hydrophilic or hydrophobic [250,251]. Hartholt *et al*. [252] reported a decreased mobility of glass particles when relative humidity increased from 45% to *ca*. 65%. For humidity above 65%, the particles became immobile, indicating increased capillary forces. Xu *et al.* [13] obtained a flat response in force at relative humidity less than 20%. The reason for adhesion after reaching the critical humidity is the capillary force due to the liquid meniscus formed near the contact area (see Figure 14).

The resulting capillary force between a plate and a sphere with radius R was calculated by O’Brien and Hermann [253] as:

(5.64) $$F_{Ad}^{C}=2\pi R_{t}\gamma_{l\nu }\;\left(\text{cos}\;\theta_{1}+\text{cos}\;\theta_{2}\right)$$

For two identical materials, *θ*_1_ *= θ*_2_, thus

(5.65) $$F_{Ad}^{C}=4\pi R_{t}\gamma_{l\nu }\;\text{cos}\;\theta$$

Equation 5.65 is useful for estimating the capillary force of a micro-contact; note that it is described as dependent only on the surface tension of bulk water, γ_lv_, and the contact angle, *θ*, but is independent of the solid–solid and solid–liquid interaction parameters. This equation does not explain the force transition experimentally observed in several papers as a function of the relative humidity. Miranda *et al.* [254] discovered by scanning polarization microscopy that the force instability was caused by a low coverage of water at the solid surface. The authors suggested that water, condensed from water vapor at room temperature on mica, forms a partially developed monolayer of an ice-like phase. They concluded that with decreasing humidity the ice-like water monolayer, which is formed around 90% RH, breaks into islands, until the water coverage is too low (20% RH).

When the relative humidity is less than 90%, both the water film thickness and the radius of the meniscus bridge are less than 10 nm [255], which is much smaller than the radius of the AFM tips used in many studies. In this case, the capillary force can be well described by:

(5.66) $$F_{Ad}^{C}=\frac{4\pi R_{t}\gamma_{lv}\text{cos}\;\theta }{\left(1+\frac{D}{d}\right)}$$

or for different contact angles:

(5.67) $$F_{Ad}^{C}=\frac{4\pi R_{t}\gamma_{l\nu }(\text{cos}\;\theta_{1}+\text{cos}\;\theta_{2})}{\left(1+\frac{D}{d}\right)}$$

where *D* is the distance between the tip and the substrate, *d* is the distance the tip extends into the water bridge and can be calculated by *d* = −1.08 cos*θ/*ln(RH) [256], where RH is the relative humidity. Generally, it is assumed that *D/d* is small and Equation 5.67 is reduced to Equation 5.65.

##### 5.2.3.2. Chemical Forces

The decrease in adhesive force during descending with increasing humidity can be understood as a superposition of physical and chemical phenomena (microscopic origin) [257]. The adhesive force on the tip is the sum of the capillary force and the interaction force between the two solid surfaces mediated by the water in the gaps between the contacting rough points. The solid-solid interaction is more complex than the capillary force. It may contain vdW forces, electrostatic forces, and chemical bonding. The presence of water in the gap can greatly change the nature of the interaction. Since the liquid water is at equilibrium with the water vapor, the chemical potential of the liquid in the gaps around the contacting asperities is [250]:

(5.68) $$\mu_{w}=kT\;\left(\text{ln}\frac{p}{p_{s}}\right)$$

From thermodynamics, the component of the chemical attractive force acting on the tip from the liquid in the gaps is given by [29,250]:

(5.69) $$F_{chem}=-\frac{\partial G}{\partial z}=-\frac{a_{L}}{\vartheta }\mu_{w}=-\frac{a_{L}}{\vartheta }kT\;\text{ln}\frac{p}{p_{s}}$$

where *G* is the Gibb’s free energy, *a_L_* is the area of the liquid film, and ϑ is the molar volume. Therefore, the force from water in the gaps becomes less attractive, *i.e*., more repulsive, with higher relative humidity.

Xu *et al.* [13] employed AFM adhesion measurements on mica surfaces to show that adhesion varies with humidity, which was confirmed with hydrophilic AFM tips on mica [251,258]. Pull-off force measurements with hydrophilic tips and hydrophobic substrates (coated silicon), or hydrophobic tip and hydrophilic substrates, are independent of relative humidity [236,259]. However, the force instability originates from the ability or inability of the water film to form a liquid joining the neck between the adjacent surfaces at high and low RH, respectively [29]. The decrease of the pull-off forces in high relative humidity for a hydrophilic tip was discussed by Binggeli and Mate [250,260]. The influence from varying air humidity on the pull-off force was also studied for particle-surface, particle-particle or surface-surface adhesion [164,261–266].

##### 5.2.3.3. Electric Forces

Burnham *et al.* [94] studied another type of Coulomb-like force which ascends from the patch charges distribution on the tip and sample, *i.e*., from regions of different surface charge density interacting through a long range force law. Consider a spherical tip and a flat sample, each one with its own initial surface charge, and each one with an image charge due to the presence of the other charged body, then the electric force is [29,94]:

(5.70) $$F_{elec}=\frac{1}{4\pi {ɛ}_{0}{ɛ}_{3}}\left[-\frac{Q_{t}}{4{\left(D+B\right)}^{2}}\left(\frac{{ɛ}_{2}-{ɛ}_{3}}{{ɛ}_{2}+{ɛ}_{3}}\right)+\frac{r_{c}Q_{t}Q_{s}}{Z\;{\left(2D+B+r_{c}\right)}^{2}}\left(\frac{{ɛ}_{1}-{ɛ}_{3}}{{ɛ}_{1}+{ɛ}_{3}}\right)\left(\frac{{ɛ}_{2}-{ɛ}_{3}}{{ɛ}_{2}+{ɛ}_{3}}\right)\right]$$

in which *Q_t_* represents an image charge associated with the tip, *D* is the tip–sample distance, *B* is the position of *Q_t_* within the tip, *Q_s_* represents an image charge on the sample surface, *r_c_* is the effective radius of curvature of the tip and *Z* is the position of *Q_s_*. The relative permittivities ɛ_1_, ɛ_2_ and ɛ_3_ correspond to the tip, sample and intervening medium, respectively.

The force is then independent of *D*, so that the patch charge effect is not noticed and vdW forces prevail. An AFM with an extremely curved tip retains the sensitivity to *D*. Recent extensions have led to methods to study surface-electrical variables: Kelvin Force Probe Microscopy, Scanning Capacitance Microscopy and Charge Detection Microscopy [29,267–271]. Once the tip and sample are exposed to air for quite a long time, no net charges are expected to persist and the electrostatic force is zero; nevertheless, capillary forces are present [29]. Through the control of the cleanliness of the surfaces (UHV environment), the adhesion due to vdW forces must become the principal attractive force amid uncharged, non-magnetic surfaces. In a solution, other forces related with double-layer, hydration and hydrophobicity need to be considered.

#### 5.6.4. Total Adhesion Forces

The total pull-off force measured in air by force spectroscopy or adhesion force between the AFM tip and flat inorganic surfaces is given by:

(5.71) $$F_{pull}^{air}=F_{cap}+F_{vdW}+F_{chem}+F_{elec}$$

In the absence of electrostatic charges:

(5.72) $$F_{pull}^{air}={\left\lceil \pi R_{t}\gamma_{LV}(\text{cos}\theta_{1}+\text{cos}\theta_{2})\frac{{\left(1+\text{cos}\emptyset \right)}^{2}}{\text{cos}\emptyset \;\left(1+\frac{D}{d}\right)}\right\rceil }_{cap}+{\left(\alpha R_{t}\varpi_{ikj}\right)}_{vdW}+{\left\lceil -\frac{a_{L}}{\vartheta }kT\;\text{ln}\frac{p}{p_{s}}\right\rceil }_{chem}$$

where the term $\frac{{\left(1+\text{cos}\;\phi \right)}^{2}}{\text{cos}\;\phi }$ is applicable only to small contacts or small asperity contacts [235], *i.e.*, large ϕ values (see Figure 13).

### 5.3. Interactions in Solution

#### 5.3.1. Screened vdW Forces in Electrolyte Solutions

Situations in which vdW forces alone determine the total interaction are restricted to a few simple systems, e.g., to interactions in a vacuum or to non-polar wetting films on surfaces, both already discussed in previous sections. In more complex systems long-range electrostatic forces are also involved, and the interplay between these two interactions has many consequences. For instance, clay particles and silt carried by rivers coagulate upon coming across the high salt concentration of the sea to form extensive deltas [272]. Electrostatic forces are also crucial in the behavior of biological systems [273], swelling of lipid bilayers in water [274,275], the unexpected stability of lattices at high salt concentrations [276], *etc*.

In an earlier section, we mentioned that the zero-frequency contribution to the vdW force is essentially an electrostatic interaction. Now, in any medium containing free charges, e.g., water containing free ions in solution or a conducting polymer containing free electrons, all electrostatic fields are screened due to polarization of these charges. In particular, highly polar H-bonding liquids, e.g., water, are known to cause a considerable reduction (by factors of 10 or more) in the vdW forces with respect to those for the vacuum level [277]. The tip/sample combination in a liquid medium can result in a more isotropic polarizable system than does the same combination in air or vacuum, with a resulting substantial reduction of the vdW forces. Across an electrolyte solution*,* the screened non-retarded Hamaker constant *A_e_* is given by [278]:

(5.73) $$A_{e}=A_{\nu =0}(2\kappa D)e^{-2\kappa D}+A_{\nu >o}$$

where κ is known as the Debye screening length and *D* is the distance between the surfaces. For example, in a 0.1 M aqueous NaCl solution the vdW screening length is *ca.* 0.5 nm, so that by *D* = 1 nm the zero-frequency contribution (*A_ν_*_=0_) has already fallen to about 10% of its value at *D* = 0. Thus, for inter-particle interactions across such a solution, at separations greater than 1 nm, the vdW interaction is effectively determined solely by the dispersion (*A_ν_*_>_*_0_*).

Hence, vdW interactions (pull-on force) in solution for the geometry described for AFM can be modeled as:

(5.74) $$F_{pull-on}=-\frac{A_{e}R}{6D^{2}}$$

This model indicates that vdW forces are reduced in salt solutions. However, as will be seen later, within the framework of the conventional DLVO colloid stability model, an increase in electrolyte concentration typically has more influence on the electrostatic interaction energy than on the vdW interaction energies, and results in increased attraction between two similar surfaces [279]. One concludes that solution chemistry can significantly affect interfacial forces between particles, even altering stability [277,280]. For example, when the muscovite mica is immersed in a polar liquid medium-like water, surface charges are induced on both the tip and the sample surface due to ionization, dissociation or spontaneous adsorption of charged species. To keep the electrical neutrality, opposite ionic species are held together closer to the tip/sample surface forming an electric doublelayer. When mica is placed in water, the mechanism of the double-layer formation is attributed to the K^+^ dissolution, as well as ionic exchange between K^+^ and H_3_O^+^ (or H^−^) [281]. The effects from ionic strength on the vdW interaction energy have been studied extensively [282–284]. Toikka *et al*. [285] showed that the double layer decreases the adhesion force, and that the apparent adhesion force depends on the pH of the solution. The authors confirmed the existence of this phenomenon by measuring adhesion forces in different pH solutions between an iron sample and a silica colloidal probe. Changes in vdW interactions as a function of electrolyte concentration can be attributed to screening of the non-dispersion portion of the Hamaker constant *A_ν_*_=0_, which was assumed not to be affected by electrolytes that cannot respond to high frequencies [278].

The fact that colloidal particles in liquid medium at high enough electrolyte concentration tend to form persistent aggregates through collisions caused by Brownian motion implies an interparticle attractive force (vdW force). In aqueous electrolyte solutions long-range electrical double layer forces also appear. The JKR theory of contact mechanics can serve as a reasonable basis for understanding adhesion forces (pull-off force or vdW adhesiveness) in an aqueous medium. Since it is based on energy balance, no adhesion is expected when the free energy of a double-layer per unit area *w_DL_* balances the interfacial surface tension γ*_SL_* Quantitatively, the pull-off force or adhesion force can be related to these two terms as follows [176]:

(5.75) $$F_{pull-off}\;=-\frac{3}{2}\pi R(\varpi_{iji}+2w_{DL})=-\frac{3}{2}\pi R\varpi_{iji}+\frac{5}{2}P_{DL}$$

where *P_DL_* ≈ 2π*Rw_DL_* is an additional load that has to be applied to a spherically shaped tip due to the presence of a double-layer and *ω̄_iki_* is the free energy of cohesion in vacuum. Thus, repulsion between like-charged surfaces (*P_DL_* > 0) will decrease the magnitude of the pull-off force compared to that given by the JKR theory.

#### 5.3.2. The DLVO Theory: vdW and Double-Layer Forces Acting Together

The first theories for the stability of hydrophobic colloids by Hamaker [286] and de Boer [287] were based on a balance between vdW attraction and electrical double-layer (DL) repulsion. These theories were further elaborated by Derjaguin [288], Derjaguin and Landau [289] and, independently, by Verwey and Overbeek [290], leading to the theory now known as DLVO theory [291]. At low salt concentration, the double-layer repulsion is sufficiently strong to keep the colloidal particles apart. With increasing salt concentration the electrostatic repulsion is increasingly screened [8]. At a certain concentration, the vdW attraction overcomes the repulsive electrostatic barrier and coagulation sets in.

**Figure 15:**
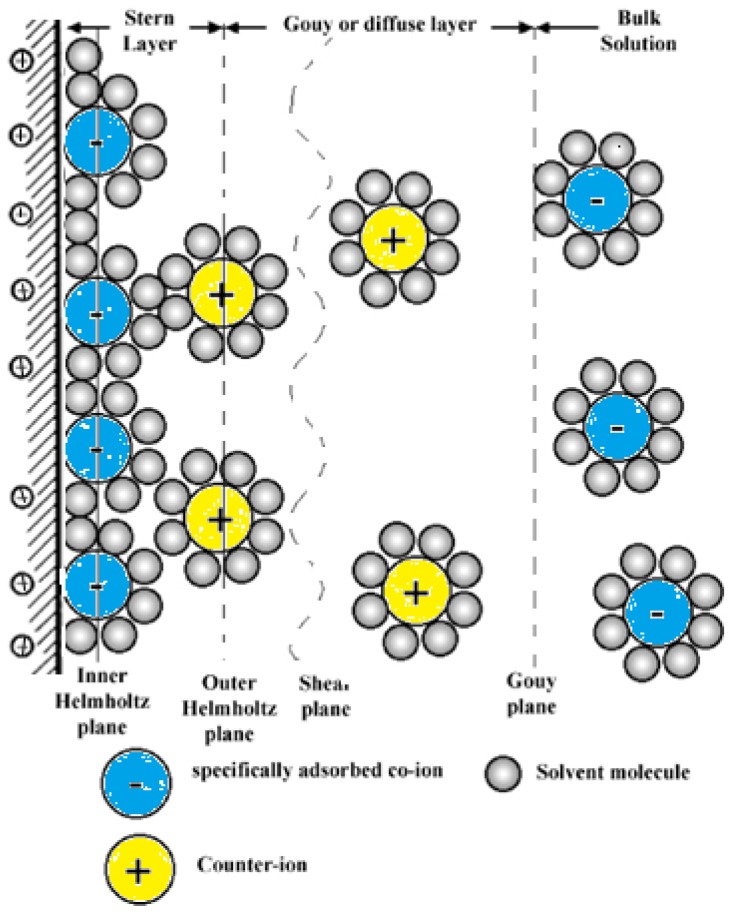
Schematic representation of the electric double layer (EDL). Overall model of the double-layer showing solvent molecules, counterions and specifically adsorbed co-ions.

The earliest model of the electrical double layer is usually attributed to von Helmholtz [292,293] (Figure 15), who treated the double layer mathematically as a simple capacitor, based on a physical model in which a single layer of ions is adsorbed at the surface. However, the classical theory for the electrical double-layer is the Gouy-Chapman-Stern [294–296] model, which combines the Helmholtz adsorbed layer with the Gouy-Chapman diffuse layer. It was proposed by Gouy [294], Chapman [295], and Debye and Huckel [297] that if a charged interface exists in a polar solvent, then ions of opposite charge are attracted to that surface. Entropy ensures that the ions do not all adsorb at the surface and form a crystal in many cases, leaving the ions to exist as a diffuse layer close to the charged surface [298]. In the stern layer, counterions are strongly adsorbed and they lower the electrical potential at points adjacent to the particle surface.

The form of the interaction is well known to all colloid scientists and is roughly exponential, given by:

(5.76) $$w_{DL}\propto \Psi_{o}^{2}\;\text{exp}(-\kappa D)$$

where *D* is the distance between the surfaces. The decay length is given by the inverse of the Debye-Huckel parameter, *κ*, and the intercept at zero separation is given by the surface potential, ψ_o_. The Debye length falls with increasing ionic strength and valence of the ions in the solution.

An analytical expression for measurements in AFM has been provided by Butt [28] on the basis of an equation of Parsegian and Gingell [299]. For a spherical tip and a flat sample, the double-layer force is given by [8]:

(5.77) $$F_{double}=\frac{2\pi R_{t}}{{ɛ}_{l}{ɛ}_{0}}\left[\left(\sigma_{T}^{2}+\sigma_{S}^{2}\right){\text{e}}^{2-\kappa_{D}D}+2\sigma_{T}\sigma_{S}{\text{e}}^{-\kappa_{D}D}\right]$$

where *R_t_* is the tip radius, κ*_D_* is the inverse of the Debye length, *D* is the distance between the surfaces, σ_T_ and σ*_S_* are the surface charge densities of tip and sample, respectively, ɛ_l_ is the dielectric constant of the liquid, and ɛ_0_ is the permittivity of free space.

**Figure 16:**
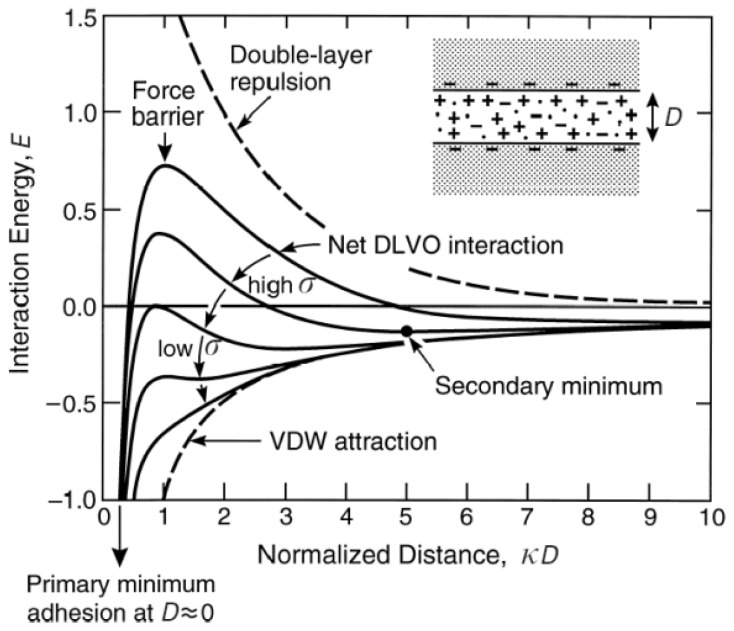
Schematic DLVO plots showing how the attractive vdW and repulsive electrostatic double-layer forces together determine the total interaction potential between two charged surfaces in aqueous electrolyte solutions at different surface charge densities *σ* or potentials Ψ*o* (Reproduced by permission of Elsevier [301]).

There are many approximate expressions for this interaction, some of which are in terms of the surface charge density σ. Others are related to the surface potential ψ_o_, which is easier to measure, or take into account “charge-regulation” effects where neither the surface charge nor the potential remain constant during an interaction [300]. In aqueous solutions, since vdW and electrostatic forces usually occur together, it is common practice to plot the two forces when describing the net interaction of two surfaces. Figure 16 shows how these forces may determine whether an interaction will be attractive or repulsive at a given separation. The subtleties in the plots arise because the forces have different distance dependencies—the one being a power law, the other an exponential [43].

**Figure 17:**
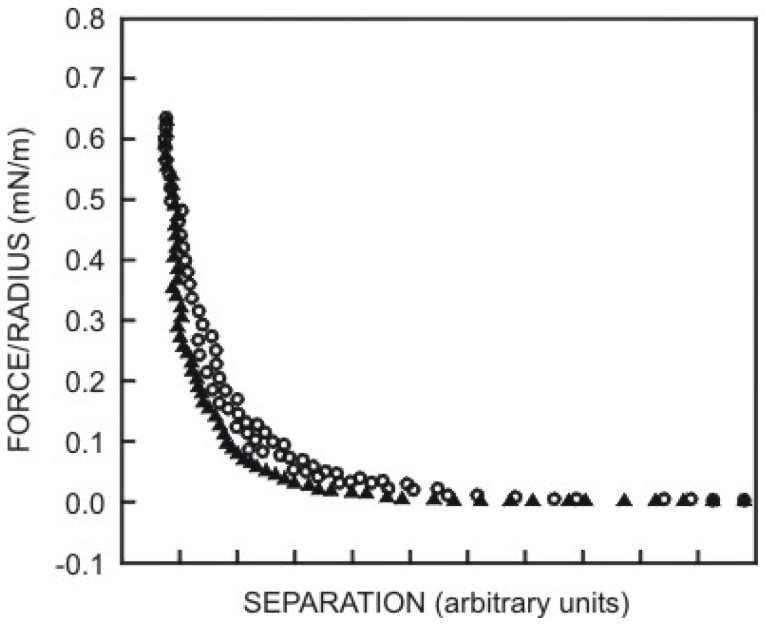
Forces measured between an α-alumina sphere (O) or silica sphere (Δ) and a flat Teflon AFTM surface in cyclohexane. The separation distance is in arbitrary units and the distance between the tick marks is 2 nm (Reproduced by permission of Elsevier [307]).

Hartley *et al.* [302] performed AFM measurements of vdW attraction between a silica sphere and a silica plate separated by 5–20 nm in aqueous solutions near the isoelectric pH 2.2–3.0 of silica. The measured forces were stronger than predicted by the non-retarded theory, which were attributed to weak electrostatic attraction. They also measured an attractive force between a silica sphere and a mica flat surface at pH 2.5, which was stronger than predicted for the vdW attraction, even including effects from retardation. Again, the discrepancy was attributed to a weak electrostatic attraction. Practically all of the measurements of vdW interactions mentioned above are for strong interactions experienced at separation distances of 20 nm or less where retardation effects are mild or unimportant. Interactions at larger separations tend to be severely retarded and much weaker. To measure accurately these weaker interactions, a different technique can be utilized. Bevan and Prieve [303] measured retarded vdW attraction with total internal reflection microscopy (TIRM) [304]. Teschke *et al.* [305] measured the force acting on the tip during its immersion in the double layer region for various tip-approaching velocities. Milling *et al*. [306] measured vdW repulsion between a gold sphere and a flat plate of poly(tetrafluoroethylene) separated by up to 20 nm of various liquids. The non-retarded Lifshitz theory was able to predict which interactions should be repulsive and that the force decays with the inverse square of the separation distance. A similar study was carried out by Lee and Sigmund [307], where the distance dependence of the measured forces between a flat Teflon AFTM foil and an α-alumina or amorphous silica sphere in cyclohexane agreed with the theoretically calculated forces, including the retardation contribution. Figure 17 shows that one can minimize vdW interactions by choosing a medium with dielectric constant and refractive index close to those of either the tip or the sample. Since both mica and Si_3_N_4_ have rather high refractive indices (1.57 and 1.97, respectively) [307], few liquids meet these criteria.

Borato *et al.* [308] showed that water is aged upon exposure to air, which was confirmed with impedance spectroscopy measurements made with taste sensors containing bare metal electrodes. Figure 17b shows that the force curves can be affected by water ageing in the liquid cell. For short periods, the curve displays a minimum with the distance between the tip of silicon nitrite (ɛ*_tip_* = 7.4) and a flat mica surface (ɛ*_mica_* = 5.4) [309], which indicated the predominance of attractive vdW interactions. For longer times, repulsive double-layer forces are practically purely repulsive (for *t*″). This is due to ageing of the water, which is accompanied by a change to lower pH values, and this increases the charge of the silicon nitride tip (whose isoelectric point is pH 6.3) [281], whereas mica is negatively charged. The net result is an increase in the repulsive, double-layer force.

**Figure 18:**
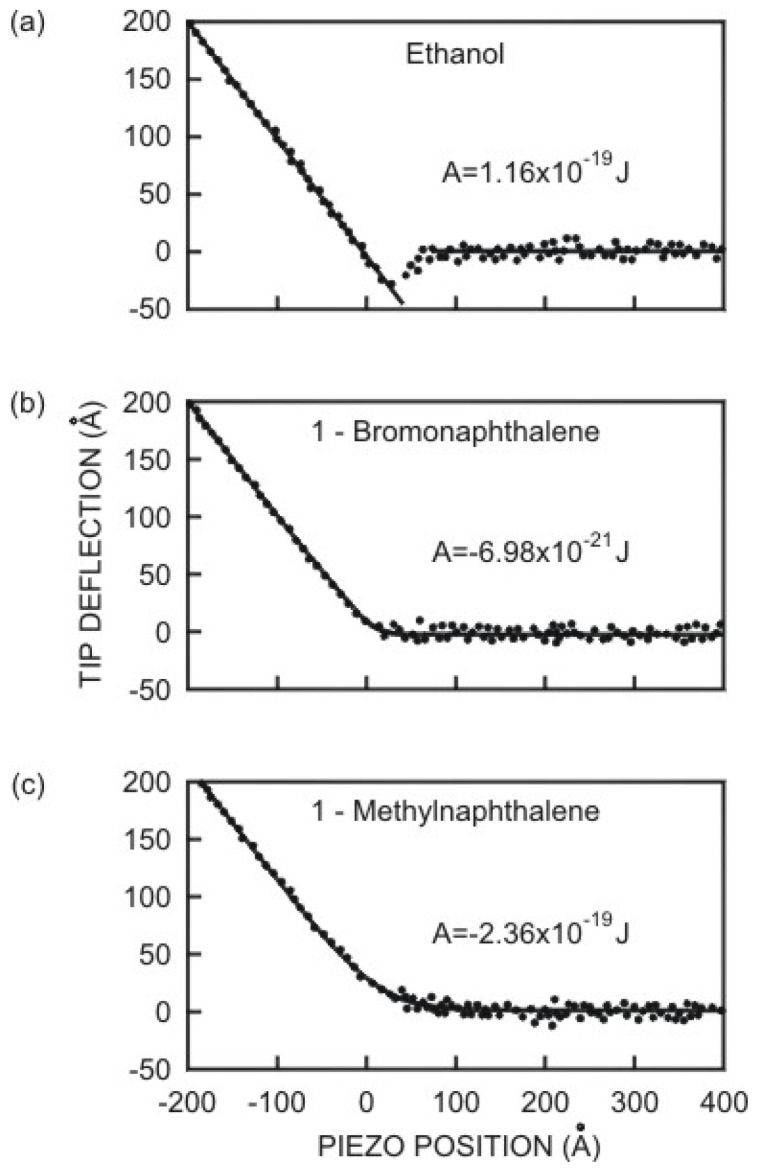
Representative deflection *vs*. piezo extension curves for a Si_3_N_4_ tip interacting with a mica sample across various media. In each case, the Hamaker constant was calculated from the fitting parameter: (**a**) Attractive interaction in ethanol (β *=* 44.9 Å); (**b**) small repulsive interaction in 1-bromonaphthalene (β = −22.2 Å); and (**c**) repulsive interaction in 1-methylnaphthalene (β = −56.9 Å) (Reproduced by permission of American Institute of Physics [114]).

Figure 18 shows representative curves for three media, viz. 1-bromonaphthalene, 1-methylnaphthalene and ethanol. These curves indicate the tip deflection as the tip approaches the sample. The vdW interaction is strongly attractive for ethanol, as one would expect. The other liquids yield a repulsive interaction.

#### 5.3.3. Non-DLVO Forces: vdW and Structural Forces Acting Together

Repulsive or attractive forces may also arise from structural forces referred to as solvation or hydration forces [279]. The continuum theories of vdW force and double-layer force cannot describe the mutual interaction of two surfaces approaching at distances below a few nanometers, because (*i*) they are not valid at small separations and (*ii*) other forces arise, which are named non-DLVO forces [224,310]. The latter can be roughly grouped into three categories: solvation forces, repulsive hydration forces and hydrophobic attractive forces. Derjaguin and Voropayeva [311] found an extra repulsive force between crossed platinum wires in aqueous solutions at high electrolyte concentrations. The stability of soap films [312] is an important example of a system where DLVO theory fails to explain the experimental observations of the thin film stability.

**Figure 19:**
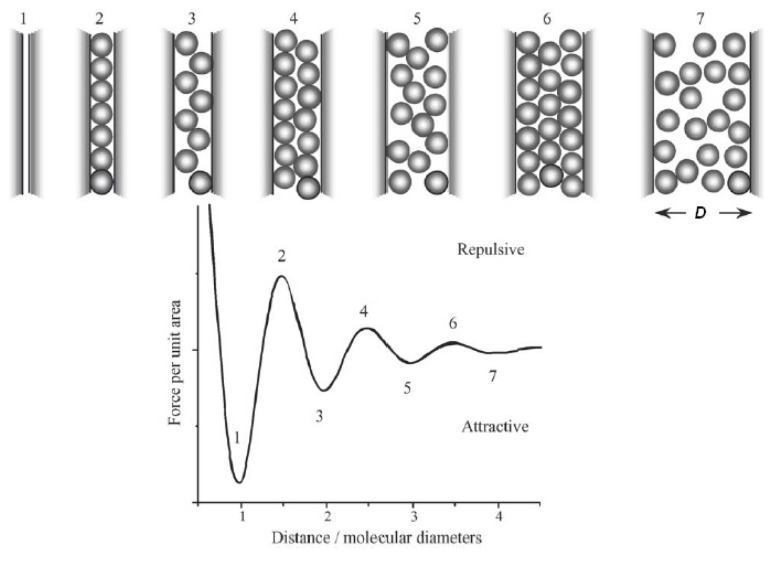
Schematic structure of a simple liquid confined between two parallel walls. The order changes drastically depending on distance, which results in an oscillatory force (adapted from Butt *et al*. and reproduced by permission of Elsevier [98]).

Solvation forces appear around particles suspended in an aqueous medium. This structured hydrogen-bonded network decays away from the surface. In most cases, these forces exhibit an oscillatory behavior, *i.e.*, the liquid density profiles and interaction potentials in liquids oscillate with the distance, with a periodicity close to the molecular size and with a range of a few molecular diameters [98]. In this range, the molecules are ordered in layers. When two surfaces approach each other, layers after layers are squeezed out of the closing gap. Here, attractive interactions between the wall and liquid molecules and the geometric constraining effect of the “hard wall” on these molecules force them to order (or structure) into quasi-discrete layers, as shown in Figure 19. This layering is reflected in an oscillatory density profile extending several molecule diameters into the liquid, as also illustrated in Figure 19 [313]. Such forces were termed solvation forces because they are a consequence of the adsorption of solvent molecules onto solid surfaces [314].

For simple spherical molecules between two hard, smooth surfaces the solvation force is usually a decaying oscillatory function of the distance (*D*). The solvation force between a sphere and planar surface can be calculated within the Derjaguin’s approximation [315,316]:

(5.78) $$F_{sol\nu }=F_{o}\;\text{cos}\left(\frac{2\pi D}{\sigma }+\text{f}\right)e^{-D/\lambda_{s}}$$

where σ is the molecular diameter, λ*_S_* is the decay length, *D* is the distance between the walls, *ϕ* is the phase shift and *F*_o_ is the measured force amplitude. Richetti *et al.* [317] suggested a similar equation to describe the interaction between two surfaces across a smectic liquid, studied with AFM [318].

There is another short-range force that cannot be accounted for by the DLVO theory, which is not oscillatory but smoothly varying, *i.e*., monotonic. This force is exponentially repulsive and is commonly referred to as the hydration or structural force (or solvation force in fluids other than water) [319–322]. Because of the correlation with the low (or negative) energy of wetting of the solids with water, the repulsive force has been attributed to the energy required to remove the water of hydration from the surface, or the surface adsorbed species (secondary hydration), presumably because of strong charge-dipole, dipole-dipole or H-bonding interactions [323,324]. The concept of hydration force emerged to explain measurements of forces between neutral lipid bilayer membranes [324]. Repulsive hydration forces appear to arise whenever water molecules strongly bind to surfaces containing hydrophilic groups, *i.e*., certain ionic, zwitterionic, or H-bonding groups. Hydrogen bonding commonly serves as the mechanistic basis of structural forces. Generally, for a solvated surface, solvent molecules highly restricted in their motion experience structural forces. When the solvent is water, this orientation restriction is referred to as hydration pressure [279]. Israelachvili [23] further explains that this effect is not limited to a primary hydration shell, but rather propagates radially towards the bulk solution into a secondary hydration shell. Hydration forces are relatively short-ranged so that at salt concentrations below 0.1 M they can easily be distinguished from the longer range electrostatic and vdW forces. In contrast to the electrostatic double-layer force, hydration forces tend to become stronger and longer ranged with increasing salt concentration, especially for divalent cations [8]. A large hydration force could have important implications for AFM imaging, because to probe the true surface of a macromolecule, the probe would have to break through the hydration “shell” [325]. If the required force is too large, the structure below the “shell” could be deformed, resulting in a lower resolution.

The hydration pressure decays with the distance, and therefore the repulsive hydration may be represented by an empirical exponential function [326]:

(5.79) $$w=w_{o}e^{-D/\lambda_{H}}$$

where the decay length λ*_H_* is the range λ_H_ ≈ 0.6–1.1 nm for 1:1 electrolytes [327], *w*_o_ is the hydration force constant which depends on the hydration of the surfaces but it is usually below 3–30 mJ m^−2^, and *D* is the distance between the surfaces.

Hydration forces have been suggested as responsible for the short-range repulsion observed between silica surfaces [328,329]. Equations 5.80 and 5.81 were used to fit the experimental short-range forces (that exponentially decay on the separation distance) between different surfaces: silica, mica, montmorillonite and lipid bilayers [274,330,331]:

(5.80) $$F(x)=C_{H}\;\text{exp}\left(-\frac{D}{\lambda_{H}}\right)$$

(5.81) $$F(x)=C_{1}\;\text{exp}\left(-\frac{D}{\lambda_{H}}\right)+C_{2}\;\text{exp}\left(-\frac{D}{\lambda_{H}}\right)$$

where *F*(*x*) is the short-range force, *D* is the separation between the surfaces, and C_H_ is a hydration constant, assuming that the short-range force is due to hydration forces. Equations 5.80 and 5.81 are empirical relations, which are not supported by any theory [332]. Valle-Delgado *et al.* [333] utilized these equations to estimate interaction forces between bovine serum albumin (BSA) layers adsorbed on different substrates (silica and polystyrene), as a function of pH and salt concentration. They observed that electrostatic and steric forces dominate the interactions at low salt concentrations; in contrast, at high salt concentrations an attractive interaction was observed, which was explained with hydration forces obtained with Equation 5.80. The same authors used this relation to estimate hydration forces in the interaction between apoferritin (protein) molecules adsorbed on silica surfaces [334] and between silica surfaces [332].

**Figure 20:**
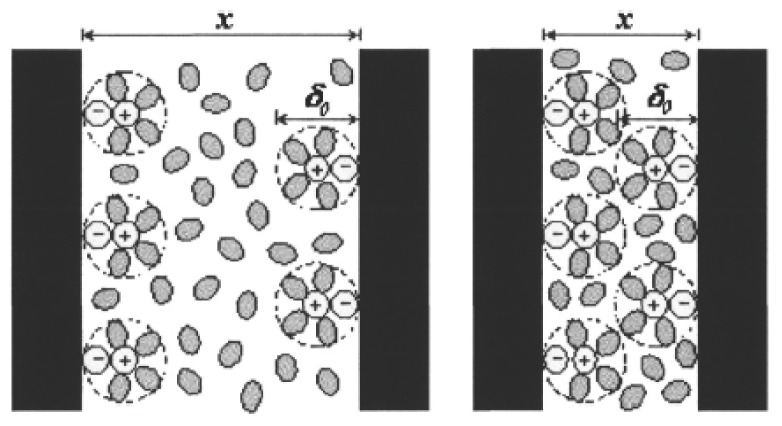
Schematic picture for the origin of hydration forces according to the model of Paunov *et al.* (Reproduced by permission of Elsevier [335]).

Paunov *et al.* [335] suggested that hydration forces in protein suspensions are due to the overlap of a layer of hydrated ions adsorbed on the surfaces. Figure 20 shows a schematic picture of this repulsive mechanism. The force between a plane and a sphere of radius R due to the overlap of the hydrated ions layers (Stern layers) is given, according to the model of Paunov *et al*. [335] by:

(5.82) $$F_{Hydration}(x)\approx R\left[-\frac{4\pi \delta_{o}kT}{\nu_{w}}\left\{\phi_{s}+\text{ln}\left(1-\phi_{s}\right)\right\}\left(\frac{2\delta_{o}}{x}-1\right)\right]$$

where υ_W_ is the volume occupied by a water molecule (υ_w_ ≈ 0.03 nm^3^), *ϕ*_s_ is the volume fraction of hydrated ions in the Stern layer, and δ_0_ is the diameter of the hydrated ions.

In addition to the equations above, other models have been proposed to explain the hydration repulsion:

1. Water Structure Theory: in the water-structuring models, the short-range repulsive interaction is attributed to an alignment of water dipoles in the vicinity of a hydrophilic surface, where the range of the surface force is determined by the orientation-correlation length of the solvent molecules [336]. Other researchers also suggested that the origin of the hydration force between silica surfaces may be related to the structuring of water molecules at the silica-water interface [337–339]. It is known that water can form strong H-bonds with the silanol groups. Derjaguin suggested that next to the silica surface there might be a layer of structured water up to 900 Å thick [340]. Attard and Batchelor [341] suggested that due to the strong orientation of water molecules near polar surfaces, there are fewer configurations available to maintain the bulk water structure and this represents lost entropy, which leads to a repulsive force.
2. Image-charge model: the image-charge models take into account the discreteness of the surface charges, which induce orientation in the adjacent water dipoles [342].
3. Dielectric-saturation model: this model assigns the hydration repulsion to a layer with lower dielectric constant, ɛ, in the vicinity of the interfaces [343]. Henderson and Lozadacassau [344] suggested that since the water molecules at the surface are strongly oriented, there should be a region of smaller dielectric constant at the solvent substrate interface when compared to the bulk.
4. Excluded-volume model: it takes into account the finite size of the ions, leading to a lower counterion concentration near a charged surface, and to a weaker Debye screening of the electrostatic field, which results in a stronger repulsion between two charged surfaces at short separations [345].
5. Gel-like layer model: the presence of a porous *gel-like layer* on silica was proposed by Lyklema [346] to explain the high surface charge and low potentials of the silica surface. Theoretical calculations to account for the observed charging characteristics of oxides have indicated that the gel layer maybe ~2–6 nm thick. Vigil *et al.* [347] used this explanation in the analysis of their experiments using silica surfaces and SFA.
6. Layer of co-ions model: this relatively simple model [348] assumes that at sufficiently small thicknesses all co-ions are pressed out of the film so that it contains only counterions dissociated from the ionized surface groups. Under such conditions, the screening of the electric field of the film surface weakens, which considerably enhances the electrostatic repulsion in comparison with that predicted by DLVO theory. Such reduced screening of the electric field could exist only in a narrow range of film thicknesses, which practically coincides with the range where hydration is observed.

Experimentally, the magnitude of the hydration force could only be inferred because of the presence of other longer range forces [28]. However, owing to the lack of control over the shape of the AFM tip, it is difficult to establish a direct connection between hydration force and tip geometry [325]. For example, it is not clear how far the hydration force extends laterally and whether the surface beyond the very end of the tip could contribute substantially to the hydration force. The existence of a short range (≤4 nm) repulsive pressure was observed in experiments on the swelling of clays [349], on the stabilization on foam films [312,348], proteins [332,334,335] and interactions between phospholipid bilayers [274]. Pashley and Israelachvili observed at electrolytes concentrations below 10^−4^ M a typical DLVO maximum; however, at electrolyte concentrations higher than 10^−3^ M they did not observe the expected DLVO maximum and primary minimum [350,351]. When Israelachvili and Pashley measured the force between two mica surfaces in electrolytes, they found, in addition to the electrostatic and vdW force, a short-range repulsive force at higher salt concentrations [352]. The more hydrated cations such as Mg^2+^ and Ca^2+^ gave stronger repulsive forces than the less hydrated monovalent ions such as K^+^ and Cs^+^. The authors concluded that the repulsive force was due to the work required to dehydrate the adsorbed ions on forcing the mica sheets together. Similar results were observed by Horn *et al.* [353], Butt [28] and Claesson *et al.* [354]. The magnitude of the hydration repulsion was found to decrease in the order Mg^+^ > Ca^+^ > Li^+^ ≈ Na^+^ > K^+^ > Cs^+^ > >> H_3_O^+^ [135,355]. As a consequence, the conventional electrostatic (double layer) repulsion was suppressed if the solution’s ionic strength was increased; in contrast, the hydration repulsion was detected at higher ionic strengths. Further experimental evidence of hydration forces can be found in coagulation studies of silica sols [356–359]; lecithin bilayers [360]; glass fibers [337]; glass sphere [30], silica [338,361] and conducting polymers [8]. Additional information can be found in review articles [29,98,324].

Initial studies on mica surfaces with adsorbed surfactant molecules pointed to an attractive force that exceeded the calculated vdW attraction between the bare substrates. This force is termed hydrophobic, whose existence was confirmed by Christenson and Claesson [362] and Rabinovich and Derjaguin [363], who showed that the force range could be greatly enhanced by increasing the hydrophobicity of the surface. Hydrophobic attractive forces (that act between solvated molecules and nonpolar interfaces) between hydrophobic macroscopic bodies in water have been measured for different systems. Hydrophobic effects roughly fall in two classes, namely those that are influenced by the addition of salt and those that are not [364]. The origin of the force appears to depend on the type of surface [365], but is still not completely understood. Several hypotheses have been proposed as follows [135,366–370]: (1) The hydrophobic force could originate from changes of the water structure in the thin layer between hydrophobic surfaces compared to the structure of bulk water; (2) it could be the capillary force due to cavitation in the vicinity of hydrophobic surfaces [371,372], (3) it could arise from hydrodynamic fluctuations at a hydrophobic surface/water interface; (4) it could arise from correlated dipole-dipole or dipole-charge interactions (electrostatic phenomena) [373–375]; (5) it may result from dipole interactions associated with the large domains of ordered hydrocarbon chains or (6) it may arise from capillary bridging of nanobubbles attached on hydrophobic surfaces [376,377]. Hypothesis (6) is probably the most important to cause very long-range interaction.

**Figure 21:**
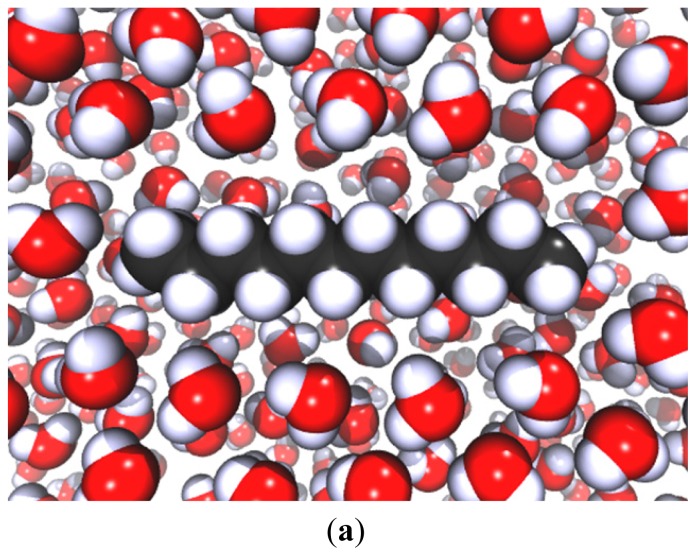

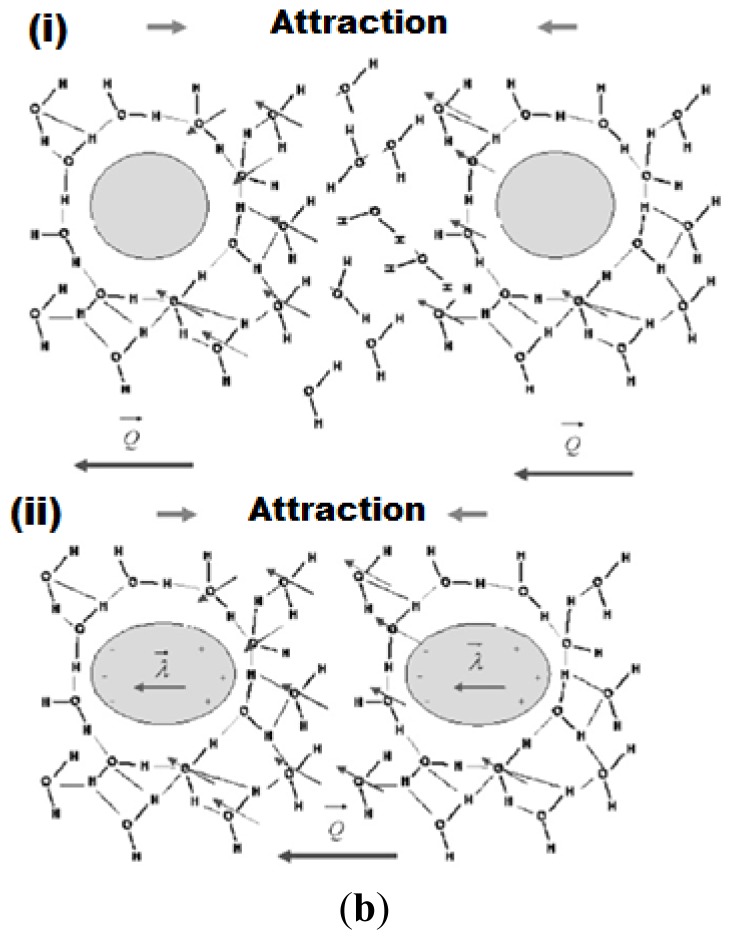
(**a**) Molecules or parts of molecules that have low or no affinity for water are called hydrophobic. These are usually composed of hydrocarbons that lack O or N or other polar groups and therefore cannot hydrogen bond or interact easily with water. The water molecules adjacent to hydrophobic domains form ice-like cages that surround the hydrophobic region [379]; (**b**) Schematic representation of the long-range attraction between hydrophobes initiated by the domains of polarized water (i) and by induced dipoles on the surface of the hydrophobic solutes (ii) (Reproduced by permission of Elsevier [380]).

A hydrophobic surface is one that is inert to water in the sense that it cannot bind to water molecules *via* ionic or hydrogen bonds. For surfaces having both the electron-donor and electron-acceptor values greater than that of water, the surface is termed hydrophobic. The orientation of water molecules in contact with other hydrophobic molecules is entropically unfavorable [378]. Therefore, such hydrophobic molecules will attract each other, since by coming together the entropically disfavor water is ejected into the bulk thereby reducing the total energy of the system [23,379]; see Figure 21a for further description. According to basic electrostatic principles, the domains of polarized water will establish long-range dipole-dipole interactions with each other. These interactions depend on the magnitude of the effective polarization fields *Q⃗* (see Figure 21b) [380]. Thus, the origin is in the polarization field produced by the strong correlation and coupling of the water molecules dipoles at the surfaces. This polarization field has been shown to give rise to dipoles on the surface of hydrophobic solutes that generate long-range hydrophobic attractions, which is crucial for colloidal interactions [224]. The hydrophobic force has resisted quantitative experimental determination as well as theoretical definition until relatively recently [226].

The hydrophobic force between two macroscopic surfaces was found to be of surprisingly long-range, decaying exponentially with a characteristic decay length λ_H_ = 1–2 nm, and then more gradually farther out [363,381,382]. The hydrophobic force can be far stronger than the vdW attraction, especially between hydrocarbon surfaces for which the Hamaker constant is quite small [279]. Therefore, for two surfaces in water, their purely hydrophobic interaction energy, *i.e.*, ignoring DLVO and oscillatory forces, in the range 0–10 nm is given by [383]:

(5.83) $$w=-2R\gamma_{i}e^{-D/\lambda_{H}}$$

where typically γ_i_ = 10–50 mJ m^−2^ [23].

The data are normally fitted by an empirical force law in the following form [384]:

(5.84) $$F=-R\left(\frac{\Gamma }{6D^{2}}\right)$$

where *R* is the tip radius, *D* is the tip-sample distance, and Γ is grafting density.

Rabinovich and Yoon [385] measured the hydrophobic force between a silica plate and a glass colloidal probe hydrophobized with octadecyltrichlorosilane (ODTCS). They obtained Γ by measuring the jump-to-contact of the curves, according to the following relationship:

(5.85) $$\Gamma_{jtc}=\frac{3k_{c}D_{jtc}^{3}}{R}$$

in which the subscript “*jtc*” means that this value is obtained with the “jump” method and *k_c_* is the elastic constant of the cantilever.

**Figure 22:**
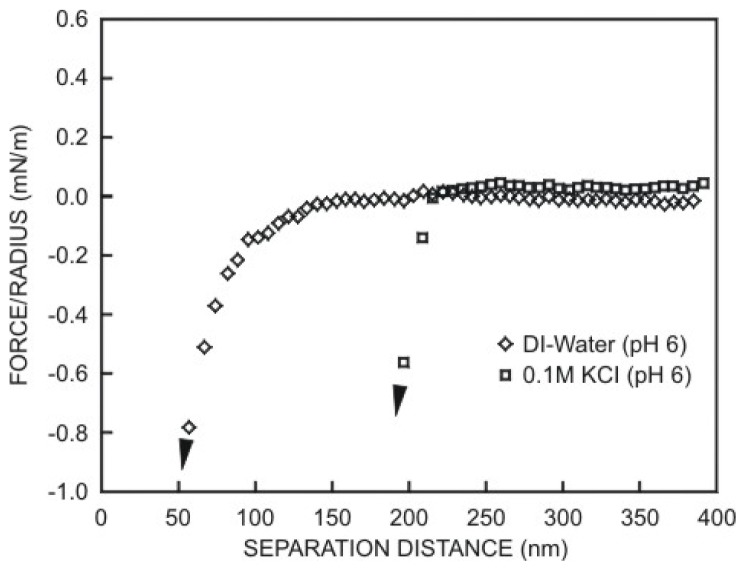
Interaction between hydrophobic surfaces (Reproduced by permission of Elsevier [387].

The effects from salt and chemical potential on adhesion between hydrophobic surfaces were investigated by Kokkoli and Zukoski [386], where they concluded that the adhesion is sensitive to surface roughness and lowering the solvent chemical potential produces an increase in the pull-off force. Freitas and Sharma [387] measured interactions between hydrophilic and hydrophobic surfaces in an aqueous medium at various pHs and ionic strengths as well as in some organic solvents using AFM. In hydrophilic systems the forces were well described by the DLVO theory at large separation distances. Long-range hydrophobic forces were not observed in hydrophilic-hydrophobic systems. However, the interaction between two hydrophobic surfaces (see Figure 22) was dominated by the long-range attraction due to hydrophobic forces [107]. Other experiments were carried out using AFM [388,389] and surface force apparatus (SFA) in the detection of hydrophobic forces [175,390].

## 6. Conclusions

This review was primarily aimed at theoretical models and direct measurements of vdW forces, particularly in the context of the use of atomic force spectroscopy (AFS). Because the molecular systems, for which vdW forces are so important, are affected by other interactions—especially H-bonding and electrostatic forces—we included in the review some discussion on other forces as well. This was important for understanding AFS measurements carried out in different media. In air, for instance, the formation of a thin water film and the capillary forces need to be addressed for a complete understanding of the whole system. For AFS measurements in liquid cells, on the other hand, the appearance of double-layer forces is essential, which is the reason why emphasis was placed on models for the double layers. All in all, we hope to have convinced the reader of the wide applicability of AFS, with potential impact in many areas of science and technology. The successful use of AFS however, requires identification and quantification of intermolecular forces, which is now becoming possible with the many physical models discussed in the review.

## Figures and Tables

**Table 1:** List of Hamaker constants in vacuum (air) and water for inorganic materials often used in experiments of force spectroscopy [23,87,93,98–100], as well as the Hamaker constants for some organic materials [101,102].

Material (1)	Material (2)	Medium (3)	Calculated × 10^−20^ J	Experimental × 10^−20^ J
Si_3_N_4_	Si_3_N_4_	Air 1	16.70	-
Si_3_N_4_	Si_3_N_4_	Water	4.80–5.90	6.10
Si_3_N_4_	Mica	Water	2.45	3.40
SiO_2_	SiO_2_	Air	6.50	-
SiO_2_	SiO_2_	Water	0.77–0.84	0.85–1.00
SiO_2_	Air	Water	−1.00	−1.00
SiO_2_	Mica	Water	1.20	1.20
SiO_2_	PTFE	Vacuum	16.44	13.70
Mica	Mica	Water	2.00–2.20	2.20
Mica	Mica	Air	9.86	-
Au	Au	Water	40.00	25.00
Silicon	Silicon	Air	18.65	-
Silicon	Silicon	Water	9.75	-
MgO	MgO	Air	12.10	-
MgO	MgO	Water	2.21	
Teflon	Teflon	Air	2.75	-
Teflon	Teflon	Water	0.33	
Polystyrene	Polystyrene	Air	6.58	-
Polystyrene	Polystyrene	Water	0.95	-
Poly(isoprene)	Poly(isoprene)	Air	5.99	-
Poly(isoprene)	Poly(isoprene)	Water	0.743	-
Ag	Ag	Air	20.00–49.00	38.50
C (diamondIIa)	C (diamondIIa)	Air	29.60	-
Cellulose	Cellulose	Air	5.80 ± 0.20	-
Cellulose	Cellulose	Water	0.80 ± 0.05	-
Hexadecane	Hexadecane	Air	5.20	-
Hexadecane	Hexadecane	Water	-	-
Cellulose	CaCO_3_	Air	7.40 ± 0.30	-
Cellulose	CaCO_3_	Water	0.57 ± 0.10	-
Cellulose	Si_3_N_4_	Air	9.50 ± 0.40	-
Cellulose	Si_3_N_4_	Water	0.80 ± 0.20	-
Cellulose	SiO_2_	Air	5.90 ± 0.30	-
Cellulose	SiO_2_	Water	0.35 ± 0.03	-
Cellulose	Mica	Air	7.20 ± 0.30	-
Cellulose	Mica	Water	0.43 ± 0.08	-
Cellulose	TiO_2_	Air	9.30 ± 0.40	-
Cellulose	TiO_2_	Water	1.20 ± 0.20	-
Octane	TPFP	Air	4.50	-
Octane	TPFP	Water	29.00	-
Octane	AF 2400	Air	4.00	-
Octane	AF 2400	Water	31.00	-
Octane	AF 1600	Air	4.10	-
Octane	AF 1600	Water	32.00	-
Octane	PTFE. LD	Air	4.60	-
Octane	PTFE. LD	Water	40.00	-
Octane	PTFE. HD	Air	5.10	-
Octane	PTFE. HD	Water	42.00	-
Octane	PDMS (liq)	Air	4.05	-
Octane	PDMS (liq)	Water	38.00	-
Octane	PDMS (s)	Air	4.50	-
Octane	PDMS (s)	Water	40.00	-
Octane	PE. LD	Air	4.90	-
Octane	PE. LD	Water	43.00	-
Octane	Rubber	Air	5.05	-
Octane	Rubber	Water	50.00	-
P-Xylene	TPFP	Air	3.40	-
P-Xylene	TPFP	Water	22.00	-
P-Xylene	AF 2400	Air	4.00	-
P-Xylene	AF 2400	Water	27.00	-
P-Xylene	AF 1600	Air	4.10	-
P-Xylene	AF 1600	Water	30.00	-
P-Xylene	PTFE. LD	Air	4.70	-
P-Xylene	PTFE. LD	Water	31.00	-
P-Xylene	PTFE. HD	Air	5.07	-
P-Xylene	PTFE. HD	Water	34.00	-
P-Xylene	PDMS (liq)	Air	4.10	-
P-Xylene	PDMS (liq)	Water	42.00	-
P-Xylene	PDMS (s)	Air	4.50	-
P-Xylene	PDMS (s)	Water	45.00	-
P-Xylene	PE. LD	Air	5.00	-
P-Xylene	PE. LD	Water	51.00	-
P-Xylene	Rubber	Air	5.30	-
P-Xylene	Rubber	Water	60.00	-
D-α-Pinene	TPFP	Air	4.00	-
D-α-Pinene	TPFP	Water	21.00	-
D-α-Pinene	AF 2400	Air	4.40	-
D-α-Pinene	AF 2400	Water	30.00	-
D-α-Pinene	AF 1600	Air	4.50	-
D-α-Pinene	AF 1600	Water	32.00	-
D-α-Pinene	PTFE. LD	Air	5.10	-
D-α-Pinene	PTFE. LD	Water	41.00	-
D-α-Pinene	PTFE. HD	Air	5.60	-
D-α-Pinene	PTFE. HD	Water	51.00	-
D-α-Pinene	PDMS (liq)	Air	4.50	-
D-α-Pinene	PDMS (liq)	Water	40.00	-
D-α-Pinene	PDMS (s)	Air	5.00	-
D-α-Pinene	PDMS (s)	Water	48.00	-
D-α-Pinene	PE. LD	Air	5.40	-
D-α-Pinene	PE. LD	Water	59.00	-
D-α-Pinene	Rubber	Air	5.60	-
D-α-Pinene	Rubber	Water	61.00	-
Olive oil	TPFP	Air	4.10	-
Olive oil	TPFP	Water	21.00	-
Olive oil	AF 2400	Air	4.50	-
Olive oil	AF 2400	Water	30.00	-
Olive oil	AF 1600	Air	4.80	-
Olive oil	AF 1600	Water	35.00	-
Olive oil	PTFE. LD	Air	5.40	-
Olive oil	PTFE. LD	Water	50.00	-
Olive oil	PTFE. HD	Air	5.95	-
Olive oil	PTFE. HD	Water	60.00	-
Olive oil	PDMS (liq)	Air	4.80	-
Olive oil	PDMS (liq)	Water	40.00	-
Olive oil	PDMS (s)	Air	5.00	-
Olive oil	PDMS (s)	Water	48.00	-
Olive oil	PE. LD	Air	5.60	-
Olive oil	PE. LD	Water	59.00	-
Olive oil	Rubber	Air	5.90	-
Olive oil	Rubber	Water	69.00	-
Sunfloweroil	TPFP	Air	4.00	-
Sunfloweroil	TPFP	Water	20.00	-
Sunfloweroil	AF 2400	Air	4.60	-
Sunfloweroil	AF 2400	Water	30.00	-
Sunfloweroil	AF 1600	Air	4.70	-
Sunfloweroil	AF 1600	Water	35.00	-
Sunfloweroil	PTFE. LD	Air	5.20	-
Sunfloweroil	PTFE. LD	Water	44.00	-
Sunfloweroil	PTFE. HD	Air	5.90	-
Sunfloweroil	PTFE. HD	Water	57.00	-
Sunfloweroil	PDMS (liq)	Air	4.80	-
Sunfloweroil	PDMS (liq)	Water	40.00	-
Sunfloweroil	PDMS (s)	Air	5.00	-
Sunfloweroil	PDMS (s)	Water	49.00	-
Sunfloweroil	PE. LD	Air	5.70	-
Sunfloweroil	PE. LD	Water	60.00	-
Sunfloweroil	Rubber	Air	5.90	-
Sunfloweroil	Rubber	Water	64.00	-
Tricosenoicacid	TPFP	Air	4.30	-
Tricosenoicacid	TPFP	Water	19.00	-
Tricosenoicacid	AF 2400	Air	4.90	-
Tricosenoicacid	AF 2400	Water	30.00	-
Tricosenoicacid	AF 1600	Air	5.00	-
Tricosenoicacid	AF 1600	Water	39.00	-
Tricosenoicacid	PTFE. LD	Air	5.70	-
Tricosenoicacid	PTFE. LD	Water	58.00	-
Tricosenoicacid	PTFE. HD	Air	6.20	-
Tricosenoicacid	PTFE. HD	Water	70.00	-
Tricosenoicacid	PDMS (liq)	Air	5.00	-
Tricosenoicacid	PDMS (liq)	Water	40.05	-
Tricosenoicacid	PDMS (s)	Air	5.40	-
Tricosenoicacid	PDMS (s)	Water	50.00	-
Tricosenoicacid	PE. LD	Air	5.90	-
Tricosenoicacid	PE. LD	Water	69.00	-
Tricosenoicacid	Rubber	Air	6.10	-
Tricosenoicacid	Rubber	Water	74.00	-
Cd-Arachidate	TPFP	Air	4.00	-
Cd-Arachidate	TPFP	Water	19.00	-
Cd-Arachidate	AF 2400	Air	4.30	-
Cd-Arachidate	AF 2400	Water	27.00	-
Cd-Arachidate	AF 1600	Air	4.30	-
Cd-Arachidate	AF 1600	Water	30.00	-
Cd-Arachidate	PTFE. LD	Air	5.08	-
Cd-Arachidate	PTFE. LD	Water	37.00	-
Cd-Arachidate	PTFE. HD	Air	5.50	-
Cd-Arachidate	PTFE. HD	Water	46.00	-
Cd-Arachidate	PDMS (liq)	Air	4.70	-
Cd-Arachidate	PDMS (liq)	Water	42.00	-
Cd-Arachidate	PDMS (s)	Air	5.00	-
Cd-Arachidate	PDMS (s)	Water	50.00	-
Cd-Arachidate	PE. LD	Air	5.40	-
Cd-Arachidate	PE. LD	Water	62.00	-
Cd-Arachidate	Rubber	Air	5.70	-
Cd-Arachidate	Rubber	Water	70.00	-
Cellulose	TPFP	Air	4.90	-
Cellulose	TPFP	Water	18.00	-
Cellulose	AF 2400	Air	5.20	-
Cellulose	AF 2400	Water	37.00	-
Cellulose	AF 1600	Air	5.50	-
Cellulose	AF 1600	Water	39.00	-
Cellulose	PTFE. LD	Air	6.20	-
Cellulose	PTFE. LD	Water	60.00	-
Cellulose	PTFE. HD	Air	6.90	-
Cellulose	PTFE. HD	Water	83.00	-
Cellulose	PDMS (liq)	Air	5.70	-
Cellulose	PDMS (liq)	Water	40.00	-
Cellulose	PDMS (s)	Air	6.00	-
Cellulose	PDMS (s)	Water	51.00	-
Cellulose	PE. LD	Air	6.50	-
Cellulose	PE. LD	Water	78.00	-
Cellulose	Rubber	Air	6.70	-
Cellulose	Rubber	Water	82.00	-
Hexadecane	TPFP	Air	4.00	-
Hexadecane	TPFP	Water	22.00	-
Hexadecane	AF 2400	Air	4.50	-
Hexadecane	AF 2400	Water	32.00	-
Hexadecane	AF 1600	Air	4.70	-
Hexadecane	AF 1600	Water	34.00	-
Hexadecane	PTFE. LD	Air	5.10	-
Hexadecane	PTFE. LD	Water	42.00	-
Hexadecane	PTFE. HD	Air	5.60	-
Hexadecane	PTFE. HD	Water	55.00	-
Hexadecane	PDMS (liq)	Air	4.50	-
Hexadecane	PDMS (liq)	Water	38.00	-
Hexadecane	PDMS (s)	Air	5.00	-
Hexadecane	PDMS (s)	Water	31.00	-
Hexadecane	PE. LD	Air	5.10	-
Hexadecane	PE. LD	Water	53.00	-
Hexadecane	Rubber	Air	5.30	-
Hexadecane	Rubber	Water	59.00	-
PVA	TPFP	Air	4.60	-
PVA	TPFP	Water	18.00	-
PVA	AF 2400	Air	5.10	-
PVA	AF 2400	Water	33.00	-
PVA	AF 1600	Air	5.40	-
PVA	AF 1600	Water	40.00	-
PVA	PTFE. LD	Air	6.08	-
PVA	PTFE. LD	Water	60.00	-
PVA	PTFE. HD	Air	6.40	-
PVA	PTFE. HD	Water	78.00	-
PVA	PDMS (liq)	Air	5.50	-
PVA	PDMS (liq)	Water	40.05	-
PVA	PDMS (s)	Air	5.70	-
PVA	PDMS (s)	Water	71.00	-
PVA	PE. LD	Air	6.10	-
PVA	PE. LD	Water	77.00	-
PVA	Rubber	Air	6.30	-
PVA	Rubber	Water	80.00	-
BSA	TPFP	Air	5.00	-
BSA	TPFP	Water	16.00	-
BSA	AF 2400	Air	5.50	-
BSA	AF 2400	Water	29.00	-
BSA	AF 1600	Air	5.80	-
BSA	AF 1600	Water	40.00	-
BSA	PTFE. LD	Air	6.40	-
BSA	PTFE. LD	Water	61.00	-
BSA	PTFE. HD	Air	7.00	-
BSA	PTFE. HD	Water	80.05	-
BSA	PDMS (liq)	Air	5.60	-
BSA	PDMS (liq)	Water	42.00	-
BSA	PDMS (s)	Air	6.00	-
BSA	PDMS (s)	Water	60.00	-
BSA	PE. LD	Air	6.50	-
BSA	PE. LD	Water	80.00	-
BSA	Rubber	Air	7.00	-
BSA	Rubber	Water	82.00	-
Nylon 6	TPFP	Air	4.50	-
Nylon 6	TPFP	Water	17.00	-
Nylon 6	AF 2400	Air	5.00	-
Nylon 6	AF 2400	Water	23.00	-
Nylon 6	AF 1600	Air	5.10	-
Nylon 6	AF 1600	Water	37.00	-
Nylon 6	PTFE. LD	Air	5.80	-
Nylon 6	PTFE. LD	Water	50.00	-
Nylon 6	PTFE. HD	Air	6.20	-
Nylon 6	PTFE. HD	Water	64.00	-
Nylon 6	PDMS (liq)	Air	5.20	-
Nylon 6	PDMS (liq)	Water	40.00	-
Nylon 6	PDMS (s)	Air	5.40	-
Nylon 6	PDMS (s)	Water	53.00	-
Nylon 6	PE. LD	Air	6.08	-
Nylon 6	PE. LD	Water	79.00	-
Nylon 6	Rubber	Air	6.30	
Nylon 6	Rubber	Water	80.00	-
Silver	Silver	Vacuum	20.30	38.20
Silver	Silver	Nitrogen	20.30	37.90
Copper	Copper	Vacuum	24.82	27.20
Copper	Copper	Nitrogen	24.82	27.10
PTFE	PTFE	Vacuum	3.63	-
PTFE	PTFE	Nitrogen	3.63	-
Silicon Dioxide	Silicon Dioxide	Vacuum	6.55	-
Silicon Dioxide	Silicon Dioxide	Nitrogen	6.55	7.20
Titanium Nitride	Titanium Nitride	Vacuum	15.73	-
Titanium Nitride	Titanium Nitride	Nitrogen	15.73	-
Parylene-n	Parylene-n	Vacuum	11.10	-
Parylene-n	Parylene-n	Nitrogen	11.10	-
Silver	Copper	Vacuum	22.45	32.60
Silver	Copper	Nitrogen	22.45	32.40
Silver	Silicon Dioxide	Vacuum	11.12	12.92
Silver	Silicon Dioxide	Nitrogen	11.12	12.70
Silver	PTFE	Vacuum	8.34	13.70
Silver	PTFE	Nitrogen	8.34	13.60
Silver	Parylene-*n*	Vacuum	14.30	11.80
Silver	Parylene-*n*	Nitrogen	14.30	11.60
Silver	Cross linked Parylene-*n*	Vacuum	14.30	12.10
Silver	Cross linked Parylene-*n*	Nitrogen	14.30	12.00
Silver	Titanium Nitride	Vacuum	16.80	16.40
Silver	Titanium Nitride	Nitrogen	16.80	16.10
Copper	Silicon Dioxide	Vacuum	11.60	14.10
Copper	Silicon Dioxide	Nitrogen	11.60	13.90
Copper	PTFE	Vacuum	8.72	13.10
Copper	PTFE	Nitrogen	8.72	12.80
Copper	Parylene-*n*	Vacuum	15.00	9.80
Copper	Parylene-*n*	Nitrogen	15.00	10.10
Copper	Cross linked Parylene-*n*	Vacuum	15.00	11.00
Copper	Cross linked Parylene-*n*	Nitrogen	15.00	11.10
Copper	Titanium Nitride	Vacuum	17.59	12.30
Copper	Titanium Nitride	Nitrogen	17.59	12.50
Silicon Dioxide	PTFE	Vacuum	4.87	-
Silicon Dioxide	PTFE	Nitrogen	4.87	7.60
Silicon Dioxide	Parylene-*n*	Vacuum	8.55	-
Silicon Dioxide	Parylene-*n*	Nitrogen	8.55	6.80
Silicon Dioxide	Cross linked Parylene-*n*	Vacuum	8.55	-
Silicon Dioxide	Cross linked Parylene-*n*	Nitrogen	8.55	6.90
Silicon Dioxide	Titanium Nitride	Vacuum	10.10	-
Silicon Dioxide	Titanium Nitride	Nitrogen	10.10	8.80

1 Hamaker constants for water (*j*) and air (vacuum) (*k*) interacting across air (vacuum) are 3.7 and 0, respectively.

**Table 2:** Forces in AFM measurements, where the type of force is given in the first column, that also specifies whether the interaction is more common in air (a), vacuum (v) or solution (s). Special features of each of the forces are mentioned in the 3rd column, while the second column provides either the expression for the force or the Section in this paper in which further information can be found [43,107,108].

Pull-on force		
Types of force	Topics or equations	Special features
van der Waals (a, v & s)	See Section2	Ubiquitous force both in vacuum and in liquids, existing between all bodies.
Hydration (s)	See Section5 (Topic C)	Hydration repulsive force attributed to the energy required to remove the water of hydration from the surface, or the surface adsorbed species.
Solvation (s)	See Section5 (Topic C)	Solvation forces arise whenever liquid molecules are compelled to order in almost discrete layers between two surfaces.
Double-Layer (s)	See Section 5 (Topic C)	A force that exists only between charged molecules (ions) or surfaces, and depends on the electrolyte concentration.
Elastic (a, v & s)	$F_{els}=\frac{4E\sqrt{R_{t}}}{3\left(1-\nu^{2}\right)}\delta^{3/2}$	Relation between the applied forces to the depth of indentation as the tip is pushed against the sample.
Brush (a, v & s)	$F_{b}=\frac{50Lk_{B}T}{d^{3}}e^{-2\pi D/L}$	Polymer-brushing forces that result from the thermally driven motion of polymers grafted onto a solid surface in solution.
**Pull-off force**		
Adhesion (a, v & s)	See Section 5 (Topic A)	Adhesion between a sphere and a plane in the absence of contaminating adsorbates (typically in a vacuum).
Capillary (a)	See Section 5 (Topic B)	Capillary adhesion–very common under ambient conditions, under which many surfaces have a thin water layer.
Hydrophobic (s)	See Section 5 (Topic C)	The hydrophobic force has different origins depending on the system. For example, when two hydrophobic surfaces are in contact, a vapor cavity bridging is formed to cause strong adhesion.
Polymer Extension (a, v & s)	$F_{pol}=\frac{k_{B}T}{\varsigma }L*\left(\frac{x}{N\varsigma }\right)$	Polymers break or detach from one of the surfaces (tip and sample).
Specific Binding (a, v & s)	$F_{B}=\frac{U-kT\;\text{ln}\left(\tau /\tau_{o}\right)}{\Lambda }$	Specific interactions (chemical force microscopy). Antibody-antigen interactions, receptor-ligand interactions and complementary binding.

Note: (v), (s) and (a) apply to interactions in vacuum, solution and air, respectively. Definitions: E: Elastic modulus, *R_t_*: Radius of probe sphere (tip), ν: Poisson ratio, δ: Indentation depth, L: Brush thickness in a good solvent, D: Probe-sample distance, U: Bond energy, τ: Period over which the bond will rupture, τo: Reciprocal of the natural bond frequency, ζ: Monomer length, L*: Inverse Langevin function, x : Elongation of a polymer, *N*: Number of units in a polymer, d: mean distance between polymers.

**Table 3:** vdW interaction potential (*w*(*r*)) and force (*F* = −d*w*/d*r*) between macroscopic bodies, for the most common geometries. *R* is the radius of the spheres or cylinders, *D* is the distance between the interacting bodies, and *A* is the Hamaker constant [23,43]. A negative force F implies attraction (*A positive*), a positive force means repulsion (*A negative*).

Geometry (*D* << R)	vdW Interactions	

	Force	Energy
Two atoms or small molecules	$F(r)=-\frac{6C_{vdW}}{r^{7}}$	$w(r)=-\frac{C_{vdW}}{r^{6}}$
Atom-surface	$F(D)=-\frac{3\pi C\rho }{6D^{4}}$	$w(D)=-\frac{\pi C\rho }{6D^{3}}$
Two spheres	$F(D)=-\frac{A}{6D^{2}}\left(\frac{R_{1}R_{2}}{R_{1}+R_{2}}\right)$	$w(D)=-\frac{A}{6D}\left(\frac{R_{1}R_{2}}{R_{1}+R_{2}}\right)$
Sphere-flat surface	$F(D)=-\frac{AR}{6D^{2}}$	$w(D)=-\frac{AR}{6D}$
Two flat plates	$F(D)=-\frac{A}{6\pi D^{3}}$	$w(D)=-\frac{A}{12\pi D^{2}}$
Two cylinders or fi1aments crossed at 90°	$F(D)=\frac{-A\sqrt{R_{1}R_{2}}}{6D^{2}}$	$w(D)=\frac{-A\sqrt{R_{1}R_{2}}}{6D}$
Cylinder near a flat surface	$F(D)=-\frac{-A\sqrt{R}}{8\sqrt{2}D^{\frac{5}{2}}}$	$w(D)=-\frac{-A\sqrt{R}}{12\sqrt{2}D^{\frac{3}{2}}}$
Two parallel cylinders or rods	$F(D)=\frac{-A}{8\sqrt{2}D^{\frac{5}{2}}}{\left(\frac{R_{1}R_{2}}{R_{1}+R_{2}}\right)}^{1/2}$	$w(D)=\frac{-A}{12\sqrt{2}D^{\frac{3}{2}}}{\left(\frac{R_{1}R_{2}}{R_{1}+R_{2}}\right)}^{1/2}$

**Table 4:** Experimental results for several samples in vacuum [154].

Sample	Surface Energy γ_lit_ (mJ/m^2^)	Tip radius (μm)	Attractive force (nN)
Mica	300, 375	2.5 ± 0.5	230 ± 30
Graphite	96, 123	2.5 ± 0.5	140 ± 90
Al_2_O_3_	45	2.0 ± 0.5	85 ± 25
CH_3_(CH_2_)_16_COOH	21	2.0 ± 0.5	17 ± 11
CF_3_(CH_2_)_16_COOH	20	3.0 ± 0.5	5.0 ± 4.0
PTFE	18	2.5 ± 0.5	2.0 ± 2.0

## References

[1] Burnham N.A., Kulik A.J., Bhushan B. (1999). Surface Forces and Adhesion. Handbook of Micro/Nanotribology.

[2] Myers D (1999). Surfaces, Interfaces, and Colloids: Principles and Applications.

[3] Varandas A.J.C., Brandao J. (1982). A simple semi-empirical approach to the intermolecular potential of vanderwaals systems.1. Isotropic interactions—Application to the lowest triplet-state of the alkali dimers. Mol. Phys.

[4] Van der Waals J.D. (1893). Thermodynamische Theorie der Capillariteit in de Onderstelling van Continue Dichtheidsverandering.

[5] Kitchener J.A., Prosser A.P. (1957). Direct measurement of the long-range van der waals forces. Proc. R. Soc. A.

[6] Drelich J., Mittal K.L. (2005). Atomic Force Microscopy in Adhesion Studies.

[7] Sun L., Li X., Hede T., Tu Y.Q., Leck C., Agren H. (2012). Molecular dynamics simulations of the surface tension and structure of salt solutions and clusters. J. Phys. Chem. B.

[8] Leite F.L., Borato C.E., da Silva W.T.L., Herrmann P.S.P., Oliveira O.N., Mattoso L.H.C. (2007). Atomic force spectroscopy on poly(*O*-ethoxyaniline) nanostructured films: Sensing nonspecific interactions. Microsc. Microanal..

[9] Bruch L.W.Evaluation of the van der waals force for atomic force microscopyPhys. Rev. B 2005 72 10.1103/PhysRevB.72.033410

[10] Nicolosi V., Nellist P.D., Sanvito S., Cosgriff E.C., Krishnamurthy S., Blau W.J., Green M.L.H., Vengust D., Dvorsek D., Mihailovic D. (2007). Observation of van der waals driven self-assembly of mosi nanowires into a low-symmetry structure using aberration-corrected electron microscopy. Adv. Mater.

[11] French R.H., Parsegian V.A., Podgornik R., Rajter R.F., Jagota A., Luo J., Asthagiri D., Chaudhury M.K., Chiang Y.M., Granick S. (2010). Long range interactions in nanoscale science. Rev. Mod. Phys.

[12] Leite F.L., Neto Mde O., Paterno L.G., Ballestero M.R.M., Polikarpov I., Mascarenhas Y.P., Herrmann P.S.P., Mattoso L.H.C., Oliveira O.N. (2007). Nanoscale conformational ordering in polyanilines investigated by saxs and afm. J. Colloid Interface Sci..

[13] Xu L., Lio A., Hu J., Ogletree D.F., Salmeron M. (1998). Wetting and capillary phenomena of water on mica. J. Phys. Chem. B.

[14] Lubarsky G.V., Mitchell S.A., Davidson M.R., Bradley R.H. (2006). Van der waals interaction in systems involving oxidised polystyrene surfaces. Colloids Surf. A: Physicochem. Eng. Asp.

[15] Wang C.X., Chang S., Gong X.Q., Yang F., Li C.H., Chen W.Z. (2012). Progress in the scoring functions of protein-protein docking. Acta Phys.Chim. Sin.

[16] Chen J.Z., Zhang D.L., Zhang Y.X., Li G.H. (2012). Computational studies of difference in binding modes of peptide and non-peptide inhibitors to mdm2/mdmx based on molecular dynamics simulations. Int. J. Mol. Sci.

[17] Elbaum M., Lipson S.G. (1994). How does a thin wetted film dry up?. Phys. Rev. Lett..

[18] Elbaum M., Schick M (1991). Application of the theory of dispersion forces to the surface melting of ice. Phys. Rev. Lett..

[19] Hodges C.S. (2002). Measuring forces with the AFM: Polymeric surfaces in liquids. Adv. Colloid Interface Sci.

[20] Ninham B.W., Parsegian V.A. (1970). Van der waals forces across triple-layer films. J. Chem. Phys.

[21] Israelachvili J.N. (1992). Adhesion forces between surfaces in liquids and condensable vapours. Surf. Sci. Rep.

[22] Israelachvili J.N., Adams G.E. (1978). Measurement of forces between two mica surfaces in aqueous electrolyte solutions in the range 0–100 nm. J. Chem. Soc., Faraday Trans.

[23] Israelachvili J.N. (1995). Intermolecular and Surface Forces.

[24] Binnig G., Quate C.F., Gerber C. (1986). Atomic force microscope. Phys. Rev. Lett.

[25] Hutter J.L., Bechhoefer J. (1994). Measurement and manipulation of van-der-waals forces in atomic-force microscopy. J. Vac. Sci. Technol. B.

[26] Meyer E. (1992). Atomic force microscopy. Prog. Surf. Sci.

[27] Frommer J.E. (1996). Scanning probe microscopy of organics, an update. Thin Solid Films.

[28] Butt H.J. (1991). Measuring electrostatic, vanderwaals, and hydration forces in electrolyte-solutions with an atomic force microscope. Biophys. J.

[29] Leite F.L., Herrmann P.S.P. (2005). Application of atomic force spectroscopy (afs) to studies of adhesion phenomena: A review. J. Adhes. Sci. Technol.

[30] Ducker W.A., Senden T.J., Pashley R.M. (1991). Direct measurement of colloidal forces using an atomic force microscope. Nature.

[31] Teschke O., Ceotto G., de Souza E.F. (2001). Rupture force of adsorbed self-assembled surfactant layers—Effect of the dielectric exchange force. Chem. Phys. Lett.

[32] Borkovec M., Papastavrou G. (2008). Interactions between solid surfaces with adsorbed polyelectrolytes of opposite charge. Curr. Opin. Colloid Interface Sci.

[33] Leite F.L., Paterno L.G., Borato C.E., Herrmann P.S.P., Oliveira O.N., Mattoso L.H.C. (2005). Study on the adsorption of poly(*O*-ethoxyaniline) nanostructured films using atomic force microscopy. Polymer.

[34] Sharp T.G., Oden P.I., Buseck P.R. (1993). Lattice-scale imaging of mica and clay (001) surfaces by atomic force microscopy using net attractive forces. Surf. Sci.

[35] Sasaki N., Tsukada M. (1999). Theory for the effect of the tip-surface interaction potential on atomic resolution in forced vibration system of noncontact afm. Appl. Surf. Sci.

[36] Erts D., Lohmus A., Lohmus R., Olin H., Pokropivny A.V., Ryen L., Svensson K. (2002). Force interactions and adhesion of gold contacts using a combined atomic force microscope and transmission electron microscope. Appl. Surf. Sci.

[37] Persson B.N.J. (1987). The atomic force microscope—Can it be used to study biological molecules. Chem. Phys. Lett.

[38] Dagastine R.R., White L.R. (2002). Forces between a rigid probe particle and a liquid interface—ii. The general case. J. Colloid Interface Sci.

[39] Sokolov I.Y., Henderson G.S., Wicks F.J. (1997). The contrast mechanism for true atomic resolution by afm in non-contact mode: Quasi-non-contact mode?. Surf. Sci.

[40] Stone A.J. (1996). The Theory of Intermolecular Forces.

[41] Bhushan B., Israelachvili J.N., Landman U. (1995). Nanotribology—Friction, wear and lubrication at the atomic-scale. Nature.

[42] Kim D.I., Grobelny J., Pradeep N., Cook R.F. (2008). Origin of adhesion in humid air. Langmuir.

[43] Leckband D., Israelachvili J. (2001). Intermolecular forces in biology. Q. Rev. Biophys.

[44] Van der Waals J.D. (1893). Thermodynamische theorie der capillariteit in de onderstelling van continue dichtheidsverandering.

[45] French R.H. (2000). Origins and applications of london dispersion forces and hamaker constants in ceramics. J. Am. Ceram. Soc.

[46] Garbassi F., Morra M., Occhiello E (1994). Polymer Surfaces from Physics to Technology.

[47] Tabor D., Winterto R.H. (1969). Direct measurement of normal and retarded van der waals forces. Proc. R. Soc. Lond. Ser. A: Math. Phys. Sci.

[48] Keesom W.H. (1915). The second virial coefficient for rigid spherical molecules whose mutual attraction is equivalent to that of a quadruplet placed at its center. Proc. R. Acad. Sci.

[49] Gerschel A. (1987). Dipole-induced static and dynamic liquid structures. J. Chem. Soc., Faraday Trans. 2 Mol. Chem. Phys.

[50] Debye P. (1920). Die van der waalsschen kohäsionskräfte. Physikalische Zeitschrift.

[51] London F. (1930). Zur theorie und systematik der molekularkräfte. Zeitschrift für Physik A Hadrons Nuclei.

[52] Feiler A.A., Bergstrom L., Rutland M.W. (2008). Superlubricity using repulsive van der waals forces. Langmuir.

[53] Duran-Vidal S., Simonin J.P., Turq P (2002). Electrolytes at Interfaces.

[54] Debye P. (1921). Molekularkräfte und ihre elektrische deutung. Physikalische Zeitschrift.

[55] Keesom W.M. (1921). Van der waals attractive force. Phys. Z.

[56] Keesom W.M. (1920). The quadrupole moments of the oxygen and nitrogen molecules. Proc. K. Ned. Akad. Wet.

[57] London F. (1930). Properties and applications of molecular forces. Zeitschrift für Physikalische Chemie (B).

[58] Jiang X.P., Toigo F., Cole M.W. (1984). The dispersion force of physical adsorption.1. Local theory. Surf. Sci.

[59] Jiang X.P., Toigo F., Cole M.W. (1984). The dispersion force of physical adsorption.2. Nonlocal theory. Surf. Sci.

[60] Hutson J.M., Fowler P.W., Zaremba E. (1986). Quadrupolar contributions to the atom-surface vanderwaals interaction. Surf. Sci.

[61] Blaney B.L., Ewing G.E. (1976). Vanderwaals molecules. Annu. Rev. Phys. Chem.

[62] Ewing G.E. (1976). Spectroscopy of vanderwaals molecules. Can. J. Phys.

[63] Watanabe A., Welsh H.L. (1964). Direct spectroscopic evidence of bound states of (h2)2 complexes at low temperatures. Phys. Rev. Lett.

[64] Henderso G, Ewing G.E. (1974). Infrared-spectrum, structure and properties of n2-ar van der waals molecule. Mol. Phys..

[65] Power E.A., Thirunamachandran T. (1996). Dispersion interactions between atoms involving electric quadrupole polarizabilities. Phys. Rev. A.

[66] Marinescu M., Babb J.F., Dalgarno A. (1994). Long-range potentials, including retardation, for the interaction of 2 alkali-metal atoms. Phys. Rev. A.

[67] Au C.K.E., Feinberg G. (1972). Higher-multipole contributions to retarded vanderwaals potential. Phys. Rev. A.

[68] Mayer J.E. (1933). Dispersion and polarizability and the van der waals potential in the alkali halides. J. Chem. Phys.

[69] Margenau H. (1931). The role of quadrupole forces in van der waals attractions. Phys. Rev.

[70] Fontana P.R., Bernstein R.B. (1964). Dipole-quadrupole + retardation effects in low-energy atom-atom scattering. J. Chem. Phys.

[71] Jain J.K., Shanker J., Khandelwal D.P. (1976). Evaluation of vanderwaals dipole-dipole and dipole-quadrupole energies in alkali-halides. Phys. Rev. B.

[72] Porsev S.G., Derevianko A. (2006). High-accuracy calculations of dipole, quadrupole, and octupole electric dynamic polarizabilities and van der waals coefficients c-6, c-8, and c-10 for alkaline-earth dimers. J. Exp. Theor. Phys.

[73] Chang T.Y. (1967). Moderately long-range interatomic forces. Rev. Mod. Phys.

[74] Pauling L., Beach J.Y. (1935). The van der waals interaction of hydrogen atoms. Phys. Rev.

[75] McLachlan A.D. (1963). Retarded dispersion forces in dielectrics at finite temperatures. Proc. R. Soc. Lond. Ser. A: Math. Phys. Sci.

[76] Landau L.D., Lifshitz E.M. Electrodynamics of Continuous Media.

[77] Laven J., Vissers J.P.C. (1999). The hamaker and the lifshitz approaches for the van der waals interaction between particles of composite materials dispersed in a medium. Colloids Surf. A: Physicochem. Eng. Asp.

[78] Hamaker H.C. (1937). The london—Van der waals attraction between spherical particles. Physica.

[79] Lifshitz E.M. (1956). The theory of molecular attractive forces between solids. Soviet Phys. JETP.

[80] Shluger A.L., Livshits A.I., Foster A.S., Catlow C.R.A. (2000). Theoretical modelling of non-contact atomic force microscopy on insulators. J. Phys-Condens. Mat.

[81] Leite F.L., Riul A., Herrmann P.S.P. (2003). Mapping of adhesion forces on soil minerals in air and water by atomic force spectroscopy (afs). J. Adhes. Sci. Technol.

[82] Wang H.Y., Hu M., Liu N., Xia M.F., Ke F.J., Bai Y.L. (2007). Multi-scale analysis of afm tip and surface interactions. Chem. Eng. Sci.

[83] Bowen W.R., Jenner F. (1995). The calculation of dispersion forces for engineering applications. Adv. Colloid Interface Sci.

[84] Sokolov I.Y. (2003). Pseudo-non-contact mode: Why it can give true atomic resolution. Appl. Surf. Sci.

[85] London F. (1937). The general theory of molecular forces. Trans. Faraday Soc.

[86] Ackler H.D., French R.H., Chiang Y.M. (1996). Comparisons of hamaker constants for ceramic systems with intervening vacuum or water: From force laws and physical properties. J. Colloid Interface Sci.

[87] Hough D.B., White L.R. (1980). The calculation of hamaker constants from lifshitz theory with applications to wetting phenomena. Adv. Colloid Interface Sci.

[88] Derjaguin B.V. (1954). A theory of the heterocoagulation, interaction and adhesion of dissimilar particles in solutions of electrolytes. Discuss. Faraday Soc.

[89] Visser J. (1972). On hamaker constants: A comparison between hamaker constants and lifshitz-van der waals constants. Adv. Colloid Interface Sci.

[90] Fowkes F.M. (1967). Surfaces and Interfaces.

[91] Van Oss C.J., Omenyi S.N., Neumann A.W. (1979). Negative hamaker coefficients. Ii. Phase separation of polymer solutions. Colloid Polym. Sci.

[92] Marra J. (1985). Controlled deposition of lipid monolayers and bilayers onto mica and direct force measurements between galactolipid bilayers in aqueous-solutions. J. Colloid Interface Sci.

[93] Cappella B., Dietler G. (1999). Force-distance curves by atomic force microscopy. Surf. Sci. Rep.

[94] Burnham N.A., Colton R.J., Pollock H.M. (1993). Interpretation of force curves in force microscopy. Nanotechnology.

[95] Meyer E., Heinzelmann H., Grutter P., Jung T., Hidber H.R., Rudin H., Guntherodt H.J. (1989). Atomic force microscopy for the study of tribology and adhesion. Thin Solid Films.

[96] Götzinger M., Peukert W. (2003). Disperse forces of particle –surface interactions: Direct afm measurements and modeling. Powder Technol.

[97] Das S., Sreeram P.A., Raychaudhuri A.K. (2007). A method to quantitatively evaluate the hamaker constant using the jump-into-contact effect in atomic force microscopy. Nanotechnology.

[98] Butt H.J., Cappella B., Kappl M. (2005). Force measurements with the atomic force microscope: Technique, interpretation and applications. Surf. Sci. Rep.

[99] Bergstrom L. (1997). Hamaker constants of inorganic materials. Adv. Colloid Interface Sci.

[100] Eichenlaub S., Chan C., Beaudoin S.P. (2002). Hamaker constants in integrated circuit metalization. J. Colloid Interface Sci.

[101] Bergstrom L., Stemme S., Dahlfors T., Arwin H., Odberg L. (1999). Spectroscopic ellipsometry characterisation and estimation of the hamaker constant of cellulose. Cellulose.

[102] Drummond C.J., Chan D.Y.C. (1996). Theoretical analysis of the soiling of “nonstick” organic materials. Langmuir.

[103] Froberg J.C., Rojas O.J., Claesson P.M. (1999). Surface forces and measuring techniques. Int. J. Miner. Process.

[104] Gu Y.A. (2001). Experimental determination of the hamaker constants for solid-water-oil-systems. J. Adhes. Sci. Technol.

[105] Cooper K., Gupta A., Beaudoin S. (2000). Substrate morphology and particle adhesion in reacting systems. J. Colloid Interface Sci.

[106] Hoh J.H., Engel A. (1993). Friction effects on force measurements with an atomic-force microscope. Langmuir.

[107] Reich Z., Kapon R., Nevo R., Pilpel Y., Zmora S., Scolnik Y. (2001). Scanning force microscopy in the applied biological sciences. Biotechnol. Adv.

[108] Heinz W.F., Hoh J.H. (1999). Spatially resolved force spectroscopy of biological surfaces using the atomic force microscope. Trends Biotechnol.

[109] Cleveland J.P., Manne S., Bocek D., Hansma P.K. (1993). A nondestructive method for determining the spring constant of cantilevers for scanning force microscopy. Rev. Sci. Instrum.

[110] Sader J.E., Larson I., Mulvaney P., White L.R. (1995). Method for the calibration of atomic-force microscope cantilevers. Rev. Sci. Instrum.

[111] Gibson C.T., Watson G.S., Myhra S. (1996). Determination of the spring constants of probes for force microscopy/spectroscopy. Nanotechnology.

[112] Sader J.E. (1998). Frequency response of cantilever beams immersed in viscous fluids with applications to the atomic force microscope. J. Appl. Phys.

[113] Sader J.E. (1995). Parallel beam approximation for v-shaped atomic-force microscope cantilevers. Rev. Sci. Instrum.

[114] Hutter J.L., Bechhoefer J. (1993). Calibration of atomic-force microscope tips. Rev. Sci. Instrum.

[115] Levy R., Maaloum M. (2002). Measuring the spring constant of atomic force microscope cantilevers: Thermal fluctuations and other methods. Nanotechnology.

[116] Burnham N.A., Colton R.J. (1989). Measuring the nanomechanical properties and surface forces of materials using an atomic force microscope. J. Vac. Sci. Technol. A: Vac. Surf. Films.

[117] Oliver W.C., Pharr G.M. (1992). An improved technique for determining hardness and elastic-modulus using load and displacement sensing indentation experiments. J. Mater. Res..

[118] Baselt D.R., Baldeschwieler J.D. (1994). Imaging spectroscopy with the atomico force microscope. J. Appl. Phys.

[119] Wang D., Fujinami S., Liu H., Nakajima K., Nishi T. (2010). Investingation of true surface morphology and nanomechanical properties of poly(styrene-*b*-ethilene-*co*-butylene-*b*-styrene) using nanomechanical mapping: Effects of composition. Macromolecules.

[120] Willing G.A., Ibrahim T.H., Etzler F.M., Neuman R.D. (2000). New approach to the study of particle-surface adhesion using atomic force microscopy. J. Colloid Interface Sci.

[121] Zuo F., Angelopoulos M., Macdiarmid A.G., Epstein A.J. (1987). Transport studies of protonated emeraldine polymer—A antigranulocytes polymeric metal system. Phys. Rev. B.

[122] Leite F.L., Alves W.F., Mir M., Mascarenhas Y.P., Herrmann P.S.P., Mattoso L.H.C., Oliveira O.N. (2008). Tem, xrd and afm study of poly(*O*-ethoxyaniline) films: New evidence for the formation of conducting islands. Appl. Phys. A: Mater. Sci. Process..

[123] Lux F., Hinrichsen G., Pohl M.M. (1994). Tem evidence for the existence of conducting islands in highly conductive polyaniline. J. Polym. Sci. Pt. B Polym. Phys.

[124] Knoll A., Magerle R., Krausch G. (2001). Tapping mode atomic force microscopy on polymers: Where is the true sample surface?. Macromolecules.

[125] Chen X., Roberts C.J., Zhang J., Davies M.C., Tendler S.J.B. (2002). Phase contrast and attraction-repulsion transition in tapping mode atomic force microscopy. Surf. Sci.

[126] Anczykowski B., Gotsmann B., Fuchs H., Cleveland J.P., Elings V.B. (1999). How to measure energy dissipation in dynamic mode atomic force microscopy. Appl. Surf. Sci.

[127] Yoshizawa H., Chen Y.L., Israelachvili J. (1993). Fundamental mechanisms of interfacial friction.1. Relation between adhesion and friction. J. Phys. Chem.

[128] Kitamura S., Iwatsuki M. (1995). Observation of 7x7 reconstructed structure on the silicon (111) surface using ultrahigh-vacuum noncontact atomic-force microscopy. Jpn. J. Appl. Phys. Part 2 Lett.

[129] Sokolov I.Y., Henderson G.S., Wicks F.J. (1999). Force spectroscopy in noncontact mode. Appl. Surf. Sci.

[130] Giessibl F.J. (1995). Atomic-resolution of the silicon (111)-(7x7) surface by atomic-force microscopy. Science.

[131] Uchihashi T., Sugawara Y., Tsukamoto T., Ohta M., Morita S., Suzuki M. (1997). Role of a covalent bonding interaction in noncontact-mode atomic-force microscopy on si(111)7x7. Phys. Rev. B.

[132] van Honschoten J.W., Tas N.R., Elwenspoek M. (2010). The profile of a capillary liquid bridge between solid surfaces. Am. J. Phys.

[133] Men Y.M., Zhang X.R., Wang W.C. (2009). Capillary liquid bridges in atomic force microscopy: Formation, rupture, and hysteresis. J. Chem. Phys.

[134] Lambert P., Régnier S Microworld Modeling in Vacuum and Gaseous Environments.

[135] Cappella B., Dietler G (1999). Force-distance curves by atomic force microscopy. Surf. Sci. Rep.

[136] Fearing R.S. Survey of Sticking Effects for Micro Parts Handling.

[137] Hao H.W., Baro A.M., Saenz J.J. (1991). Electrostatic and contact forces in force microscopy. J. Vac. Sci. Technol. B Microelectron. Process. Phenom.

[138] Hudlet S., Saint Jean M., Guthmann C., Berger J. (1998). Evaluation of the capacitive force between an atomic force microscopy tip and a metallic surface. Eur. Phys. J. B.

[139] Patil S., Kulkarni A.V., Dharmadhikari C.V. (2000). Study of the electrostatic force between a conducting tip in proximity with a metallic surface: Theory and experiment. J. Appl. Phys.

[140] Patil S., Dharmadhikari C.V. (2002). Investigation of the electrostatic forces in scanning probe microscopy at low bias voltages. Surf. Interface Anal.

[141] Smythe W.R. (1968). Static and Dynamic Electricity.

[142] Overbeek J.T.G., Sparnaay M.J. (1954). Classical coagulation. London-vanderwaals attraction between macroscopic objects. Discuss. Faraday Soc.

[143] Black W., Dejongh J.G.V., Overbeek J.T.G., Sparnaay M.J. (1960). Measurements of retarded vanderwaals forces. Trans. Faraday Soc.

[144] Ducker W.A., Senden T.J., Pashley R.M. (1992). Measurement of forces in liquids using a force microscope. Langmuir.

[145] Larson I., Drummond C.J., Chan D.Y.C., Grieser F. (1993). Direct force measurements between TiO_2_ surfaces. J. Am. Chem. Soc.

[146] Biggs S., Spinks G. (1998). Atomic force microscopy investigation of the adhesion between a single polymer sphere and a flat surface. J. Adhes. Sci. Technol.

[147] Butt H.J., Graf K., Kappl M (2003). Physics and Chemistry of Interfaces.

[148] Lennard-Jones J.E. (1931). Cohesion. Proc. Phys. Soc.

[149] Stifter T., Marti O., Bhushan B. (2000). Theoretical investigation of the distance dependence of capillary and van der waals forces in scanning force microscopy. Phys. Rev. B.

[150] Derjaguin B.V. (1934). Friction and adhesion. Iv. The theory of adhesion of small particles. Kolloid Zeits.

[151] Todd B.A., Eppell S.J. (2004). Probing the limits of the derjaguin approximation with scanning force microscopy. Langmuir.

[152] Hunter R (1989). Foundations of Colloid Science.

[153] French R.H., Cannon R.M., Denoyer L.K., Chiang Y.M. (1995). Full spectral calculation of nonretarded hamaker constants for ceramic systems from interband transition strengths. Solid State Ionics.

[154] Hutter J.L., Bechhoefer J. (1993). Manipulation of vanderwaals forces to improve image-resolution in atomic-force microscopy. J. Appl. Phys.

[155] Burnham N.A., Dominguez D.D., Mowery R.L., Colton R.J. (1990). Probing the surface forces of monolayer films with an atomic-force microscope. Phys. Rev. Lett.

[156] Landman U., Luedtke W.D., Burnham N.A., Colton R.J. (1990). Atomistic mechanisms and dynamics of adhesion, nanoindentation, and fracture. Science.

[157] Goodman F.O., Garcia N. (1991). Roles of the attractive and repulsive forces in atomic-force microscopy. Phys. Rev. B.

[158] Kirsch V.A. (2003). Calculation of the van der waals force between a spherical particle and an infinite cylinder. Adv. Colloid Interface Sci.

[159] Gradshteyn I.S., Ryzhik I.M. (1994). Table of Integrals, Series and Products.

[160] Casimir H.B.G., Polder D. (1948). The influence of retardation on the london-vanderwaals forces. Phys. Rev.

[161] Hartmann U. (1990). Manifestation of zero-point quantum fluctuations in atomic force microscopy. Phys. Rev. B.

[162] Wennerstrom H., Daicic J., Ninham B.W. (1999). Temperature dependence of atom-atom interactions. Phys. Rev. A.

[163] Ramos S.M.M., Charlaix E., Benyagoub A., Toulemonde M. (2003). Wetting on nanorough surfaces. Phys. Rev. E.

[164] Colak A., Wormeester H., Zandvliet H.J.W., Poelsema B. (2012). Surface adhesion and its dependence on surface roughness and humidity measured with a flat tip. Appl. Surf. Sci.

[165] Zhang X.L., Lu Y.J., Liu E.Y., Yi G.W., Jia J.H. (2012). Adhesion and friction studies of microsphere-patterned surfaces in contact with atomic force microscopy colloidal probe. Colloids Surf. A: Physicochem. Eng. Asp.

[166] Mizes H.A., Ott M., Eklund E., Hays D.A. (2000). Small particle adhesion: Measurement and control. Colloids Surf. A: Physicochem. Eng. Asp.

[167] Rimai D.S., DeMejo L.P. (1996). Physical interactions affecting the adhesion of dry particles. Annu. Rev. Mater. Sci.

[168] Hays D.A. (1994). Toner Adhesion. Proceedings of the Seventeenth Annual Meeting and the Symposium on Particle Adhesion.

[169] Matsusaka S. (2011). Control of particle tribocharging. KONA Powder Part. J.

[170] Horn R.G., Smith D.T. (1992). Contact electrification and adhesion between dissimilar materials. Science.

[171] Johnson K.L., Kendall K., Roberts A.D. (1971). Surface energy and contact of elastic solids. Proc. R. Soc. Lond. Ser. A: Math. Phys. Sci.

[172] Derjaguin B.V., Muller V.M., Toporov Y.P. (1975). Effect of contact deformations on adhesion of particles. J. Colloid Interface Sci.

[173] Harkins W.D. (1928). Surface energy and the orientation of molecules in surfaces as revealed by surface energy relations. Zeitschrift für Physikalische Chemie.

[174] Maugis D., Pollock H.M. (1984). Surface forces, deformation and adherence at metal microcontacts. Acta Metall.

[175] Skvarla J. (2001). Hydrophobic interaction between macroscopic and microscopic surfaces. Unification using surface thermodynamics. Adv. Colloid Interface Sci.

[176] Noy A (2007). Handbook of Molecular Force Spectroscopy.

[177] Tabor D. (1977). Surface forces and surface interactions. J. Colloid Interface Sci.

[178] Muller V.M., Yushchenko V.S., Derjaguin B.V. (1980). On the influence of molecular forces on the deformation of an elastic sphere and its sticking to a rigid plane. J. Colloid Interface Sci.

[179] Maugis D.J. (1992). Adhesion of spheres: The jkr-dmt transition using a dugdale model. J. Colloid Interface Sci.

[180] Johnson K.L., Greenwood J.A. (1997). An adhesion map for the contact of elastic spheres. J. Colloid Interface Sci.

[181] Xu D.W., Liechti K.M., Ravi-Chandar K. (2007). On the modified tabor parameter for the jkr-dmt transition in the presence of a liquid meniscus. J. Colloid Interface Sci.

[182] Fogden A., White L.R. (1990). Contact elasticity in the presence of capillary condensation. 1. The nonadhesive hertz problem. J. Colloid Interface Sci.

[183] Maugis D., Gauthiermanuel B. (1994). Jkr-dmt transition in the presence of a liquid meniscus. J. Adhes. Sci. Technol.

[184] Johnson K.L. (1998). Mechanics of adhesion. Tribol. Int.

[185] Carpick R.W., Agrait N., Ogletree D.F., Salmeron M. (1996). Variation of the interfacial shear strength and adhesion of a nanometer-sized contact. Langmuir.

[186] Lantz M.A., Oshea S.J., Welland M.E., Johnson K.L. (1997). Atomic-force-microscope study of contact area and friction on nbse2. Phys. Rev. B.

[187] Carpick R.W., Ogletree D.F., Salmeron M. (1999). A general equation for fitting contact area and friction vs load measurements. J. Colloid Interface Sci.

[188] Shi X.H., Zhao Y.P. (2004). Comparison of various adhesion contact theories and the influence of dimensionless load parameter. J. Adhes. Sci. Technol.

[189] Patrick D.L., Flanagan J.F., Kohl P., Lynden-Bell R.M. (2003). Atomistic molecular dynamics simulations of chemical force microscopy. J. Am. Chem. Soc.

[190] Rabinovich Y.I., Adler J.J., Ata A., Singh R.K., Moudgil B.M. (2000). Adhesion between nanoscale rough surfaces—i. Role of asperity geometry. J. Colloid Interface Sci.

[191] Rabinovich Y.I., Adler J.J., Ata A., Singh R.K., Moudgil B.M. (2000). Adhesion between nanoscale rough surfaces—ii. Measurement and comparison with theory. J. Colloid Interface Sci.

[192] Beach E.R., Tormoen G.W., Drelich J., Han R. (2002). Pull-off force measurements between rough surfaces by atomic force microscopy. J. Colloid Interface Sci.

[193] Zhou H.B., Gotzinger M., Peukert W. (2003). The influence of particle charge and roughness on particle-substrate adhesion. Powder Technol.

[194] Atherton A., Born G.V.R. (1972). Quantitative investigations of adhesiveness of circulating polymorphonuclear leukocytes to blood-vessel walls. J. Physiol.-Lond.

[195] Li Q., Rudolph V., Weigl B., Earl A. (2004). Interparticle van der waals force in powder flowability and compactibility. Int. J. Pharm.

[196] Li Q., Rudolph V., Peukert W. (2006). London-van der waals adhesiveness of rough particles. Powder Technol.

[197] Schaefer D.M., Carpenter M., Reifenberger R., Demejo L.P., Rimai D.S. (1994). Surface force interactions between micrometer-size polystyrene spheres and silicon substrates using atomic-force techniques. J. Adhes. Sci. Technol.

[198] Schaefer D.M., Carpenter M., Gady B., Reifenberger R., Demejo L.P., Rimai D.S. (1995). Surface-roughness and its influence on particle adhesion using atomic-force techniques. J. Adhes. Sci. Technol.

[199] Thoreson E.J., Martin J., Burnham N.A. (2006). The role of few-asperity contacts in adhesion. J. Colloid Interface Sci.

[200] Eichenlaub S., Kumar G., Beaudoin S. (2006). A modeling approach to describe the adhesion of rough, asymmetric particles to surfaces. J. Colloid Interface Sci.

[201] Kumar G., Beaudoin S. (2006). Undercut removal of micrometer-scale particles from surfaces. J. Electrochem. Soc.

[202] Cooper K., Gupta A., Beaudoin S. (2001). Simulation of the adhesion of particles to surfaces. J. Colloid Interface Sci.

[203] Kumar G., Smith S., Jaiswal R., Beaudoin S. (2008). Scaling of van der waals and electrostatic adhesion interactions from the micro- to the nano-scale. J. Adhes. Sci. Technol.

[204] Eichenlaub S., Gelb A., Beaudoin S. (2004). Roughness models for particle adhesion. J. Colloid Interface Sci.

[205] Lai L., Irene E.A. (1999). Area evaluation of microscopically rough surfaces. J. Vac. Sci. Technol. B.

[206] Liu D.L., Martin J., Burnham N.A. (2010). Which fractal parameter contributes most to adhesion?. J. Adhes. Sci. Technol.

[207] Segeren L., Siebum B., Karssenberg F.G., Van den Berg J.W.A., Vancso G.J. (2002). Microparticle adhesion studies by atomic force microscopy. J. Adhes. Sci. Technol.

[208] Jaiswal R.P., Kumar G., Kilroy C.M., Beaudoin S.P. (2009). Modeling and validation of the van der waals force during the adhesion of nanoscale objects to rough surfaces: A detailed description. Langmuir.

[209] Liu D.L., Martin J., Burnham N.A. Asme. Optimum Roughness for Minimum Adhesion.

[210] Karan S., Mallik B. (2008). Power spectral density analysis and photoconducting behavior in copper(ii) phthalocyanine nanostructured thin films. Phys. Chem. Chem. Phys.

[211] Kosaka P.M., Kawano Y., Petri D.F.S. (2007). Dewetting and surface properties of ultrathin films of cellulose esters. J. Colloid Interface Sci.

[212] Young T. (1805). An essay on the cohesion of fluids. Philos. Trans. R. Soc.

[213] Good R.J. (1966). Physical significance of parameters γ_c_, γ_s_ and Φ that govern spreading on adsorbed films. Society of the Chemical Industry Monographs.

[214] Good R.J. (1969). A thermodynamic analysis of formation of bilayer films. J. Colloid Interface Sci.

[215] Dupre A (1869). Theorie Mechanique de la Chaleur.

[216] Fowkes F.M. (1963). Additivity of intermolecular forces at interfaces .1. Determination of contribution to surface and interfacial tensions of dispersion forces in various liquids. J. Phys. Chem.

[217] Clint J.H., Wicks A.C. (2001). Adhesion under water: Surface energy considerations. Int. J. Adhes. Adhes.

[218] Berg J.C., Dillard D.A., Pocius A.V. (2002). Semi-Empiral Strategies for Predicting Adhesion. Adhesion Science and Engineering: Surfaces, Chemistry and Applications.

[219] Neumann A.W. (1974). Contact angles and their temperature dependence: Thermodynamic status, measurement, interpretation and application. Adv. Colloid Interface Sci.

[220] Kwok D.Y., Gietzelt T., Grundke K., Jacobasch H.J., Neumann A.W. (1997). Contact angle measurements and contact angle interpretation. 1. Contact angle measurements by axisymmetric drop shape analysis and a goniometer sessile drop technique. Langmuir.

[221] Kwok D.Y., Neumann A.W. (2000). Contact angle interpretation in terms of solid surface tension. Colloids Surf. A: Physicochem. Eng. Asp.

[222] Kwok D.Y., Neumann A.W. (2000). Contact angle interpretation: Re-evaluation of existing contact angle data. Colloids Surf. A: Physicochem. Eng. Asp.

[223] Jasper J.J. (1972). The surface tension of pure liquid compounds. J. Phys. Chem. Ref. Data.

[224] Vanoss C.J., Good R.J., Chaudhury M.K. (1988). Additive and nonadditive surface-tension components and the interpretation of contact angles. Langmuir.

[225] Clint J.H. (2001). Adhesion and components of solid surface energies. Curr. Opin. Colloid Interface Sci.

[226] van Oss C.J., Good R.J., Chaudhury M.K. (1987). Determination of the hydrophobic interaction energy —Application to separation processes. Sep. Sci. Technol.

[227] Owens D.K., Wendt R.C. (1969). Estimation of surface free energy of polymers. J. Appl. Polym. Sci.

[228] Aveyard R., Saleem S.M. (1976). Interfacial-tensions at alkane-aqueous electrolyte interfaces. J. Chem. Soc.-Faraday Trans. I.

[229] Erbil H.Y. (2006). Surfaces Chemistry of Solid and Liquid Interfaces.

[230] Bodner T., Behrendt A., Prax E., Wiesbrock F. (2012). Correlation of surface roughness and surface energy of silicon-based materials with their priming reactivity. Mon. Chem.

[231] Miller J.D., Veeramasuneni S., Drelich J., Yalamanchili M.R., Yamauchi G. (1996). Effect of roughness as determined by atomic force microscopy on the wetting properties of ptfe thin films. Polym. Eng. Sci.

[232] Drelich J., Tormoen G.W., Beach E.R. (2004). Determination of solid surface tension from particle-substrate pull-off forces measured with the atomic force microscope. J. Colloid Interface Sci.

[233] Tormoen G.W., Drelich J., Beach E.R. (2004). Analysis of atomic force microscope pull-off forces for gold surfaces portraying nanoscale roughness and specific chemical functionality. J. Adhes. Sci. Technol.

[234] Correa R., Saramago B. (2004). On the calculation of disjoining pressure isotherms for nonaqueous films. J. Colloid Interface Sci.

[235] Halsey G.D. (1949). Catalysis on non-uniform surfaces. J. Chem. Phys.

[236] He M.Y., Blum A.S., Aston D.E., Buenviaje C., Overney R.M., Luginbuhl R. (2001). Critical phenomena of water bridges in nanoasperity contacts. J. Chem. Phys.

[237] de Gennes P.G. (1985). Wetting: Statics and dynamics. Rev. Mod. Phys.

[238] Heslot F., Fraysse N., Cazabat A.M. (1989). Molecular layering in the spreading of wetting liquid drops. Nature.

[239] Israelachvili J.N. (1987). Solvation forces and liquid structure, as probed by direct force measurements. Acc. Chem. Res.

[240] Hu J., Xiao X.D., Salmeron M. (1995). Scanning polarization force microscopy—A technique for imaging liquids and weakly adsorbed layers. Appl. Phys. Lett.

[241] Hu J., Xiao X.D., Ogletree D.F., Salmeron M. (1995). Imaging the condensation and evaporation of molecularly thin-films of water with nanometer resolution. Science.

[242] Herminghaus S., Fery A., Reim D. (1997). Imaging of droplets of aqueous solutions by tapping-mode scanning force microscopy. Ultramicroscopy.

[243] Gil A., Colchero J., Luna M., Gomez-Herrero J., Baro A.M. (2000). Adsorption of water on solid surfaces studied by scanning force microscopy. Langmuir.

[244] Forcada M.L., Jakas M.M., Grasmarti A. (1991). On liquid-film thickness measurements with the atomic-force microscope. J. Chem. Phys.

[245] Maeda N., Israelachvili J.N., Kohonen M.M. (2003). Evaporation and instabilities of microscopic capillary bridges. Proc. Natl. Acad. Sci. USA.

[246] Adamson A.W. (1976). Physical Chemistry of Surfaces.

[247] Wei Z., Zhao Y.P. (2007). Growth of liquid bridge in afm. J. Phys. D-Appl. Phys.

[248] Aveyard R., Clint J.H., Paunov V.N., Nees D. (1999). Capillary condensation of vapours between two solid surfaces: Effects of line tension and surface forces. Phys. Chem. Chem. Phys.

[249] Ata A., Rabinovich Y.I., Singh R.K. (2002). Role of surface roughness in capillary adhesion. J. Adhes. Sci. Technol.

[250] Binggeli M., Mate C.M. (1994). Influence of capillary condensation of water on nanotribology studied by force microscopy. Appl. Phys. Lett.

[251] Thundat T., Zheng X.Y., Chen G.Y., Warmack R.J. (1993). Role of relative-humidity in atomic-force microscopy imaging. Surf. Sci.

[252] Hartholt G.P., Hoffmann A.C., Janssen L. (1996). Visual observations of individual particle behaviour in gas and liquid fluidized beds. Powder Technol.

[253] Obrien W.J., Hermann J.J. (1973). Strength of liquid bridges between dissimilar materials. J. Adhes.

[254] Miranda P.B., Xu L., Shen Y.R., Salmeron M. (1998). Icelike water monolayer adsorbed on mica at room temperature. Phys. Rev. Lett.

[255] Wiesendanger R., Guntherodt H.J. (1992). Scanning Tunneling Microscopy II.

[256] Utriainen M., Leijala A., Niinisto L., Matero R. (1999). Chemical imaging of patterned inorganic thin-film structures by lateral force microscopy. Anal. Chem.

[257] Jang J., Yang M., Schatz G. (2007). Microscopic origin of the humidity dependence of the adhesion force in atomic force microscopy. J. Chem. Phys.

[258] Yang G.L., Vesenka J.P., Bustamante C.J. (1996). Effects of tip-sample forces and humidity on the imaging of DNA with a scanning force microscope. Scanning.

[259] Fujihira M., Aoki D., Okabe Y., Takano H., Hokari H., Frommer J., Nagatani Y., Sakai F (1996). Effect of capillary force on friction force microscopy: A scanning hydrophilicity microscope. Chem. Lett.

[260] Binggeli M., Mate C.M. (1995). Influence of water-vapor on nanotribology studied by friction force microscopy. J. Vac. Sci. Technol. B.

[261] Tanaka M., Komagata M., Tsukada M., Kamiya H. (2008). Evaluation of the particle-particle interactions in a toner by colloid probe afm. Powder Technol.

[262] Shen Y.J., Nakajima M., Ahmad M.R., Kojima S., Homma M., Fukuda T. (2011). Effect of ambient humidity on the strength of the adhesion force of single yeast cell inside environmental-sem. Ultramicroscopy.

[263] Shi Q., Wong S.C., Ye W., Hou J.W., Zhao J., Yin J.H. (2012). Mechanism of adhesion between polymer fibers at nanoscale contacts. Langmuir.

[264] Chen S.C., Lin J.F. (2008). Detailed modeling of the adhesion force between an afm tip and a smooth flat surface under different humidity levels. J. Micromech. Microeng.

[265] Farshchi-Tabrizi M., Kappl M., Butt H.J. (2008). Influence of humidity on adhesion: An atomic force microscope study. J. Adhes. Sci. Technol.

[266] Yasuhisa A. (2000). The effect of relative humidity on friction and pull-off forces measured on submicron-size asperity arrays. Wear.

[267] Drelich J., Wang Y.U. (2011). Charge heterogeneity of surfaces: Mapping and effects on surface forces. Adv. Colloid Interface Sci.

[268] Yin X.H., Drelich J. (2008). Surface charge microscopy: Novel technique for mapping charge-mosaic surfaces in electrolyte solutions. Langmuir.

[269] Williams C.C., Hough W.P., Rishton S.A. (1989). Scanning capacitance microscopy on a 25 nm scale. Appl. Phys. Lett.

[270] Chang M.N., Chen C.Y., Wan W.W., Liang J.H. (2004). The influence of the annealing sequence on p(+)/n junctions observed by scanning capacitance microscopy. Appl. Phys. Lett.

[271] Williams C.C., Wickramasinghe H.K. (1991). Scanning chemical-potential microscope—A new technique for atomic scale surface investigation. J. Vac. Sci. Technol. B.

[272] Quirk J.P. (2003). Comments on “diffuse double-layer models, long-range forces, and ordering of clay colloids”. Soil Sci. Soc. Am. J.

[273] Liang Y., Hilal N., Langston P., Starov V (2007). Interaction forces between colloidal particles in liquid: Theory and experiment. Adv. Colloid Interface Sci.

[274] Leneveu D.M., Rand R.P., Parsegian V.A. (1976). Measurement of forces between lecithin bilayers. Nature.

[275] Qiu X.Y., Rau D.C., Parsegian V.A., Fang L.T., Knobler C.M., Gelbart W.M. (2011). Salt-dependent DNA-DNA spacings in intact bacteriophage lambda reflect relative importance of DNA self-repulsion and bending energies. Phys. Rev. Lett.

[276] Healy T.W., Homola A., James R.O., Hunter R.J. (1978). Coagulation of amphoteric latex colloids—Reversibility and specific ion effects. Faraday Discuss.

[277] de Souza E.F., Douglas R.A., Teschke O. (1997). Atomic force microscopic imaging in liquids: Effects of the film compressed between the substrate and the tip. Langmuir.

[278] Manhanty J., Ninham B.W. (1976). Dispersion Forces.

[279] Grasso D., Subramanian K., Butkus M., Strevett K., Bergendahl J. (2002). A review of non-dlvo interactions in environmental colloidal systems. Rev. Environ. Sci. Biotechnol.

[280] Pelin I.M., Piednoir A., Machon D., Farge P., Pirat C., Ramos S.M.M. (2012). Adhesion forces between afm tips and superficial dentin surfaces. J. Colloid Interface Sci.

[281] de Souza E.F., Ceotto G., Teschke O. (2001). Dielectric constant measurements of interfacial aqueous solutions using atomic force microscopy. J. Mol. Catal. A-Chem.

[282] Marra J. (1985). Direct measurements of attractive van der waals and adhesion forces between uncharged lipid bilayers in aqueous solutions. J. Colloid Interface Sci.

[283] Yaminsky V.V., Ninham B.W., Christenson H.K., Pashley R.M. (1996). Adsorption forces between hydrophobic monolayers. Langmuir.

[284] Bowen W.R., Williams P.M. (1996). The osmotic pressure of electrostatically stabilized colloidal dispersions. J. Colloid Interface Sci.

[285] Toikka G., Hayes R.A., Ralston J. (1996). Surface forces between spherical zns particles in aqueous electrolyte. Langmuir.

[286] Hamaker H.C. (1936). A general theory of lyophobic colloids i. Recueil des Travaux Chimiques des Pays-Bas.

[287] de Boer J.H. (1936). The influence of van der waals forces and primary bonds on binding energy, strengh and orientation, with special reference to some artifical resins. Trans. Faraday Soc.

[288] Derjaguin B.V. (1939). A theory of interaction of particles in presence of electric double layers and the stability of lyophobe colloids and disperse systems. Acta Physico-Chimica URSS.

[289] Derjaguin B.V., Landau L. (1941). Theory of the stability of strongly charged lyophobic sols and of the adhesion of strongly charged particles in solution of electrolytes. Acta Physico-Chimica URSS.

[290] Verwey E.J.W., Overbeek J.T.G. (1948). Theory of the Stability of Lyophobic Colloids.

[291] Christenson H.K. (1984). Dlvo (derjaguin-landau-verwey-overbeek) theory and solvation forces between mica surfaces in polar and hydrogen-bonding liquids. J. Chem. Soc.-Faraday Trans. I.

[292] Von Helmholtz H.L.F. (1879). Studies of electric boundary layers. Annalen der Physik.

[293] Conway B.E. (2006). Encyclopedia of Surface and Colloid Science.

[294] Gouy G. (1910). Constitution of the electric charge at the surface of an electrolyte. J. Phys.

[295] Chapman D.L. (1913). A contribution to the theory of electrocapillarity. Philos. Mag.

[296] Stern O. (1924). Zur theorie der elektrolytischen doppelschicht. Elektrochem.

[297] Debye P., Hückel E. (1923). Zur theorie der electrolyte. Zeitschrift fur Physik A.

[298] Luckham P.F. (2004). Manipulating forces between surfaces: Applications in colloid science and biophysics. Adv. Colloid Interface Sci.

[299] Parsegian V.A., Gingell D. (1972). Electrostatic interaction across a salt solution between 2 bodies bearing unequal charges. Biophys. J.

[300] Hogg R., Healy T.W., Fuerstenau D.W. (1966). Mutual coagulation of colloidal dispersions. Trans. Faraday Soc.

[301] Israelachvili J.N. (2011). Intermolecular and Surface Forces.

[302] Hartley P.G., Larson I., Scales P.J. (1997). Electrokinetic and direct force measurements between silica and mica surfaces in dilute electrolyte, solutions. Langmuir.

[303] Bevan M.A., Prieve D.C. (1999). Direct measurement of retarded van der waals attraction. Langmuir.

[304] Prieve D.C., Luo F., Lanni F. (1987). Brownian-motion of a hydrosol particle in a colloidal force-field. Faraday Discuss.

[305] Teschke O., de Souza E.F., Ceotto G. (1999). Double layer relaxation measurements using atomic force microscopy. Langmuir.

[306] Milling A., Mulvaney P., Larson I. (1996). Direct measurement of repulsive van der waals interactions using an atomic force microscope. J. Colloid Interface Sci.

[307] Lee S.W., Sigmund W.M. (2002). Afm study of repulsive van der waals forces between teflon af (tm) thin film and silica or alumina. Colloids Surf. A: Physicochem. Eng. Asp.

[308] Borato C.E., Leite F.L., Oliveira O.N., Mattoso L.H.C. (2006). Efficient taste sensors made of bare metal electrodes. Sens. Lett..

[309] Senden T.J., Drummond C.J. (1995). Surface-chemistry and tip sample interactions in atomic-force microscopy. Colloids Surf. A: Physicochem. Eng. Asp.

[310] Ninham B.W., Kurihara K., Vinogradova O.I. (1997). Hydrophobicity, specific ion adsorption and reactivity. Colloids Surf. A: Physicochem. Eng. Asp.

[311] Derjaguin B.V., Voropayeva T.N. (1964). Surface forces + stability of colloids + disperse systems. J. Colloid Sci.

[312] Clunie J.S., Goodman J.F., Symons P.C. (1967). Solvation forces in soap films. Nature.

[313] Abraham F.F. (1978). The interfacial density profile of a Lennard-Jones fluid in contact with a (100) Lennard-Jones wall and its relationship to idealized fluid-wall systems: A Monte-cCarlo simulation. J. Chem. Phys.

[314] Vanmegen W., Snook I. (1979). Solvent structure and solvation forces between solid bodies. J. Chem. Soc.-Faraday Trans. 2.

[315] Horn R.G., Israelachvili J.N. (1981). Direct measurement of structural forces between 2 surfaces in a non-polar liquid. J. Chem. Phys.

[316] Evans R., Parry A.O. (1990). Liquids at interfaces—What can a theorist contribute. J. Phys. Condes. Matter.

[317] Richetti P., Moreau L., Barois P., Kekicheff P. (1996). Measurement of the interactions between two ordering surfaces under symmetric and asymmetric boundary conditions. Phys. Rev. E.

[318] Kocevar K., Blinc R., Musevic I. (2000). Atomic force microscope evidence for the existence of smecticlike surface layers in the isotropic phase of a nematic liquid crystal. Phys. Rev. E.

[319] Zemb T., Parsegian V.A. (2011). Editorial overview: Hydration forces. Curr. Opin. Colloid Interface Sci.

[320] Parsegian V.A., Zemb T. (2011). Hydration forces: Observations, explanations, expectations, questions. Curr. Opin. Colloid Interface Sci.

[321] Ninham B.W. (1981). Surface forces - the last 30 a. Pure Appl. Chem.

[322] Kaggwa G.B., Nalam P.C., Kilpatrick J.I., Spencer N.D., Jarvis S.P. (2012). Impact of hydrophilic/hydrophobic surface chemistry on hydration forces in the absence of confinement. Langmuir.

[323] Cevc G. (1991). Hydration force and the interfacial structure of the polar surface. J. Chem. Soc. Faraday Trans.

[324] Leikin S., Parsegian V.A., Rau D.C., Rand R.P. (1993). Hydration forces. Annu. Rev. Phys. Chem.

[325] Ho R.Y., Yuan J.Y., Shao Z.F. (1998). Hydration force in the atomic force microscope: A computational study. Biophys. J.

[326] Molina-Bolivar J.A., Ortega-Vinuesa J.L. (1999). How proteins stabilize colloidal particles by means of hydration forces. Langmuir.

[327] Pashley R.M. (1982). Hydration forces between mica surfaces in electrolyte-solutions. Adv. Colloid Interface Sci.

[328] Grabbe A., Horn R.G. (1993). Double-layer and hydration forces measured between silica sheets subjected to various surface treatments. J. Colloid Interface Sci.

[329] Yoon R.H., Vivek S. (1998). Effects of short-chain alcohols and pyridine on the hydration forces between silica surfaces. J. Colloid Interface Sci.

[330] Churaev N.V., Derjaguin B.V. (1985). Inclusion of structural forces in the theory of stability of colloids and films. J. Colloid Interface Sci.

[331] Rand R.P., Fuller N., Parsegian V.A., Rau D.C. (1988). Variation in hydration forces between neutral phospholipid-bilayers - evidence for hydration attraction. Biochemistry.

[332] Valle-Delgado J.J., Molina-Bolivar J.A., Galisteo-Gonzalez F., Galvez-Ruiz M.J., Feiler A., Rutland M.W. (2005). Hydration forces between silica surfaces: Experimental data and predictions from different theories. J. Chem. Phys.

[333] Valle-Delgado J.J., Molina-Bolivar J.A., Galisteo-Gonzalez F., Galvez-Ruiz M.J., Feiler A., Rutland M. (2004). Interactions between bovine serum albumin layers adsorbed on different substrates measured with an atomic force microscope. Phys. Chem. Chem. Phys.

[334] Valle-Delgado J.J., Molina-Bolivar J.A., Galisteo-Gonzalez F., Galvez-Ruiz M.J., Feiler A., Rutland M.W. (2005). Existence of hydration forces in the interaction between apoferritin molecules adsorbed on silica surfaces. Langmuir.

[335] Paunov V.N., Kaler E.W., Sandler S.L., Petsev D.N. (2001). A model for hydration interactions between apoferritin molecules in solution. J. Colloid Interface Sci.

[336] Besseling N.A.M. (1997). Theory of hydration forces between surfaces. Langmuir.

[337] Rabinovich Y.I., Derjaguin B.V., Churaev N.V. (1982). Direct measurements of long-range surface forces in gas and liquid-media. Adv. Colloid Interface Sci.

[338] Peschel G., Belouschek P., Muller M.M., Muller M.R., Konig R. (1982). The interaction of solid-surfaces in aqueous systems. Colloid Polym. Sci.

[339] Klier K., Zettlemoyer A.C. (1977). Water at interfaces - molecular-structure and dynamics. J. Colloid Interface Sci.

[340] Derjaguin B.V. (1966). Effect of lyophile surfaces on properties of boundary liquid films. Discuss. Faraday Soc.

[341] Attard P., Batchelor M.T. (1988). A mechanism for the hydration force demonstrated in a model system. Chem. Phys. Lett.

[342] Trokhymchuk A., Henderson D., Wasan D.T. (1999). A molecular theory of the hydration force in an electrolyte solution. J. Colloid Interface Sci.

[343] Basu S., Sharma M.M. (1994). Effect of dielectric saturation on disjoining pressure in thin-films of aqueous-electrolytes. J. Colloid Interface Sci.

[344] Henderson D., Lozadacassou M. (1986). A simple theory for the force between spheres immersed in a fluid. J. Colloid Interface Sci.

[345] Paunov V.N., Binks B.P. (1999). Analytical expression for the electrostatic disjoining pressure taking into account the excluded volume of the hydrated ions between charged interfaces in electrolyte. Langmuir.

[346] Lyklema J. (1968). The structure of electrical double layer on porous surfaces. J. Electroanal. Chem.

[347] Vigil G., Xu Z.H., Steinberg S., Israelachvili J. (1994). Interactions of silica surfaces. J. Colloid Interface Sci.

[348] Kralchevsky P.A., Danov K.D., Basheva E.S. (2011). Hydration force due to the reduced screening of the electrostatic repulsion in few-nanometer-thick films. Curr. Opin. Colloid Interface Sci.

[349] Norrish K (1954). The swelling of montmorillonite. Discuss. Faraday Soc.

[350] Pashley R.M., Israelachvili J.N. (1981). A comparison of surface forces and interfacial properties of mica in purified surfactant solutions. Colloids Surf.

[351] Pashley R.M., Israelachvili J.N. (1984). Molecular layering of water in thin-films between mica surfaces and its relation to hydration forces. J. Colloid Interface Sci.

[352] Israelachvili J.N., Pashley R.M. (1983). Molecular layering of water at surfaces and origin of repulsive hydration forces. Nature.

[353] Horn R.G., Smith D.T., Haller W. (1989). Surface forces and viscosity of water measured between silica sheets. Chem. Phys. Lett.

[354] Claesson P., Carmonaribeiro A.M., Kurihara K. (1989). Dihexadecyl phosphate monolayers—Intralayer and interlayer interactions. J. Phys. Chem.

[355] Chapel J.P. (1994). Electrolyte species-dependent hydration forces between silica surfaces. Langmuir.

[356] Allen L.H., Matijević E. (1969). Stability of colloidal silica.I. Effect of simple electrolytes. J. Colloid Interface Sci.

[357] Yotsumoto H., Yoon R.H. (1993). Application of extended dlvo theory .1. Stability of rutile suspensions. J. Colloid Interface Sci.

[358] Yotsumoto H., Yoon R.H. (1993). Application of extended dlvo theory .2. Stability of silica suspensions. J. Colloid Interface Sci.

[359] Depasse J., Watillon A. (1970). Stability of amorphous colloidal silica. J. Colloid Interface Sci.

[360] Persson P.K.T., Bergenstahl B.A. (1985). Repulsive forces in lecithin glycol lamellar phases. Biophys. J.

[361] Duan J.M. (2009). Interfacial forces between silica surfaces measured by atomic force microscopy. J. Environ. Sci. -China.

[362] Christenson H.K., Claesson P.M. (1988). Cavitation and the interaction between macroscopic hydrophobic surfaces. Science.

[363] Rabinovich Y.I., Derjaguin B.V. (1988). Interaction of hydrophobized filaments in aqueous-electrolyte solutions. Colloids Surf.

[364] Horinek D., Serr A., Bonthuis D.J., Bostrom M., Kunz W., Netz R.R. (2008). Molecular hydrophobic attraction and ion-specific effects studied by molecular dynamics. Langmuir.

[365] Christenson H.K., Claesson P.M. (2001). Direct measurements of the force between hydrophobic surfaces in water. Adv. Colloid Interface Sci.

[366] Derjaguin B.V., Churaev N.V. (1974). Structural component of disjoining pressure. J. Colloid Interface Sci.

[367] Ruckenstein E., Churaev N. (1991). A possible hydrodynamic origin of the forces of hydrophobic attraction. J. Colloid Interface Sci.

[368] Yushchenko V.S., Yaminsky V.V., Shchukin E.D. (1983). Interaction between particles in a nonwetting liquid. J. Colloid Interface Sci.

[369] Podgornik R., Parsegian V.A. (1991). An electrostatic-surface stability interpretation of the hydrophobic force inferred to occur between mica plates in solutions of soluble surfactants. Chem. Phys.

[370] Tsao Y.H., Evans D.F., Wennerstrom H. (1993). Long-range attraction between a hydrophobic surface and a polar surface is stronger than that between 2 hydrophobic surfaces. Langmuir.

[371] Berard D.R., Attard P., Patey G.N. (1993). Cavitation of a lennard-jones fluid between hard walls, and the possible relevance to the attraction measured between hydrophobic surfaces. J. Chem. Phys.

[372] Wood J., Sharma R. (1995). How long is the long-range hydrophobic attraction?. Langmuir.

[373] Zhang J.H., Yoon R.H., Mao M., Ducker W.A. (2005). Effects of degassing and ionic strength on afm force measurements in octadecyltrimethylammonium chloride solutions. Langmuir.

[374] Podgornik R. (1989). Forces between surfaces with surface-specific interactions in a dilute electrolyte. Chem. Phys. Lett.

[375] Kekicheff P., Spalla O (1995). Long-range electrostatic attraction between similar, charge-neutral walls. Phys. Rev. Lett..

[376] Ishida N., Sakamoto M., Miyahara M., Higashitani K. (2000). Attraction between hydrophobic surfaces with and without gas phase. Langmuir.

[377] Stevens H., Considine R.F., Drummond C.J., Hayes R.A., Attard P. (2005). Effects of degassing on the long-range attractive force between hydrophobic surfaces in water. Langmuir.

[378] Teschke O., de Souza E.F. (2003). Measurements of long-range attractive forces between hydrophobic surfaces and atomic force microscopy tips. Chem. Phys. Lett.

[379] Chandler D. (2005). Interfaces and the driving force of hydrophobic assembly. Nature.

[380] Despa F., Berry R.S. (2007). The origin of long-range attraction between hydrophobes in water. Biophys. J.

[381] Parker J.L., Cho D.L., Claesson P.M. (1989). Plasma modification of mica—forces between fluorocarbon surfaces in water and a nonpolar liquid. J. Phys. Chem.

[382] Christenson H.K., Fang J.F., Ninham B.W., Parker J.L. (1990). Effect of divalent electrolyte on the hydrophobic attraction. J. Phys. Chem.

[383] Sirghi L., Nakagiri N., Sugimura H., Takai O. (2001). Analysis of atomic force curve data for mapping of surface properties in water. Jpn. J. Appl. Phys.

[384] Rabinovich Y.I., Yoon R.H. (1994). Use of atomic-force microscope for the measurements of hydrophobic forces. Colloids Surf. A: Physicochem. Eng. Asp.

[385] Rabinovich Y.I., Yoon R.H. (1994). Use of atomic-force microscope for the measurements of hydrophobic forces between silanated silica plate and glass sphere. Langmuir.

[386] Kokkoli E., Zukoski C.F. (1998). Interactions between hydrophobic self-assembled monolayers. Effect of salt and the chemical potential of water on adhesion. Langmuir.

[387] Freitas A.M., Sharma M.M. (2001). Detachment of particles from surfaces: An afm study. J. Colloid Interface Sci.

[388] Teschke O., de Souza E.F. (2003). Hydrophobic surfaces probed by atomic force microscopy. Langmuir.

[389] Teschke O., de Souza E.F. (2003). Electrostatic response of hydrophobic surface measured by atomic force microscopy. Appl. Phys. Lett.

[390] Yoon R.H., Ravishankar S.A. (1996). Long-range hydrophobic forces between mica surfaces in dodecylammonium chloride solutions in the presence of dodecanol. J. Colloid Interface Sci.

